# Spin–Vibronic Control of Intersystem Crossing in Iodine-Substituted Heptamethine Cyanines

**DOI:** 10.1021/acs.joc.3c00005

**Published:** 2023-05-05

**Authors:** Radek Tovtik, Eva Muchová, Lenka Štacková, Petr Slavíček, Petr Klán

**Affiliations:** † Department of Chemistry, Faculty of Science, Masaryk University, Kamenice 5, 625 00 Brno, Czech Republic; § RECETOX, Faculty of Science, Masaryk University, Kamenice 5, 625 00 Brno, Czech Republic; ‡ Department of Physical Chemistry, University of Chemistry and Technology, Prague, Technické 5, 166 28 Prague 6, Czech Republic

## Abstract

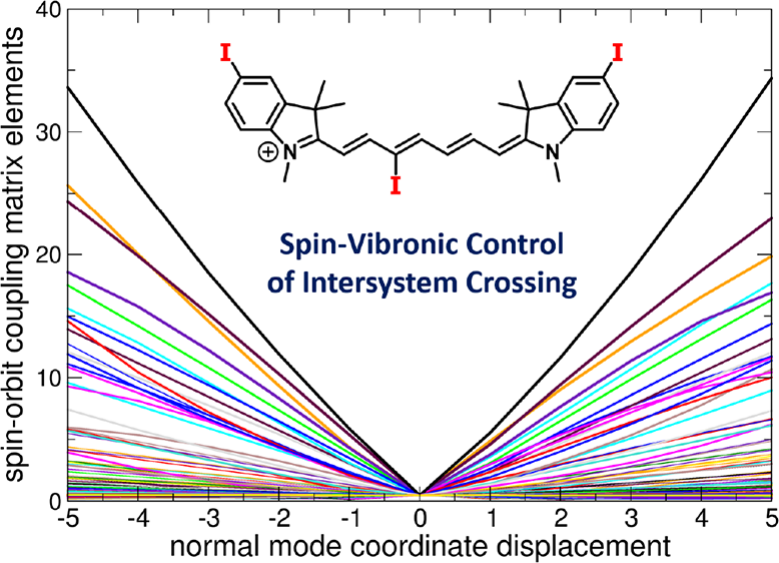

Spin–orbit coupling between electronic states of different multiplicity can be strongly coupled to molecular vibrations, and this interaction is becoming recognized as an important mechanism for controlling the course of photochemical reactions. Here, we show that the involvement of spin–vibronic coupling is essential for understanding the photophysics and photochemistry of heptamethine cyanines (Cy7), bearing iodine as a heavy atom in the C3′ position of the chain and/or a 3*H*-indolium core, as potential triplet sensitizers and singlet oxygen producers in methanol and aqueous solutions. The sensitization efficiency was found to be an order of magnitude higher for the chain-substituted than the 3*H*-indolium core-substituted derivatives. Our ab initio calculations demonstrate that while all optimal structures of Cy7 are characterized by negligible spin–orbit coupling (tenths of cm^–1^) with no dependence on the position of the substituent, molecular vibrations lead to its significant increase (tens of cm^–1^ for the chain-substituted cyanines), which allowed us to interpret the observed position dependence.

## Introduction

Heptamethine cyanines (Cy7) are small organic chromophores with strong absorption and emission in the near-infrared (NIR) region that are used in diverse biological applications, such as fluorescence probes,^1^ pH^2^ and metal cation^3−5^ sensors, and DNA^6^ and protein^7^ markers, or for tumor visualization^8^ and photocaging.^9,10^ The best-known Cy7 derivative, indocyanine green (ICG), is a fluorescent probe approved by the Federal Drug Administration (FDA)^11^ for various clinical applications.^12^ Some Cy7 derivatives, bearing heavy halogen atoms—usually on the indole core—have been reported to act as photosensitizers.^13−15^ Heavy atoms enhance intersystem crossing (ISC) due to strong spin–orbit coupling between the singlet and triplet states (heavy-atom effect, HAE).^16,17^ The ISC enhancement can also be achieved by introducing a covalently-linked radical species, such as 2,2,6,6-tetramethyl-1-piperidinyloxyl (TEMPO),^18^ J-aggregation,^19^ or charge transfer.^20,21^ Electron-donating substituents in the C4′ positions or electron-withdrawing groups in the C3′ position of the Cy7 chain were also shown to improve ISC, but the effect is relatively small.^22^ Triplet-excited Cy7s can be used as singlet oxygen generators in organic synthesis,^23^ wastewater treatment,^24^ or photodynamic therapy.^25^

Some of us have recently introduced a new approach to the synthesis of Cy7 derivatives substituted in the C3′–C5′ positions of the chain under mild conditions using a Zincke salt ring-opening reaction.^26^ This allowed us to investigate how the chain substitution modulates their photophysical properties, such as quantum yields of singlet oxygen formation, photodecomposition, and emission.^22^ It was demonstrated that the C3′-iodine substitution significantly increases the quantum yield of singlet oxygen production (Φ_Δ_).^22^

The cyanine dyes have also attracted the attention of theory; the electronic structure of cyanine dyes and their excited states have been modeled by many research groups for over three decades. Models ranging from the simplest yet surprisingly accurate particle-in-a-box model^27^ to current electronic structure models (typically at the density functional theory (DFT) level) have been used to explain the substituent and strong vibronic effects responsible for the characteristic asymmetric shape of the electronic spectra of cyanines. Despite the effort, the quantitative modeling of these spectra is still a challenge,^28−36^ as standard DFT methods fail to reproduce the exact position of the absorption bands. The reason for this unsuspected failure has been addressed by various research groups.^29,36−39^ Interestingly, the electronic states of interest show no significant multi-reference character, the overlap of the involved frontier orbitals is large, and the charge-transfer character is not present. Yet, the accurate peak position is not accessible via widely used DFT methods. From a qualitative point of view, inaccurate DFT energies are related to a particularly strong reorganization of the electron density upon excitation.^29,36,38^ ISC in excited cyanine dyes has been studied to a much lesser extent, and the treatment was typically insufficient to understand the photophysics.

**Figure 1:**
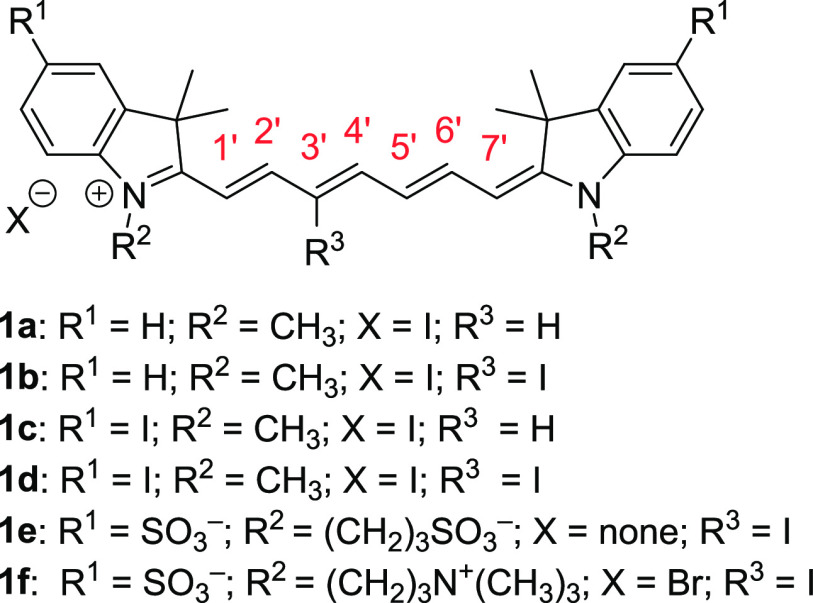
Cy7 derivatives studied in this work.

In this work, we synthesized several iodine-substituted Cy7 derivatives **1** (Figure 1) to study the effects of the substituent positions on ISC and singlet oxygen production in methanol and aqueous solutions to evaluate their potential application as photosensitizers. To interpret the experimentally observed results, we used quantum chemical methods focusing on the interaction between spin–orbit coupling and vibrational motion. The spin–vibronic coupling proved to be essential for a correct understanding of the ISC phenomena.^40^

## Results and Discussion

### Synthesis

**Scheme 1:**
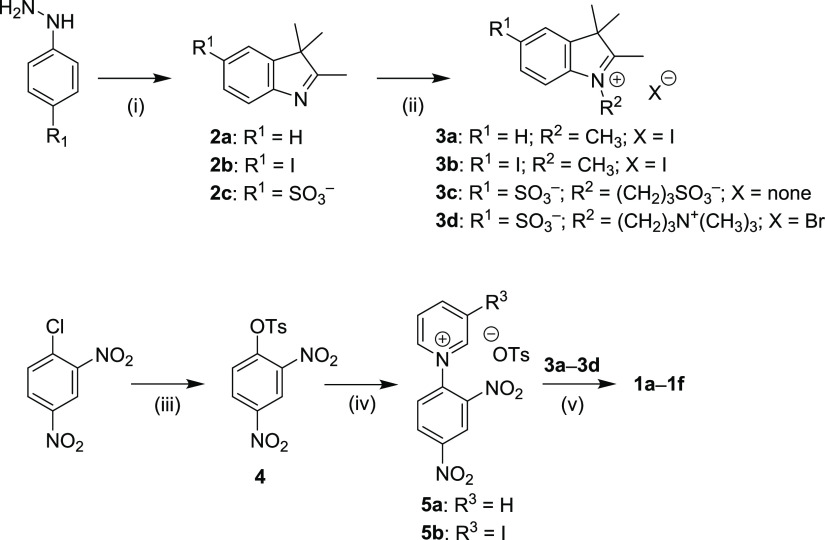
Synthesis of Cyanine Derivatives **1a**–**f**. (i) **2a**: Commercially Available; **2b**: 3-Methyl-2-butanone, H_2_SO_4_, Ethanol, Reflux, 78%; **2c**: 3-Methyl-2-butanone, Acetic Acid, Reflux, then NaOH, CH_3_OH + i-PrOH, Reflux, 85%; (ii) **3a**,**b**: CH_3_I, Toluene, 120 °C, 85–93%; **3c**: 1,3-Propanesultone, Toluene, Reflux, 32%; **3d**: 3-Bromo-*N*,*N*,*N*-trimethyl-1-propanaminium Bromide, NaI, CH_3_CN, 120 °C, 93%; (iii) **4**: TsCl, TEA, DCM, 20 °C, 85%; (iv) **5a** Pyridine, Toluene, Reflux, 90%; **5b**: 3-Iodopyridine, 120 °C, 87%; (v) **1a,b**: 4-Bromoaniline, NaOAc, Methanol, 20 °C, 75–76%; **1c**,**d**: 4-Bromoaniline, NaOAc, CH_3_OH, 45 °C, 28–33%; **1e**,**f**: 4-Aminobenzonitrile, NaOAc, CH_3_OH, 45 °C, 54–87%

Iodine atom-substituted heptamethine cyanines **1** were synthesized via ring-opening of the corresponding Zincke salts according to the reported procedure (Figure 1, Scheme 1).^26^ This methodology allowed us to prepare compounds **1a**–**f** containing iodine atoms in both the heptamethine (C3′ position) and 3*H*-indolium moieties. 3*H*-Indole derivatives **2a**–**c** were synthesized from the corresponding hydrazines via the Fischer indole synthesis, and substituted indolium compounds **3a**–**d** were obtained by alkylation of the nitrogen atom. The other precursors, *N*-2,4-dinitrophenylpyridinium (Zincke) salts **5a**,**b**, were prepared by the reaction of pyridine or 3-iodopyridine with 2,4-dinitrophenyl tosylate **4**. Finally, the synthesis of derivatives **1a**–**f** was carried out via ring-opening of an electron-deficient pyridinium core using 4-bromoaniline and the subsequent condensation with the corresponding indolium heterocycle. For **1d**,**e**, 4-bromoaniline as a nucleophile provided cyanines only in low yields; higher chemical yields (86 and 54%, respectively) were obtained using 4-aminobenzonitrile.

### Absorption and Emission Spectroscopy

**Figure 2:**
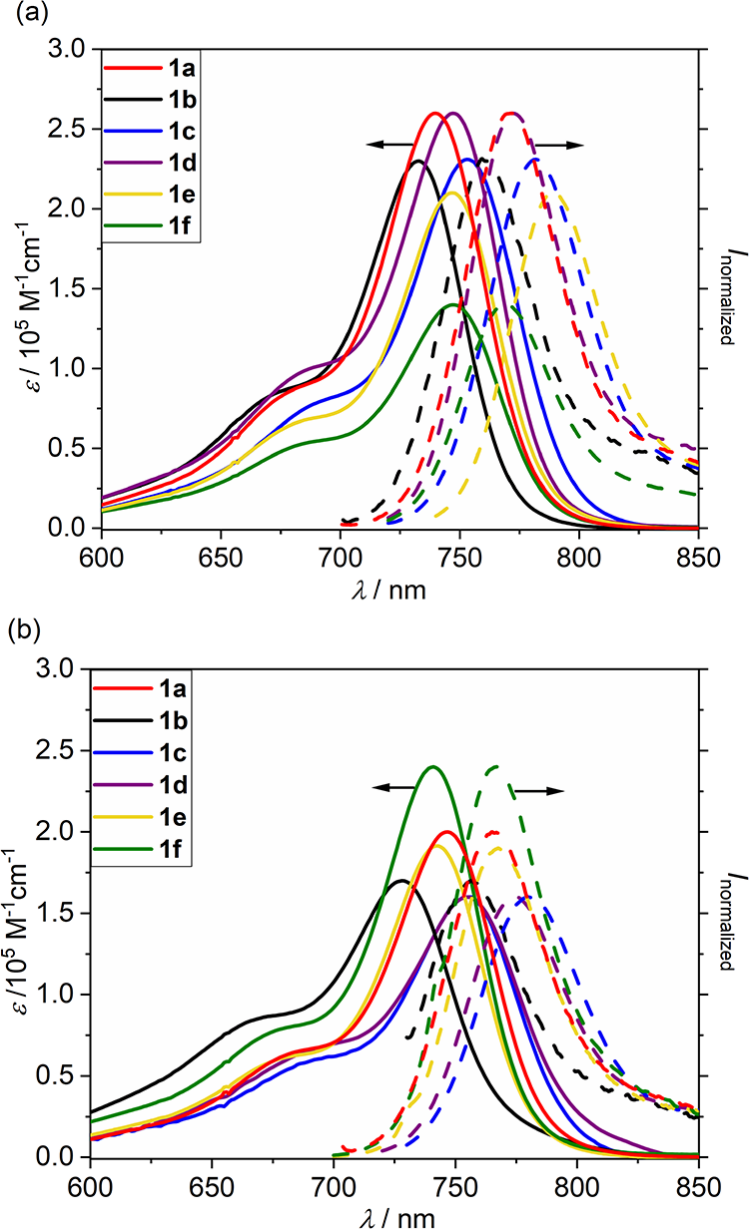
Absorption (solid line) and emission (dashed line) spectra of **1a**–**f** in (a) methanol and (b) PBS.

The absorption and emission spectra of **1a**–**f** were determined in both methanol and phosphate-buffered saline (PBS, pH 7.4, *c* = 10 mM, *I* = 100 mM). The major absorption bands (λ_max_^abs^) are located in the NIR regions of 733–753 and 728–749 nm in methanol and PBS, respectively (Table 1, Figure 2, Figures S1 and S2). In general, the molar absorption coefficients (ε_max_) in PBS are smaller than those in methanol. The compounds exhibit negative solvatochromism typical for Cy7 derivatives^41^ except for **1f**, which was also observed for other Cy7 derivatives bearing sulfonate and trimethylammonium groups.^42^ Compounds **1c** and **1d** form J- and H-aggregates in an aqueous solution; DMSO was thus used to suppress aggregation (Figure S3). Iodine substituents may be responsible for the increased tendency to form aggregates.^43^ Aggregation was also enhanced when other compounds (e.g., rose bengal used as a singlet oxygen generator; Figure S3c) were added to the aqueous solution. On the other hand, **1e** and **1f**, bearing sulfonate groups,^44^ did not aggregate in aqueous solutions. The emission (λ_max_^em^) in the range of 750–789 nm is responsible for small Stokes shifts (Δν̃) in agreement with the published data.^45^ The effect of the iodine substituents on the absorption and emission spectra is relatively small. The chain substitution with electron-withdrawing^46^ iodine is related to an insignificant hypsochromic shift,^22^ whereas the substitution on the indole core shows an opposite effect (Table 1).

**Table 1:** Absorption and Emission Properties of Cy7 Derivatives in Methanol and PBS

Cy7	solvent	λ_max_^abs^(nm)^a^	ε_max_ (10^5^ M^–1^ cm^–1^)^b^	λ_max_^em^ (nm)^c^	Δν̃ (cm^–1^)^d^	Φ_f_^e^	Φ_f_ε_max_^e^
**1a**	CH_3_OH	740	2.6 ± 0.1	772	560	0.24^f^	62,000
**1b**	CH_3_OH	733	2.3^f^	757	433	0.081 ± 0.020	19,000
**1c**	CH_3_OH	753	2.3 ± 0.1	782	492	0.16 ± 0.02	41,000
**1d**	CH_3_OH	747	2.6 ± 0.2	772	434	0.085 ± 0.020	20,000
**1e**	CH_3_OH	747	2.1 ± 0.1	789	713	0.103 ± 0.020	22,000
**1f**	CH_3_OH	744	1.4 ± 0.1^g^	769	437	0.098 ± 0.020	13,000
**1a**	PBS	736	2.0 ± 0.1	765	515	0.06^h^	12,000
**1b**	PBS	728	1.7^f^	763	630	0.018 ± 0.020	3000
**1c**	PBS	749^i^	1.6 ± 0.2^i^	780^i^	531	0.054 ± 0.020^i^	8700^i^
**1d**	PBS	746^j^	1.6 ± 0.1^j^	775^j^	629	0.041 ± 0.020^j^	6500^j^
**1e**	PBS	742	1.9 ± 0.1	768	456	0.064 ± 0.020	12,000
**1f**	PBS	741	2.4 ± 0.2	767	457	0.067 ± 0.020	16,000

a Absorption maxima λ_abs,max_.

b Molar absorption coefficients ε_max_ (10^5^ M^–1^ cm^–1^).

c Emission maxima λ_em,max_ (nm).

d Stokes shifts Δν̃ (cm^–1^).

e Fluorescence quantum yields Φ_f_ and brightness Φ_f_ε_max_ (M^–1^ cm^–1^) were obtained in the given solvent. PBS: pH = 7.4, *I* = 0.1 mM.

f Ref (22).

g 0.1% DMSO.

h Ref (47).

i PBS + 20% DMSO.

j PBS + 25% DMSO.

In general, Cy7 derivatives are known to exhibit low fluorescence quantum yields (Φ_f_),^22,48,49^ especially in water,^50^ although they still exhibit high molecular brightness (Φ_f_ε_max_) thanks to the large ε_max_ values. Iodo substitution led to the expected suppression of fluorescence by an order of magnitude thanks to enhanced ISC in both solvents (see later). For example, Φ_f_ ∼ 0.08 found for **1b** and **1d** in methanol is lower than that of unsubstituted Cy7 **1a** (Φ_f_ = 0.24^47^) by a factor of 2.5. Fluorescence was further suppressed in PBS (Φ_f_ = 0.04–0.07), the values which are comparable to that of **1a** in water (Φ_f_ = 0.06).^47^

### Characterization of the Excited States

**Figure 3:**
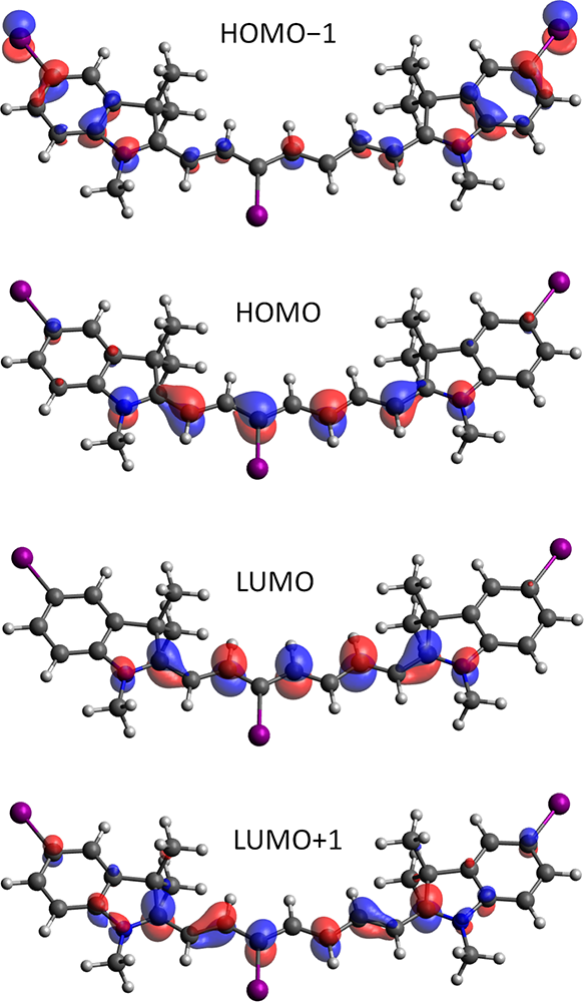
Molecular orbitals involved in the electronic transitions at the CAM-B3LYP/def2-TZVP level in water for **1d**. The contour threshold is set to 0.05 a.u.

To understand the photophysics of the studied dyes, we first had to characterize the relevant electronic states. The electronic absorption spectra of **1a**–**d** were calculated at the time-dependent density functional theory (TDDFT) level (full TDDFT and with the Tamm–Dancoff approximation, TDA) with the CAM-B3LYP functional. In all cases, the vertical electronic absorption is dominated by the transition between the highest occupied (HOMO) and lowest unoccupied (LUMO) molecular orbitals to give the S_1_ state, which exhibits the highest oscillator strength values, as also found for differently substituted cyanine dyes before.^22^ Both HOMO and LUMO are delocalized over the molecule. The vertical excitation energies are collected in Table 2; the corresponding molecular orbitals (for **1d**) are depicted in Figure 3. The first two triplet states have a mixed HOMO−1 → LUMO and HOMO → LUMO + 1 character. The experimentally observed vibronic shoulder of cyanine dyes (Figure 2) has already been interpreted: the 0–0 transition dominates the transition.^22,29,32,37−39,51^ We also compared the values calculated with and without TDA (Table 2). The values for singlet states obtained within TDA are systematically higher because cyanines are systems where de-excitation for a linear response of the density matrix is important. As pointed out by many authors before,^29,32,35,39^ the first π → π* singlet excitation energies are systematically overestimated by TDDFT methods (compare the data in Table 2 and the experimental data in Table 1). The agreement can be improved by techniques proposed in many previous works.^28−36^ However, in this work, we do not focus on quantitative modeling of the absorption spectra but on understanding ISC and the related rates and the dependence of ISC on vibrations.

**Table 2:** Excitation Energies in eV (Nm) for Molecules **1a**–**d** Calculated for Minimal Structures at the TDDFT CAM-B3LYP/def2-TZVP Level in Water with or without TDA^a^

	TDDFT	TDA	ΔSCF	TDDFT	TDA	TDDFT	TDA
Cy7	S_1_	S_1_	T_1_	T_1_	T_1_	T_2_	T_2_
**1a**	2.380 (521)	2.580 (481)	1.150 (1078)	0.881 (1407)	1.231 (1007)	2.442 (508)	2.574 (482)
**1b**	2.240 (553)	2.589 (479)	1.348 (920)	1.067 (1162)	1.335 (929)	2.456 (505)	2.581 (480)
**1c**	2.146 (578)	2.527 (491)	1.208 (1026)	0.875 (1417)	1.228 (1010)	2.435 (509)	2.555 (485)
**1d**	2.211 (561)	2.350 (528)	1.337 (927)	1.055 (1175)	1.369 (906)	2.437 (509)	2.594 (478)

a Triplet T_1_ energy was estimated as the energy difference between singlet and first triplet states. ΔSCF refers to the difference between the energy of the ground states and the energy of the lowest state of triplet multiplicity.

In contrast to the singlet states, TDA provides higher excitation energies for the triplet states that are supposedly more accurate.^52^ The TDA values of the triplet excitation energy for the T_1_ state are also closer to the ΔSCF values. The S/T energy gap was studied in detail by Zhekova and coworkers,^39^ who showed that the gap is systematically exaggerated by TDDFT methods independently of the DFT functional used. Table 2 shows that the S_1_/T_1_ energy gap in molecules **1a**–**d** is approximately 1 eV. Considering only vertical excitation, we suggest two possible ISC channels: (i) a nonradiative transition from bright S_1_ to T_1_ by spin–orbit coupling of S_1_ to higher vibrational levels of T_1_, or (ii) spin–orbit coupling to the higher triplet state T_2_ followed by a rapid internal conversion from T_2_ to T_1_. In both scenarios, the most important factors are the spin–orbit coupling (SOC) matrix elements (SOCMEs) and the energy gap between the given pair of states.

We can obtain more information about the plausible mechanism by analyzing the S_1_ state. The optimized geometries are collected in Table S3, and their overlap with the corresponding ground state structures is provided in Figure S46. As can be inferred from the structures, the geometry changes are relatively small in all cases; the changes are most profound in **1a** and **1c**. The TDDFT excitation energies at the S_1_ minimum are listed in Table S4. The T_2_ state energy is systematically lower than the S_1_ energy for the S_1_ minimum structure. Based on the energies of the states, we assume that after excitation to S_1_, the most relevant ISC channel involves SOC to the higher triplet state T_2_, followed by a rapid internal conversion from T_2_ to T_1_. The states primarily relevant for the ISC are the S_1_ and T_2_ states.

### Production of ^1^O_2_ and Photochemical Stability

Triplet-excited cyanines sensitize oxygen (^3^O_2_) to give singlet oxygen (^1^O_2_) and other reactive oxygen species.^53^ The quantum yield of ^1^O_2_ production (Φ_Δ_) is related to an ISC efficiency (Φ_isc_); the heavy atom substitution of a dye usually improves Φ_isc_ but also shortens the triplet lifetime,^54^ causing reduced quenching by O_2_. The iodine chain substitution in **1b** led to an increase in Φ_Δ_ in methanol (0.12) by more than an order of magnitude compared to Φ_Δ_ = 0.0095 found for unsubstituted derivative **1a** (Table 3). On the other hand, only a marginal increase (Φ_Δ_ = 0.018) was observed for **1c** with the I-substituted indolium core. A higher efficiency found for doubly I-substituted **1d** indicates the additivity of HEA (Φ_Δ_ = 0.13), as also observed before.^55^ Water-solubilizing groups in **1e** and **1f** did not affect the Φ_Δ_ values significantly. The effect of an iodide counterion on the ^1^O_2_ formation has been reported to be negligible.^22,56^

**Table 3:** Quantum Yields of Photodecomposition and Production of Singlet Oxygen and Rate Constants for the Reaction of Cy7 with ^1^O_2_

Cy7	solvent	Φ_Δ_^a^	Φ_dec_ (10^–4^)^b^	*k*_r_ (10^7^ M^–1^ s^–1^)^c^
**1a**	methanol	0.0095 ± 0.0005 (0.0089^d^)	0.031^d^	
**1b**		0.12 ± 0.01 (0.17^d^)	0.12 ± 0.02	
**1c**		0.018 ± 0.005	0.047 ± 0.011	
**1d**		0.13 ± 0.01	0.11 ± 0.02	
**1e**		0.16 ± 0.01	0.084 ± 0.010	
**1f**		0.11 ± 0.01	2.3 ± 0.3	
**1a**	PBS	n.d.	2.2^d^	
**1b**		n.d.	84^d^	6.87 ± 0.17^e^
**1c**		n.d.	63 ± 8^f^	5.38 ± 0.1.7^g^
**1d**		n.d.	173 ± 2^f^	1.63 ± 0.01^g^
**1e**		0.13 ± 0.01^h^	14 ± 2^f^	3.48 ± 0.11^h^
**1f**		0.25 ± 0.03^h^	8.0 ± 0.3^f^	2.86 ± 0.056^h^

a Quantum yield of singlet oxygen production; n.d. = not determined (compounds are not soluble); determined using 1,3-diphenylisobenzofuran as a ^1^O_2_ trap and methylene blue as a standard sensitizer.

b Quantum yield of photodecomposition (disappearance).

c Bimolecular rate constants of the reaction of Cy7 derivatives with singlet oxygen.

d Ref (22).

e 40% DMSO + 60% water.

f In water.

g 70% DMSO + 30% water.

h In PBS (pH 7.4, *c* = 10 mM, *I* = 100 mM).

We did not observe any significant decrease in the Φ_Δ_ values for water-soluble derivatives **1e** and **1f** in PBS (pH 7.4, *c* = 10 mM, *I* = 100 mM) solutions (Table 3). Indeed, polar Cy7 substituents have been shown by Burgess and coworkers to affect Φ_Δ_ only marginally.^57^ Their work reported that a Cy7 derivative bearing two iodine atoms in the indolium groups and one *meso* (C4′) chlorine atom exhibits Φ_Δ_ in the range of 0.59–0.79, which are significantly higher values than the maximum Φ_Δ_ found for C4′/indolium-substituted derivatives studied in our work. Those Φ_Δ_ values were calculated using ICG as a reference sensitizer with Φ_Δ_ (PBS) = 0.077. However, this number is an order of magnitude higher than that measured by Pandey and coworkers (Φ_Δ_ (ICG, methanol) = 0.008).^58^ In such a case, the Φ_Δ_ values for the reported *meso* (C4′) chlorine Cy7 derivatives^55^ would be similar to those reported in this work (Table 3), although we cannot directly compare Φ_Δ_ values obtained in methanol and PBS. Because of this controversy, we chose methylene blue as a reference sensitizer^59,60^ for our study.

Cyanine dyes are known to be chemically degraded (photobleaching) by regioselective oxidative fragmentation with singlet oxygen^61^ or via an electron transfer mechanism.^62,63^ The extent of photodecomposition depends, among others, on the type of solvent and the length of the cyanine chain. Cy7 derivatives tend to undergo photobleaching more efficiently than the shorter Cy5 or Cy3 analogues.^64^ To evaluate the photostability of Cy7 derivatives, quantum yields of decomposition (i.e., disappearance; Φ_dec_) were measured under different experimental conditions (Table 3). The Φ_Δ_ values were two to three orders of magnitude smaller than those in methanol and water; compound **1f** was the most reactive in methanol but relatively persistent in water. It has already been reported that cyanine polar groups increase their photostability.^65^ The results in Table 3 demonstrate that Cy7, in general, are relatively resistant to the presence of ^1^O_2_.

HRMS analyses of the irradiated mixtures of **1b** and **1c** in methanol suggested that the major degradation process detectable by HRMS is monodeiodination (Figures S50 and S51; a partial loss of iodine was also found to occur in the HRMS analyses of **1c**). Photoinduced homolysis of the sp^2^ carbon–halogen bond has been known since the 1960s.^66^ Homolysis requires the energy of the productive excited state to be greater than the corresponding bond dissociation energy (BDE).^16^ The C–I BDE of ∼65 kcal mol^–1^^67^ is too high for the Cy7 triplet energies (∼29 kcal mol^–1^; Table 2), but the S_1_ energy of ∼58 kcal mol^–1^ (Table 2) can be sufficiently high. Therefore, in addition to photobleaching related to the reaction of the heptamethine chain with ^1^O_2_ resulting in a complex chain degradation (see above), deiodination occurs from either a singlet excited state or via an alternative mechanism other than direct homolysis.^66^ Because photodegradation of I-containing cyanines **1** is much less efficient than sensitization (Table 3), we did not study these processes further.

Bimolecular rate constants of photooxygenation of Cy7 with ^1^O_2_ (*k*_r_), produced by rose bengal oxygen sensitization, were measured in aqueous solutions to evaluate the specific reactivities of cyanines (Table 3). DMSO was added to the solutions to increase the solubilities of Cy7 derivatives and the sensitizer (Figure S3c; see above). The relatively high rate constants cannot be directly compared because the DMSO content affects the ^1^O_2_ lifetime;^68^ however, we can conclude that improved solvation of the delocalized Cy7 cation in water enhances the nucleophilicity of cyanine toward the addition of ^1^O_2_, as also previously reported.^22^

### ISC and Spin–Vibronic Effects

We show below that the experimentally observed ^1^O_2_ production rates (Table 3) are not consistent with the calculated values of the SOCs in the optimal geometry. However, the molecular vibrations accessible even within the zero-point motion enhance the averaged value of the SOCs by an order of magnitude (leading to an increase in the ISC rate by two orders of magnitude) due to orbital mixing. This effect is called spin–vibronic coupling. In the following paragraphs, we explain this phenomenon in detail.

We can start with Fermi’s golden rule, expressing the rate of population transfer *k* as
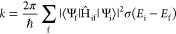
1where Ψ_i_ and Ψ_f_ are the wave functions of the initial and final states, Ĥ_if_ is the Hamiltonian describing the coupling, and *E*_i_ and *E*_f_ are energies of the initial and final states, respectively. For ISC, the Hamiltonian Ĥ_SO_ describes SOC. If the SOC is independent of vibrational motion, the equation simplifies to
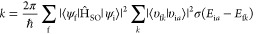
2where |⟨υ_f*k*_ | υ_i*a*_⟩|^2^ is the measure of the vibrational state overlap. Within this approach, we can calculate the SOCMEs as a qualitative estimate of the ISC rate constants and propose that SOC is driven by the electronic character of the states as suggested by El-Sayed rules (the coupling is effective if the spin change is accompanied by a change in angular momentum).^69,70^ Therefore, we calculated the SOCMEs for **1a**–**d** in their optimal ground state structures (Table 4) and the S_1_ optimized structures (Table S5). The calculated values are small (maximum tenths of cm^–1^) and almost the same for all studied Cy7 derivatives in both the ground state and the S_1_ state minima. Another important factor in eq 2 is the energy gap between the states, which is approximately the same for all studied molecules (Table 2 and Table S4). Apparently, the calculated data for the structures in the ground and S_1_ state minimum geometries, but neither SOCMEs nor energy gap, can explain an order of magnitude increase in the quantum yield of singlet oxygen production observed for **1b** and **1d**.

**Table 4:** SOCMEs in cm^–1^ for **1a**–**1d** Calculated for the Optimal Structures at the CAM-B3LYP/def2-TZVP and def2-SVP Levels in Water

	SOCME T_2_-S_1_		SOCME T_1_-S_1_	
Cy7	def2-TZVP	def2-SVP	def2-TZVP	def2-SVP
**1a**	0.000	0.000	0.000	0.000
**1b**	0.244	0.472	0.037	0.079
**1c**	0.088	0.103	0.020	0.030
**1d**	0.085	0.073	0.020	0.010

**Figure 4:**
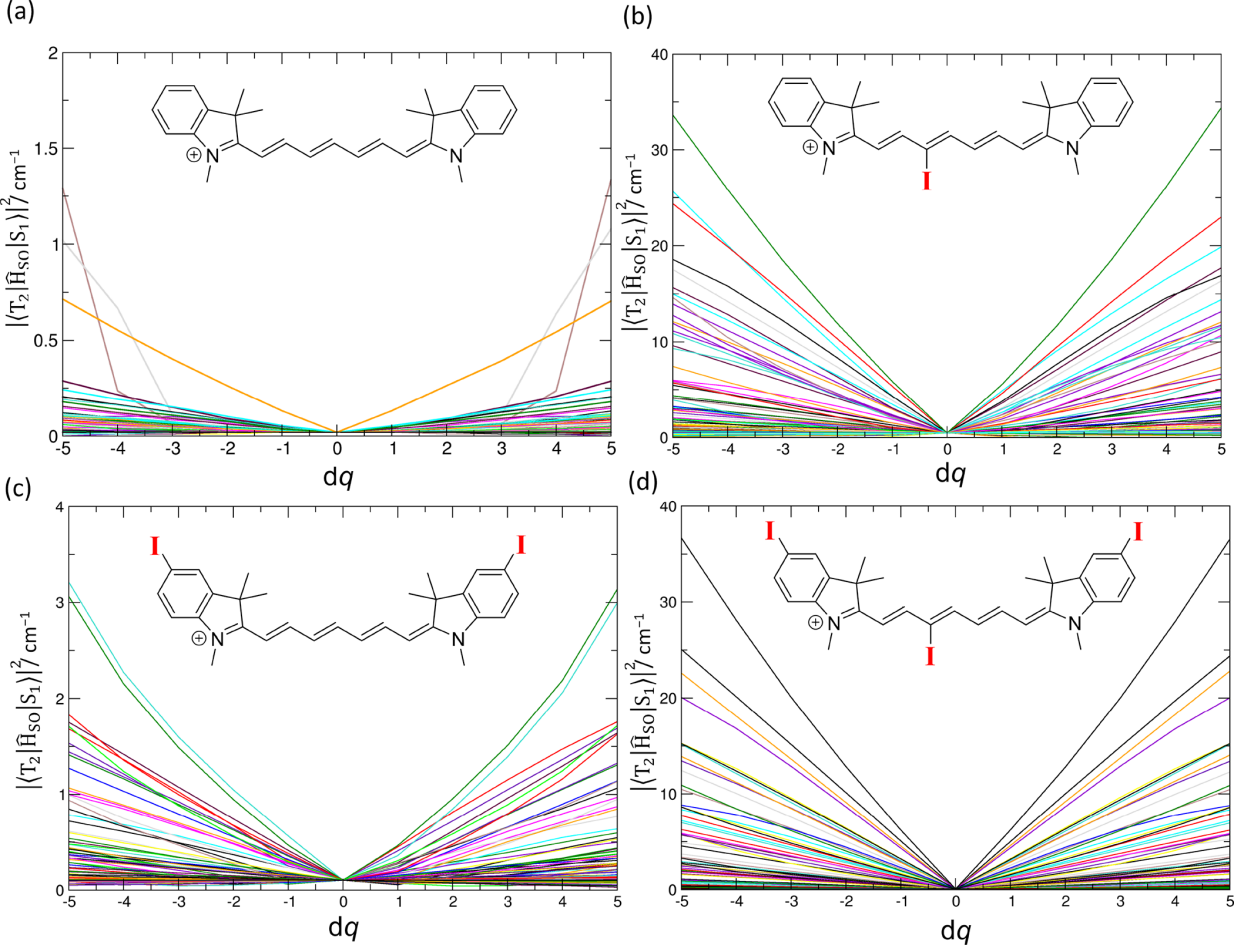
Spin–orbit coupling matrix elements (SOCMEs) between the T_2_ and S_1_ states for (a) **1a**, (b) **1b**, (c) **1c**, and (d) **1d** at CAM-B3LYP/def2-SVP in water as a polarizable continuum along all normal modes (186 in total) *q_i_* (of the optimized ground state geometry). The d*q* values are given in units of a dimensionless normal-mode coordinate displacement. The displacements with d*q* up to 2–3 are populated within the zero point motion.

**Figure 5:**
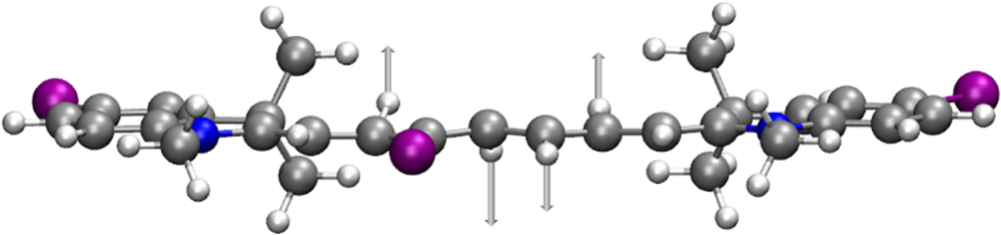
Schematic visualization of the out-of-plane normal mode ω_80_ for **1d** (941.93 cm^–1^) exhibiting the most significant SOCMEs values (at the CAM-B3LYP/def2-SVP level).

However, as Albrecht suggested in his work,^71^ there are other mechanisms for mixing the triplet and singlet states, such as vibrational SOC, in which the size of SOCMEs depends on the motion along a particular vibrational coordinate *q_i_*.^40^ The spin–orbit interaction, including the vibrational SOCs, can be calculated, for instance, within the framework of the first-order perturbation theory as
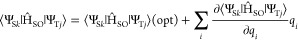
3where Ĥ_SO_ is the SOC operator and Ψ_S_ and Ψ_T_ are the wave functions of the singlet and triplet states. Along these lines, we calculated the SOCMEs for all vibrational coordinates in the ground-state geometries (see Figure 4 for T_2_ and S_1_ and Figure S47 for T_1_ and S_1_). Figure 4 shows the changes of SOCMEs along all 186 vibrational modes for **1a**–**1d**; the point d*q* = 0 corresponds to the optimized structure. The SOCMEs are very small (<1 cm^–1^) for the optimal structures **1a**–**1d**. The motion along several vibrational coordinates is associated with a substantial increase in the SOCMEs for **1a**–**1d**. However, the increase is an order of magnitude higher only for **1b** and **1d**, that is, for molecules bearing the iodine substituent attached to the heptamethine chain. The out-of-plane vibrations ω_73_ for **1b** (950.23 cm^–1^) and ω_80_ for **1d** (941.93 cm^–1^; Figure 5) show the most significant SOCMEs values (at the CAM-B3LYP/def2-SVP level). Note that the displacements in d*q* on the order of several units are accessible within the zero-point energy motion, irrespective of the vibrational frequency. A significant increase in SOCMEs connected to the out-of-plane vibrational modes was also reported for porphyrins^72,73^ or psoralene.^74^

In the optimal geometry, the iodine orbitals are perpendicular to the molecular plane and do not contribute to the frontier molecular orbitals (Figure 3). Once the system is distorted out of the plane, the iodine orbitals mix in the frontiers orbitals (Figure S48). The selection rules for SOC are lifted because of the possible mixing of the orbitals involved in the *n*,π* and π,π* states; as a result, the SOCMEs ⟨Ψ_S1_ | Ĥ_SO_ | Ψ_T2_⟩ adopt non-vanishing values. The atomic orbital contributions can be quantitatively evaluated in terms of Löwdin population analysis, where the population of a molecular orbital is projected into the minimal basis set of atomic orbitals of individual atoms. According to the analysis, the iodine atomic orbitals in the 3*H*-indolium moieties do not contribute to the frontier orbitals, whereas in the C3′ position, their contribution is up to a few percent (4% for HOMO), which affects ISC along the vibrational coordinates.

The change in the TDDFT with TDA energies of the S_1_, T_1_, and T_2_ states and energy differences along vibrational modes are shown in Figure S49. The figure demonstrates that the energy varies along the vibrational coordinates and that T_2_ and S_1_ states exhibit many crossing points. This supports the claim that these states are involved in ISC. On the contrary, the crossing between T_1_ and S_1_ states is probably less efficient owing to the substantial energy gap for all but one vibrational coordinate (in the case of **1b** and **1d**).

Based on the theoretical data, we assume that an order of magnitude increase in the quantum yield of ^1^O_2_ production (Φ_Δ_) observed for **1b** and **1d** can be interpreted in terms of increased efficiency of ISC thanks to the vibronic SOC mechanism between S_1_ and T_2_ states. The SOCMEs for **1b** and **1d** show an order of magnitude increase along the vibrational coordinates compared to **1a** and **1c**, which can lead to two orders of magnitude faster population transfer. Therefore, the vibronic SOC can be viewed as a powerful mechanism for electronic population transfer from the optically bright state to the triplet manifold.

## Conclusions

In this work, the singlet oxygen formation efficiency, affinity to singlet oxygen, and photostability of several Cy7 derivatives bearing iodine atom substituents in the C3′ position of the chain and/or the 3*H*-indolium core in methanol and aqueous solutions were determined to evaluate the magnitude of the heavy-atom effect of iodine substitution. The singlet-oxygen production was found to be an order of magnitude more efficient for the chain substitution than the 3*H*-indole core substitution.

The observed dependence cannot be explained solely based on electronic structure calculations for the optimized structures of the synthesized compounds. We show that the variation of SOC along the vibrational coordinates must be considered when interpreting ISC in organic molecules. Although theory showing the fundamentals of the spin–vibronic mechanisms of ISC has been known since the 1960s,^40,71^ only recent experimental and theoretical advances have proved that this mechanism is important in many systems. We demonstrate that a simple qualitative analysis based on the energy gaps or Condon approximations, which assumes that the SOCMEs between states remain unchanged along vibrational motion, cannot provide a complete understanding of the ISC phenomenon. Consequently, we should always be aware of the interplay between spin, electronic, and nuclear dynamics when describing the excited states of molecules, even those that do not contain a heavy element. It should also be emphasized that the standard way of calculating SOCs, i.e., using an effective charge model with effective core potentials for heavy atoms, provides an unsatisfactory description of the system. The evaluation of spin–vibronic coupling should thus become a standard tool for the theoretical analysis of photochemical reactions. This work not only serves for a better understanding of SOC in substituted cyanine dyes but also can help for their further development as photosensitizers, for example, in photodynamic therapy applications.

## Experimental Section

### Materials and Methods

Reagents (2,4-dinitrophenol, *p*-toluenesulfonyl chloride, 3-iodopyridine, 3-methylbutan-2-one, 4-iodophenylhydrazine, 4-hydrazinobenzenesulfonic acid, 2,3,3-trimethylindolenine, 1,3-propane sultone, 4-bromoaniline, methyl iodide, 1,3-diphenylisobenzofuran, rose bengal, and methylene blue) and solvents of the highest purity available were used as purchased, or they were purified/dried using standard methods when necessary. ^1^H NMR spectra were recorded on 300 or 500 MHz spectrometers; ^13^C NMR spectra were obtained on 125 or 75 MHz instruments. ^1^H chemical shifts are reported in parts per million (ppm) relative to *d*_6_-DMSO (δ = 2.50 ppm), CD_3_OD (δ = 3.31 ppm), CDCl_3_ (δ = 7.26 ppm), or D_2_O (δ = 4.79 ppm) as internal references. ^13^C chemical shifts are reported in ppm with *d*_6_-DMSO (δ = 77.67 ppm), CDCl_3_ (δ = 77.16 ppm), and CD_3_OD (δ = 49.30 ppm) as internal references. Absorption spectra and the molar absorption coefficients were obtained on a UV–vis spectrometer with matched 1.0 cm quartz cells. Molar absorption coefficients were determined from the absorption spectra (the average values were obtained from three independent measurements with solutions of different concentrations). Emission and excitation spectra were normalized and smoothed using standard protocols. Flash column chromatography was performed using silica gel (230–400 mesh). The exact masses of the synthesized compounds were obtained using a triple quadrupole electrospray ionization (ESI) mass spectrometer in a positive or negative mode. Melting points were measured on an automatic melting point apparatus. Synthetic procedures were performed under an ambient atmosphere unless stated otherwise. In specified cases, the structural assignment was made with additional information from gCOSY, gHSQC, and gHMBC experiments.

### Fluorescence Measurements

Fluorescence and excitation spectra were measured using a fluorescence spectrometer in 1.0 cm quartz fluorescence cuvettes at 23 ± 1 °C. The sample concentrations were adjusted to keep the absorbance below 0.15 at the corresponding excitation wavelength. Each sample was measured three times**,** and the spectra were averaged.

### Molar Absorption Coefficients

Stock solutions of **1a**–**f** were prepared from the amounts of 3–6 mg in 10 or 25 mL volumetric flasks. The absorbances at different concentrations (10^–7^–10^–5^ M) were measured in 1.0 cm quartz cuvettes. Slopes of the data obtained from the measurements at maximum absorbance wavelengths from three independently prepared solutions were averaged to get molar absorption coefficients using the Beer–Lambert equation *A* = *l c* ε(λ), where *A* is the absorbance, *l* is an optical length, *c* is the concentration, and ε(λ) is a molar absorption coefficient. In some cases (especially for the measurements in PBS), DMSO was added to avoid aggregation.

### Reaction with ^1^O_2_

Rose bengal (for RB: Φ_Δ_ = 0.75,^75,76^ methanol: Φ_Δ_ = 0.76,^77^*c* = 1.0–1.2 × 10^–5^ M) was used as a singlet oxygen sensitizer. Trapping ^1^O_2_ by diphenyl benzofuran (DPBF) in methanol was used as a reference experiment. Solutions (2.5 mL) were stirred in a 1.0 cm quartz cuvette and irradiated with 535 nm LEDs. The absorbance was measured on a UV–vis spectrometer to get 10 experimental points while keeping the conversion smaller than 10% monitored at 410 nm wavelength. The following equation was used for the calculation of the rate constant:^22^
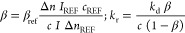
4where Δ*n* is the number of moles of a decomposed compound, Δ*n*_REF_ is the number of moles of a decomposed reference, *k*_d_ is the rate constant of quenching of singlet oxygen by solvent (PBS: *k*_d_ = 2.4 × 10^5^, and methanol: 1.2 × 10^5^ s^–1^),^78^*k*_r_ is the rate of the reaction of DPBF with singlet oxygen (1.3 × 10^9^ M^–1^ s^–1^),^79^ and *I* and *I*_REF_ are the total light absorbed by a sample and a reference, respectively.

### Singlet-Oxygen Production Quantum Yields in Methanol

A solution of 1,3-diphenylisobenzofuran (DPBF; *c* = 5–8 × 10^–5^ M) and one of the **1a**–**f** derivatives (*c* = 4–7 × 10^–6^ M) or methylene blue (MB; *c* = 6–8 × 10^–6^ M) as a standard photosensitizer in methanol was prepared. The stirred solution (3.5 mL) in a 1.0 cm quartz cuvette was irradiated using LEDs at 700 nm, and the UV–vis spectra were recorded periodically. The irradiation time and the decomposition of DPBF were monitored using a UV–vis spectrometer at 411 nm at <10% conversions. All experiments were repeated three times and averaged. The singlet oxygen formation quantum yield was calculated using MB in methanol as a reference (Φ_Δ_ = 0.52)^59,60^ using the following equation:^22^
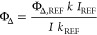
5where Φ_Δ,REF_ is the quantum yield of singlet oxygen production from a reference, *k* is the rate of the consumption of the singlet oxygen trap, and *I* is the amount of absorbed light, where
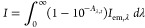
6where *A*_λ,*t*_ is the absorbance of the sample and *I*_em,λ_ is the emission intensity of LEDs.

### Singlet Oxygen Production Quantum Yields in PBS

All measurements and calculations were performed analogous to those in methanol with the exception of the use of 9,10-anthracenediyl-bis(methylene)dimalonic acid (ABDA) (*c* = 1.3 × 10^–4^ M; *k*_r_ = 5.61 × 10^7^ M^–1^ s^–1^)^80^ as a singlet oxygen trap, **1a**–**f** (*c* = 2 × 10^–6^ M), and MB (Φ_Δ_ = 0.52,^81^*c* = 6 × 10^–6^ M) as a reference. The irradiation time and the decomposition of ABDA were monitored on a UV–vis spectrometer at 401 nm at <10% conversions.

### Quantum Yield of Cy7 Decomposition

Solutions of **1a**–**f** at constant concentrations (4–6 × 10^–6^ M) were irradiated in water or methanol under continuous stirring. **1a** (HITC-iodide; Φ_dec_ = 3.1 × 10^–6^)^22^ and **1b** (Φ_dec_ = 84 × 10^–4^)^22^ were used as references in methanol. The concentration changes were measured with a UV–vis spectrometer, and the quantum yield was calculated^22^ using
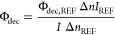
7where *I* is given by
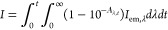
8

### Synthesis

#### 2,4-Dinitrophenyl 4-Methylbenzenesulfonate (**4**)

2,4-Dinitrophenol (10.0 g, 54.3 mmol) was dissolved in dichloromethane (100 mL). Trimethylamine (18.9 mL, 136 mmol) and *p*-toluenesulfonyl chloride (11.4 g, 59.8 mmol) were added in one portion, and the mixture was stirred for 16 h. Afterward, water (100 mL) was added, and the mixture was extracted with dichloromethane (3 × 100 mL), the combined organic extracts were dried with MgSO_4_, and the solvents were evaporated under reduced pressure. The crude product was recrystallized from methanol. Yield: 15.6 g (85%). White solid. Mp. 122.3–125.0 °C. ^1^H NMR (300 MHz, *d*_6_-DMSO): δ (ppm) 8.82 (d, *J* = 2.6 Hz, 1H), 8.58 (dd, *J*_1_ = 2.6 Hz, *J*_2_ = 9.0 Hz, 1H), 7.78 (d, *J* = 8.2 Hz, 2H), 7.59 (d, *J* = 9.0 Hz, 1H), 7.53 (d, *J* = 8.2 Hz, 2H), 2.45 (s, 3H). ^13^C{^1^H} NMR (75 MHz, *d*_6_-DMSO): δ (ppm) 147.2, 145.6, 144.3, 142.5, 130.6, 130.0, 129.6, 128.4, 126.0, 121.8, 21.2. This compound has also been reported elsewhere.^82^

#### 1-(2,4-Dinitrophenyl)pyridin-1-ium *p*-Toluenesulfonate (**5a**)

A mixture of **4** (1.88 g, 5.56 mmol) and pyridine (0.41 mL, 5.05 mmol) in toluene (15 mL) in a pressure tube was heated at 120 °C in an oil bath for 2 h. After cooling to room temperature, toluene (15 mL) was added and the resulting precipitate was filtered off and washed with toluene (3 × 15 mL) and diethyl ether (3 × 15 mL) to give the pure product. Yield: 1.72 g (90%). White solid. Mp. 249.6–254.6 °C. ^1^H NMR (300 MHz, *d_6_*-DMSO): δ (ppm) 9.39 (d, *J* = 6.7 Hz, 2H), 9.11 (d, *J* = 2.5 Hz, 1H), 8.99–8.91 (m, 2H), 8.47–8.40 (m, 3H), 7.45 (d, *J* = 8.0 Hz, 2H), 7.10 (d, *J* = 8.0 Hz, 2H), 2.29 (s, 3H). ^13^C{^1^H} NMR (75 MHz, *d*_6_-DMSO): δ (ppm) 149.0, 148.8, 146.1, 145.7, 143.0, 138.7, 137.5, 131.8, 130.1, 127.97, 127.95, 125.4, 121.4, 20.7. HRMS (ESI-TOF) *m*/*z*: [M]^+^ calcd for C_11_H_8_N_3_O_4_^+^ 246.0510; found 246.0511. This compound has also been reported elsewhere.^26^

#### 1-(2,4-Dinitrophenyl)-3-iodopyridin-1-ium *p*-Toluenesulfonate (**5b**)

3-Iodopyridine (0.75 g, 3.66 mmol) and compound **4** (1.36 g, 4.02 mmol) were added to a pressure tube, and the reaction mixture was heated at 120 °C in an oil bath for 16 h. The precipitate was filtered off and washed with toluene (3 × 15 mL) and diethyl ether (3 × 15 mL) to give the pure product. Yield: 2.12 g (87%). White solid. Mp.: 225.8–228.2 °C. ^1^H NMR (300 MHz, *d_6_*-DMSO): δ (ppm) 9.78 (s, 1H), 9.40 (d, *J* = 6.3 Hz, 1H), 9.30 (d, *J* = 8.2 Hz, 1H), 9.10 (d, *J* = 2.5 Hz, 1H), 8.95 (dd, *J*_1_ = 8.7, *J_2_* = 2.5 Hz, 1H), 8.39 (d, *J* = 8.7 Hz, 1H), 8.20 (dd, *J*_1_ = 8.2, *J_2_* = 6.3 Hz, 1H), 7.45 (d, *J* = 8.0 Hz, 2H), 7.10 (d, *J* = 8.0 Hz, 2H), 2.29 (s, 3H). ^13^C{^1^H} NMR (75 MHz, *d_6_*-DMSO): δ (ppm) 156.1, 150.9, 149.1, 145.6, 145.0, 142.8, 137.9, 137.5, 132.0, 130.1, 128.2, 128.0, 125.4, 121.2, 95.0, 20.7. HRMS (ESI-TOF) *m*/*z*: [M]^+^ calcd for C_11_H_7_IN_3_O_4_^+^ 371.9476; found 371.9479. This compound has also been reported elsewhere.^26^

#### 2,3,3-Trimethyl-5-iodo-3*H*-indole (**2b**)

3-Methyl-butan-2-one (0.8 mL, 7.5 mmol) and sulfuric acid (0.26 mL) were added dropwise to the mixture of 4-iodophenylhydrazine (1.02 g, 4.37 mmol) in ethanol (40 mL), and the reaction mixture was refluxed in an oil bath for 4 h. Afterward, water (100 mL) was added, and the mixture was extracted with dichloromethane (3 × 100 mL), the combined organic extracts were dried with MgSO_4_, and the solvents were evaporated under reduced pressure to give the pure product. Yield: 0.78 g (78%). Orange oil. ^1^H NMR (300 MHz, CDCl_3_): δ (ppm) 7.62 (dd, *J*_1_ = 1.6 Hz, *J*_2_ = 8.0 Hz, 1H), 7.52 (d, *J* = 1.6 Hz, 1H), 7.30 (d, *J* = 8.0 Hz, 1H), 2.25 (s, 3H), 1.29 (s, 6H). ^13^C{^1^H} NMR (75 MHz, CDCl_3_): δ (ppm) 188.5, 153.1, 148.1, 136.7, 130.7, 121.8, 90.0, 54.0, 22.9, 15.2. HRMS (ESI-TOF) *m*/*z*: [M + H]^+^ calcd for C_11_H_13_IN^+^ 286.0088; found 286.0089. This compound has also been reported elsewhere.^83^

#### Sodium 2,3,3-Trimethylindole-5-sulfonate (**2c**)

4-Hydrazino-benzenesulfonic acid (14.4 g, 76.6 mmol) and 3-methyl-butan-2-one (16.4 mL, 153 mmol) were dissolved in acetic acid (30 mL), and the mixture was refluxed in an oil bath for 3 h. After cooling down to 22 °C, ethyl acetate (100 mL) was added and the resulting pink precipitate was filtered and washed with ethyl acetate (3 × 50 mL). Residual acetic acid was evaporated under reduced pressure. The resulting compound was dissolved in a mixture of NaOH (4.59 g, 115 mmol) in methanol/isopropanol (50/50 mL) and refluxed for 30 min. Afterward, methanol was evaporated under reduced pressure and the resulting precipitate was filtered and washed with isopropanol (1 × 50 mL) and diethyl ether (3× 50 mL) to give the pure product. Yield: 17.5 g (85%). Brown solid. Mp.: 239.0–247.8 °C. ^1^H NMR (300 MHz, *d_6_*-DMSO): δ (ppm) 7.63–7.61 (m, 1H), 7.54 (dd, *J*_1_ = 1.7 Hz, *J*_2_ = 7.9 Hz, 1H), 7.33 (d, *J* = 7.9 Hz, 1H), 2.21 (s, 3H), 1.24 (s, 6H). ^13^C{^1^H} NMR (75 MHz, *d_6_*-DMSO): δ (ppm) 188.9, 153.7, 145.2, 145.0, 125.1, 119.2, 118.2, 53.3, 22.5, 15.2. HRMS (ESI-TOF) *m*/*z*: [M]^−^ calcd for C_11_H_12_NO_3_S^–^ 238.0543; found 238.0545. This compound has also been reported elsewhere.^84^

#### 1,2,3,3-Tetramethyl-3*H*-indolium Iodide (**3a**)

A mixture of 2,3,3-trimethylindolenine (5.00 g, 31.4 mmol) and methyl iodide (8.91 g, 62.8 mmol) in toluene (50 mL) was heated at 100 °C in an oil bath for 2 h. After cooling to room temperature, the precipitate was filtered and washed with toluene (3 × 15 mL) and diethyl ether (3 × 15 mL) to give the pure product. Yield: 8.8 g (93%). Red solid. Mp.: 237.6 °C (decomp.). ^1^H NMR (300 MHz, *d*_6_-DMSO): δ (ppm) 7.95–7.88 (m, 1H), 7.85–7.79 (m, 1H), 7.67–7.57 (m, 2H), 3.98 (s, 3H), 2.77 (s, 3H), 1.53 (s, 6H). ^13^C{^1^H} NMR (75 MHz, *d*_6_-DMSO): δ (ppm) 195.9, 142.0, 141.5, 129.2, 128.7, 123.2, 115.1, 53.9, 34.8, 21.7, 14.3. HRMS (ESI-TOF) *m*/*z*: [M]^+^ calcd for C_12_H_16_N^+^ 174.1277; Found 174.1274. This compound has also been reported elsewhere.^26^

#### 5-Iodo-1,2,3,3-tetramethyl-3*H*-indol-1-ium Iodide (**3b**)

A mixture of **2b** (1.05 g, 3.68 mmol) and methyl iodide (0.98 mL, 7.37 mmol) in toluene (10 mL) in a pressure tube was heated at 120 °C in an oil bath for 1.5 h. After cooling down to room temperature, the resulting precipitate was filtered off and washed with toluene (3 × 20 mL) and diethyl ether (3 × 20 mL) to give the pure product. Yield: 1.3 g (85%). Brown solid. Mp.: 232.8–237.6 °C. ^1^H NMR (300 MHz, *d*_6_-DMSO): δ (ppm) 8.28 (d, *J* = 1.1 Hz, 1H), 8.00 (dd, *J*_1_ = 8.4, *J_2_* = 1.1 Hz, 1H), 7.72 (d, *J* = 8.4 Hz, 1H), 3.94 (s, 3H), 2.73 (s, 3H), 1.52 (s, 6H). ^13^C{^1^H} NMR (75 MHz, *d*_6_-DMSO): δ (ppm) 196.0, 143.7, 141.9, 137.4, 132.1, 117.1, 96.0, 54.0, 34.8, 21.4, 14.2. HRMS (ESI-TOF) *m*/*z*: [M]^+^ calcd for C_12_H_15_IN^+^ 300.0244; found 300.0241. This compound has also been reported elsewhere.^85^

#### Sodium 1-(2-Sulfonatopropyl)-2,3,3-trimethyl-3*H*-indolenine-5-sulfonate (**3c**)

**2c** (2.13 g, 8.15 mmol) and 1,3-propane sultone (1.49 g, 12.2 mmol) were dissolved in toluene (15 mL) and refluxed in an oil bath for 18 h. After cooling, toluene was evaporated under reduced pressure, the resulting compound was dissolved in water (10 mL) and precipitated with acetone (30 mL), and the precipitate was filtered off and washed with diethyl ether (3 × 20 mL). The precipitation was repeated twice to give the pure product. Yield: 0.99 g (32%). Pink solid. Mp.: 265.6–270.1 °C. ^1^H: NMR (500 MHz, *d*_6_-DMSO): δ (ppm) 8.01 (s, 1H), 7.98 (d, *J* = 8.2 Hz, 1H), 7.82 (d, *J* = 8.2 Hz, 1H), 4.64 (t, *J* = 7.3 Hz, 2H), 2.84 (s, 3H), 2.63 (t, *J* = 5.9 Hz, 2H), 2.21–2.12 (m, 2H), 1.54 (s, 6H). ^13^C{^1^H} NMR (125 MHz, *d*_6_-DMSO): δ (ppm) 197.3, 159.6, 141.6, 141.0, 126.3, 120.7, 114.8, 54.2, 47.3, 46.7, 23.7, 21.9, 13.9. HRMS (ESI-TOF) *m*/*z*: [M]^−^ calcd for C_14_H_18_NO_6_S_2_^–^ 360.0581; found 360.0584. This compound has also been reported elsewhere.^86^

#### 2,3,3-Trimethyl-1-[3-(trimethylammonium)propyl]-3*H*-indolinium-5-sulfonate Bromide (**3d**)

**2c** (1.87 g, 7.15 mmol), 3-bromo-*N*,*N*,*N*-trimethyl-1-propanaminium bromide (3.73 g, 14.31 mmol), and sodium iodide (190 mg, 1,27 mmol) were dissolved in acetonitrile (20 mL) in a pressure tube and heated at 120 °C in an oil bath for 48 h. After cooling, the resulting precipitate was filtered off and washed with isopropanol (3 × 15 mL) and diethyl ether (2 × 40 mL) to give the pure product. Yield: 2.79 g (93%). Purple solid. Mp.: 219.7–224.7 °C, ^1^H NMR (300 MHz, CD_3_OD): δ (ppm) 8.15–8.09 (m, 2H), 7.88 (d, *J* = 8.3, 1H), 4.65 (t, *J* = 8.0, 2H), 3.87–3.78 (m, 2H), 3.28 (s, 9H), 2.61–2.48 (m, 2H), 1.67 (s, 6H). ^13^C{^1^H} NMR (75 MHz, CD_3_OD): δ (ppm) 201.5, 148.8, 143.5, 143.3, 128.4, 122.4, 116.6, 64.0, 56.5, 54.1, 46.1, 30.3, 22.9, 22.7. HRMS (ESI-TOF) *m*/*z*: [M]^+^ calcd for C_17_H_27_N_2_O_3_S^+^ 339.1742; found 339.1738. This compound has also been reported elsewhere.^84^

#### 2-((1*E*,3*Z*,5*E*)-7-((*E*)-1,3,3-Trimethylindolin-2-ylidene)hepta-1,3,5-trien-1-yl)-1,3,3-trimethyl-3*H*-indol-1-ium Iodide (**1a**)

**5a** (200 mg, 0.48 mmol) and 4-bromoaniline (247 mg, 1.44 mmol) were dissolved in methanol (5 mL), and the mixture was stirred at room temperature for 30 min. Then, **3a** (433 mg, 1.44 mmol) and sodium acetate (236 mg, 2.88 mmol) were added and the reaction mixture was stirred at room temperature for additional 6 h. Then, diethyl ether (7.5 mL) was added and the product was stored in the fridge (−5 °C) for 16 h. The resulting precipitate was filtered off and washed with water (3 × 15 mL) and diethyl ether (3 × 15 mL). The crude product was purified by flash chromatography (dichloromethane/methanol, 30: 1). Yield: 195 mg (76%). Green solid. Mp.: 173.4 °C (decomp.). ^1^H NMR (500 MHz, CD_3_OD): δ (ppm) 7.93 (dd, *J*_1_ = 13.7, *J_2_* = 12.7 Hz, 2H), 7.62 (t, *J* = 12.6 Hz, 1H), 7.46 (d, *J* = 7.4 Hz, 2H), 7.44–7.36 (m, 2H), 7.29–7.20 (m, 4H), 6.56 (dd, *J*_1_ = *J*_2_ = 12.7 Hz, 2H), 6.26 (d, *J* = 13.7 Hz, 2H), 3.60 (s, 6H), 1.69 (s, 12H). ^13^C{^1^H} NMR (125 MHz, CD_3_OD): δ (ppm) 173.9, 157.6, 152.9, 144.4, 142.4, 129.7, 126.9, 126.0, 123.2, 111.6, 104.8, 50.3, 31.5, 27.9. HRMS (ESI-TOF) *m*/*z*: [M]^+^ calcd for C_29_H_33_N_2_^+^ 409.2638; found 409.2640. This compound has also been reported elsewhere.^26^

#### 2-((1*E*,3*Z*,5*E*)-3-Iodo-7-((*E*)-1,3,3-trimethylindolin-2-ylidene)hepta-1,3,5-trien-1-yl)-1,3,3-trimethyl-3*H*-indol-1-ium Iodide (**1b**)

**5b** (200 mg, 0.37 mmol) and 4-bromoaniline (190 mg, 1.1 mmol) were dissolved in methanol (5 mL), and the reaction mixture was stirred at room temperature for 30 min. Then, **3a** (330 mg, 1.1 mmol) and sodium acetate (180 mg, 2.2 mmol) were added, and the mixture was stirred at room temperature for additional 16 h. Afterward, diethyl ether (15 mL) was added and the product was stored in the fridge (−5 °C) for 16 h. The precipitate was filtered and washed with water (3 × 10 mL) and diethyl ether (2 × 10 mL). The crude product was purified on a flash chromatography column (dichloromethane/methanol, 30: 1). Yield: 180 mg (75%). Green solid. Mp.: 201.1–205.5 °C. ^1^H NMR (300 MHz, CD_3_OD): δ (ppm) 8.19 (dd, *J*_1_ = 14.0, *J*_2_ = 12.2 Hz, 1H), 7.75 (d, *J* = 12.9 Hz, 1H), 7.61 (d, *J* = 12.4 Hz, 1H), 7.55 (d, *J* = 7.5 Hz, 1H), 7.49–7.42 (m, 3H), 7.40–7.33 (m, 2H), 7.29–7.19 (m, 2H), 6.80 (dd, *J*_1_ = 12.4 Hz, *J*_2_ = 12.2 Hz, 1H), 6.59 (d, *J* = 14.0 Hz, 1H), 6.21 (d, *J* = 12.9 Hz, 1H), 3.74 (s, 3H), 3.60 (s, 3H), 1.73 (s, 6H), 1.69 (s, 6H). ^13^C{^1^H} NMR (125 MHz, CD_3_OD): δ (ppm) 177.3, 173.8, 158.1, 155.2, 151.9, 144.4, 144.0, 143.1, 142.1, 130.0, 129.7, 129.6, 127.5, 125.8, 123.4, 123.3, 112.9, 111.4, 107.8, 106.0, 95.4, 51.3, 50.2, 32.2, 31.4, 27.9, 27.6. HRMS (ESI-TOF) *m*/*z*: [M]^+^ calcd for C_29_H_32_IN_2_^+^ 535.1605; found 535.1601. This compound has also been reported elsewhere.^26^

#### 2-((1*E*,3*Z*,5*E*)-7-((*E*)-5-Iodo-1,3,3-trimethylindolin-2-ylidene)hepta-1,3,5-trien-1-yl)-5-iodo-1,3,3-trimethyl-3*H*-indol-1-ium Iodide (**1c**)

**5a** (100 g, 0.24 mmol) and 4-bromoaniline (124 mg, 0.719 mmol) were dissolved in methanol (2.5 mL), and the mixture was stirred at room temperature for 30 min. Then, **3b** (310 mg, 0.72 mmol) and sodium acetate (118 mg, 1.44 mmol) were added and the reaction mixture was stirred at 45 °C in an oil bath for additional 6 h. Afterward, diethyl ether (7.5 mL) was added, and the product was stored in the fridge (−5 °C) for 16 h. The resulting precipitate was filtered off and washed with water (3 × 10 mL) and diethyl ether (3 × 10 mL). The crude product was purified using flash chromatography (dichloromethane/methanol, 30: 1). Yield: 49 mg (28%). Green solid. Mp.: 220.1–230.7 °C. ^1^H NMR (500 MHz, *d*_6_-DMSO): δ (ppm) 7.95 (d, *J* = 1.5 Hz, 2H), 7.86 (dd, *J*_1_ = 13.8, *J*_2_**=** 12.6 Hz, 2H), 7.76 (dd, *J*_1_ = *J*_2_ = 12.6 Hz, 1H), 7.73 (dd, *J*_1_ = 8.3, *J*_2_ = 1.6 Hz, 2H), 7.19 (d, *J* = 8.4 Hz, 2H), 6.54 (dd, *J*_1_*= J*_2_ = 12.6 Hz, 2H), 6.30 (d, *J* = 13.8 Hz, 2H), 3.54 (s, 6H), 1.62 (s, 12H). ^13^C{^1^H} NMR (125 MHz, *d*_6_-DMSO): δ (ppm) 171.0, 156.1, 150.8, 143.3, 142.8, 136.9, 130.9, 125.8, 113.1, 104.1, 88.6, 48.6, 31.1, 26.8. HRMS (ESI-TOF) *m*/*z*: [M]^+^ calcd for C_29_H_31_I_2_N_2_^+^ 661.0571; found 661.0573.

#### 2-((1*E*,3*Z*,5*E*)-3-Iodo-7-((*E*)-5-iodo-1,3,3-trimethylindolin-2-ylidene)hepta-1,3,5-trien-1-yl)-5-iodo-1,3,3-trimethyl-3*H*-indol-1-ium Iodide (**1d**)

**5b** (150 mg, 0.28 mmol) and 4-bromoaniline (83 mg, 0.14 mmol) were dissolved in methanol (2.8 mL), and the mixture was stirred at room temperature for 30 min. Then, **3b** (350 g, 0.83 mmol) and sodium acetate (140 mg, 1.7 mmol) were added and the reaction mixture was stirred at 45 °C in an oil bath for additional 6 h. Diethyl ether (8.4 mL) was added, and the product was stored in the fridge (−5 °C) for 16 h. The resulting precipitate was filtered off and washed with water (3 × 10 mL) and diethyl ether (3 × 10 mL). The crude product was purified by flash chromatography (dichloromethane/methanol, 30: 1). Yield: 84 mg (33%). Green solid. Mp.: 148.7–150.4 °C. ^1^H NMR (500 MHz, *d*_6_-DMSO): δ (ppm) 8.09 (dd, *J*_1_ = 12.2, *J*_2_ = 14.2 Hz, 1H), 8.08 (d, *J* = 1.5 Hz, 1H), 7.96 (d, *J* = 1.5 Hz, 1H), 7.90 (d, *J* = 12.3 Hz, 1H), 7.82 (dd, *J*_1_ = 8.4, *J*_2_ = 1.6 Hz, 1H), 7.74 (d, *J* = 12.9 Hz, 1H), 7.71 (dd, *J*_1_ = 8.4, *J*_2_ = 1.6 Hz, 1H), 7.35 (d, *J* = 8.4 Hz, 1H), 7.19 (d, *J* = 8.4 Hz, 1H), 6.72 (d, *J* = 14.2 Hz, 1H), 6.67 (dd, *J*_1_ = 12.2 Hz, *J*_2_ = 12.3 Hz, 1H), 6.01 (d, *J* = 12.9 Hz, 1H), 3.70 (s, 3H), 3.53 (s, 3H), 1.66 (s, 6H), 1.63 (s, 6H). ^13^C{^1^H} NMR (125 MHz, *d*_6_-DMSO): δ (ppm) 174.8, 170.7, 158.2, 153.3, 149.7, 144.0, 143.0, 142.8, 142.3, 137.1, 136.8, 131.2, 131.0, 128.2, 114.4, 112.8, 107.6, 104.3, 95.6, 90.9, 88.1, 49.6, 48.5, 32.0, 30.9, 26.8, 26.4. HRMS (ESI-TOF) *m*/*z*: [M]^+^ calcd for C_29_H_30_I_3_N_2_^+^ 786.9538; found 786.9542.

#### Sodium 3-[2-[7-[3,3-Dimethyl-5-sulfo-1-(3-(*N*-(3-sulfonatopropyl)))-3*H*-indol-1-ium-2-yl]-3-iodo-2,4,6-heptatrien-1-ylidene]-3,3-dimethyl-5-sulfo-1,2-dihydro-3*H*-indol-1-yl]-propanesulfonate (**1e**)

**5b** (200 mg, 0.37 mmol) and 4-aminobenzonitrile (190 mg, 1.1 mmol) were dissolved in methanol (5.5 mL), and the mixture was stirred for 30 min at room temperature. Then, **3d** (420 mg, 1.1 mmol) and sodium acetate (180 mg, 2.2 mmol) were added and the reaction mixture was stirred for an additional 16 h at 45 °C in an oil bath. The reaction mixture was cooled down to room temperature, and the resulting precipitate was filtered off and washed with ethanol (2 × 5 mL), acetone (5 × 10 mL), and diethyl ether (3 × 5 mL) to give the pure product. Yield: 310 g (87%). Green solid. Mp.: 353.3 °C (decomp.). ^1^H NMR (500 MHz, D_2_O): δ (ppm) 8.03 (dd, *J*_1_ = 13.9 Hz, *J*_2_ = 12.3 Hz, 1H), 7.93 (d, *J* = 1.5 Hz, 1H), 7.89–7.83 (m, 2H), 7.78 (dd, *J*_1_ = 8.3, *J*_2_ = 1.5 Hz, 1H), 7.65 (d, *J* = 12.7 Hz, 1H), 7.49 (d, *J* = 8.3 Hz, 1H), 7.47–7.38 (m, 1H), 6.73 (dd, *J*_1_ = *J*_2_ = 12.3 Hz, 1H), 6.51 (d, *J* = 13.9 Hz, 1H), 6.26 (d, *J* = 12.7 Hz, 1H), 4.54–4.26 (m, 2H), 3.33–2.96 (m, 4H), 2.45–2.24 (m, 4H), 1.68 (s, 6H), 1.66 (s, 6H). ^13^C{^1^H} NMR (125 MHz, *d*_6_-DMSO) δ (ppm) 175.4, 170.8, 156.9, 153.6, 149.8, 146.6, 144.8142.4, 141.6, 141.3, 139.8, 128.1, 126.2, 126.0, 119.8, 119.7, 111.5, 109.7, 107.4, 104.3, 95.5, 49.7, 48.5, 48.3, 47.7, 43.6, 43.0, 27.1, 26.8, 23.8, 23.1. HRMS (ESI-TOF) *m*/*z*: [M]^3–^ calcd for C_33_H_36_IN_2_O_12_S_4_^3–^ 302.3404; found 302.3408.

#### 3-[2-[7-[3,3-Dimethyl-5-sulfo-1-(3-(trimethylammonium)propyl)-3*H*-indol-1-ium-2-yl]-3-iodo-2,4,6-heptatrien-1-ylidene]-3,3-dimethyl-5-sulfo-1,2-dihydro-3*H*-indol-1-yl]-propanesulfonate Bromide (**1f**)

**5b** (150 mg, 0.28 mmol) and 4-aminobenzonitrile (98 mg, 0.82 mmol) were dissolved in methanol (2.8 mL), and the mixture was stirred at room temperature for 30 min. Then, **3c** (350 mg, 0.82 mmol) and sodium acetate (260 mg, 1.66 mmol) were added and the mixture was stirred at 45 °C in an oil bath for an additional 6 h. The reaction mixture was cooled down to room temperature, and the resulting precipitate was filtered off and washed with ethanol (2 × 5 mL), acetone (5 × 10 mL), and diethyl ether (3 × 5 mL). The compound was dissolved in water (10 mL), acetone (30 mL) was added dropwise, and the solution was stored in the fridge (−5 °C) for 6 h. The resulting precipitate was filtered off and washed with acetone (5 × 10 mL) and diethyl ether (3 × 5 mL) to give the pure product. Yield: 140 mg (54%). Green solid. Mp.: 268.1 °C (decomp.). ^1^H NMR (500 MHz, *d*_6_-DMSO): δ (ppm) 8.19 (dd, *J*_1_ = 14.1, *J*_2_ = 12.3 Hz, 1H), 8.00 (d, *J* = 12.7 Hz, 1H), 7.90 (d, *J* = 1.4 Hz, 1H), 7.84–7.80 (m, 2H), 7.73 (dd, *J*_1_ = 8.3, *J*_2_ = 1.4 Hz, 1H), 7.66 (dd, *J*_1_ = 8.3, *J*_2_ = 1.5 Hz, 1H), 7.55 (d, *J* = 8.3 Hz, 1H), 7.35 (d, *J* = 8.3 Hz, 1H), 6.78 (d, *J* = 14.1 Hz, 1H), 6.69 (dd, *J*_1_ = *J*_2_ = 12.3 Hz, 1H), 6.09 (d, *J* = 12.7 Hz, 1H), 4.28 (t, *J* = 7.4 Hz, 2H), 4.14 (t, *J* = 7.2 Hz, 2H), 3.58–3.41 (m, 4H), 3.11 (s, 9H), 3.10 (s, 9H), 2.27–2.10 (m, 4H), 1.72 (s, 6H), 1.68 (s, 6H). ^13^C{^1^H} NMR (125 MHz, *d*_6_-DMSO): δ (ppm) 175.6, 171.2, 157.7, 154.1, 150.3, 147.0, 145.4, 141.8, 141.22, 141.19, 139.8, 128.2, 126.3, 126.1, 120.05, 120.01, 111.4, 109.6, 107.3, 104.5, 95.8, 62.7, 62.4, 52.6, 52.4, 49.8, 48.7, 41.5, 40.9, 27.1, 26.8, 21.1, 20.4. HRMS (ESI-TOF) *m*/*z*: [M]^+^ calcd for C_39_H_54_IN_4_O_6_S_2_^+^ 865.2524; found 865.2517.

#### Ab Initio Calculations—Technical Details

The structures of Cy7 derivatives were optimized in water (used as an archetype of a polar solvent) represented by a dielectric continuum, using the polarizable continuum model (PCM).^87^ The electronic structure calculations were performed at the density functional level, using the CAM-B3LYP functional and the def2-TZVP and def2-SVP basis sets for optimization; the structures are shown in Supporting Information. The choice of the functional was motivated by our previous investigation of analogous cyanine molecules^22^ and by the fact that the range-separate hybrid functionals have recently been proven to provide an accurate description of the conjugated double bonds.^88^ However, the excitation energies of cyanines are generally relatively insensitive to the choice of functional.^37^ Vibrational frequencies, SOC matrix elements (SOCMEs), and the TDDFT excitation energies—with and without a TDA^89^—were calculated at the same levels of theory. To calculate the SOCMEs, we used the Breit–Pauli SOC operator with a mean-field approximation with exact two-electron terms as implemented in the ORCA 5.0.1 package.^90−92^ To include the scalar relativistic effects, we used the full Douglas–Kroll Hamiltonian,^93^ which was a critical step to obtain reliable values of the SOCs. The Hessian with the relativistic Douglas–Kroll Hamiltonian (DKH) and PCM for solvent is available only for the ground electronic state. We assumed that the normal modes of *q_i_* are the same for the ground state and the S_1_ state because the optimized geometries are very similar. The basis set dependence of SOCMEs is not critical. The tests were performed for **1b** and are summarized in Table S6. This approach for the calculations of SOCMEs was shown to lead to errors of ∼5%.^94^

The SOCME scans were performed as a function of dimensionless normal coordinates δ*q_i_*; the particular molecular distortions were constructed to correspond to variation of the normal coordinates from −*l_i_*δ*q_i_* to +*r_i_*δ*q_i_*, where *l_i_* and *r_i_* specify the number of steps in positive and negative directions along each normal mode. The relation between the dimensionless normal coordinate and the vector of atomic Cartesian displacements δ***X*** is defined in the ORCA code as
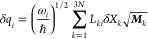
where *L_ki_* is the orthogonal matrix obtained upon numerical diagonalization of the mass-weighted Hessian matrix and ***M*** is the vector of atomic masses. The Hessian matrix was obtained for optimized geometries of the ground electronic state at the same level of theory. Note that the optimization of excited-state geometries within the same level of theory with PCM is not implemented yet. The optimization of the S_1_ excited-state geometries was performed in a vacuum at the CAM-B3LYP/def2-SVP level, and the geometries were compared to those of the ground state structure at the same level of theory. These geometries were used for calculations of the SOCMEs and TDDFT energies at the CAM-B3LYP/def2-TZVP levels at the S_1_ minimum. All electronic structure calculations were performed in ORCA, version 5.0.1.^90−92^

## Data Availability

The data underlying this study are available in the published article and its online supporting material.

## Supporting Information Available

Absorption and emission spectra; NMR spectra; quantum-chemical calculations; Cartesian coordinates; excitation energies; spin–orbit matrix elements; analyses of photoproducts (PDF)

## References

[ref1] Huang J.; Pu K. Activatable Molecular Probes for Second Near-Infrared Fluorescence, Chemiluminescence, and Photoacoustic Imaging. Angew. Chem., Int. Ed. 2020, 59, 11717 10.1002/anie.202001783. 32134156

[ref2] Zhang Y.; Bi J.; Xia S.; Mazi W.; Wan S.; Mikesell L.; Luck R. L.; Liu H. A near-infrared fluorescent probe based on a FRET rhodamine donor linked to a cyanine acceptor for sensitive detection of intracellular pH alternations. Molecules 2018, 23, 2679 10.3390/molecules23102679. 30340334 PMC6222743

[ref3] Li S.; Zhang D.; Xie X.; Ma S.; Liu Y.; Xu Z.; Gao Y.; Ye Y. A novel solvent-dependently bifunctional NIR absorptive and fluorescent ratiometric probe for detecting Fe3+/Cu2+ and its application in bioimaging. Sens. Actuators, B 2016, 224, 661 10.1016/j.snb.2015.10.086.

[ref4] Gao X.; Wu W.; Xi J.; Zheng H. Manipulation of monomer-aggregate transformation of a heptamethine cyanine ligand: near infrared chromogenic recognition of Hg^2+^. RSC Adv. 2017, 7, 32732 10.1039/C7RA03517A.

[ref5] Liu Y.; Chen M.; Cao T.; Sun Y.; Li C.; Liu Q.; Yang T.; Yao L.; Feng W.; Li F. A cyanine-modified nanosystem for in vivo upconversion luminescence bioimaging of methylmercury. J. Am. Chem. Soc. 2013, 135, 9869 10.1021/ja403798m. 23763640

[ref6] Shealy D. B.; Lipowska M.; Lipowski J.; Narayanan N.; Sutter S.; Strekowski L.; Patonay G. Synthesis, chromatographic separation, and characterization of near-infrared labeled DNA oligomers for use in DNA sequencing. Anal. Chem. 1995, 67, 247 10.1021/ac00098a002.

[ref7] Vus K.; Tarabara U.; Kurutos A.; Ryzhova O.; Gorbenko G.; Trusova V.; Gadjev N.; Deligeorgiev T. Aggregation behavior of novel heptamethine cyanine dyes upon their binding to native and fibrillar lysozyme. Mol. BioSyst. 2017, 13, 970 10.1039/C7MB00185A. 28379242

[ref8] Shen Z.; Prasai B.; Nakamura Y.; Kobayashi H.; Jackson M. S.; McCarley R. L. A near-infrared, wavelength-shiftable, turn-on fluorescent probe for the detection and imaging of cancer tumor cells. ACS Chem. Biol. 2017, 12, 1121 10.1021/acschembio.6b01094. 28240865 PMC7900918

[ref9] Nani R. R.; Gorka A. P.; Nagaya T.; Kobayashi H.; Schnermann M. J. Near-IR light-mediated cleavage of antibody–drug conjugates using cyanine photocages. Am. Ethnol. 2015, 127, 13839 10.1002/ange.201507391. PMC4743669 26403799

[ref10] Stackova L.; Russo M.; Muchova L.; Orel V.; Vítek L.; Stacko P.; Klan P. Cyanine-Flavonol Hybrids for Near-Infrared Light-Activated Delivery of Carbon Monoxide. Chem. – Eur. J. 2020, 26, 13184 10.1002/chem.202003272. 32885885 PMC7693251

[ref11] Food U.; Administration D. In Approved drug products with therapeutic equivalence evaluations; US Food and Drug Administration (FDA): 2021.

[ref12] Ogasawara Y.; Ikeda H.; Takahashi M.; Kawasaki K.; Doihara H. Evaluation of breast lymphatic pathways with indocyanine green fluorescence imaging in patients with breast cancer. World J. Surg. 2008, 32, 1924 10.1007/s00268-008-9519-7. 18330628

[ref13] Cao J.; Chi J.; Xia J.; Zhang Y.; Han S.; Sun Y. Iodinated cyanine dyes for fast near-infrared-guided deep tissue synergistic phototherapy. ACS Appl. Mater. Interfaces 2019, 11, 25720 10.1021/acsami.9b07694. 31246000

[ref14] Liu H.; Yin J.; Xing E.; Du Y.; Su Y.; Feng Y.; Meng S. Halogenated cyanine dyes for synergistic photodynamic and photothermal therapy. Dyes Pigm. 2021, 190, 109327 10.1016/j.dyepig.2021.109327.

[ref15] Atchison J.; Kamila S.; Nesbitt H.; Logan K. A.; Nicholas D. M.; Fowley C.; Davis J.; Callan B.; McHale A. P.; Callan J. F. Iodinated cyanine dyes: a new class of sensitisers for use in NIR activated photodynamic therapy (PDT). Chem. Commun. 2017, 53, 2009 10.1039/C6CC09624G. 28124050

[ref16] Klan P.; Wirz J. Photochemistry of Organic Compounds: From Concepts to Practice; 1^st^ ed.; John Wiley & Sons Ltd.: Chichester, 2009.

[ref17] Solov’ev K. N.; Borisevich E. A. Intramolecular heavy-atom effect in the photophysics of organic molecules. Phys.-Usp. 2005, 48, 231 10.1070/PU2005v048n03ABEH001761.

[ref18] Yang X.; Bai J.; Qian Y. The investigation of unique water-soluble heptamethine cyanine dye for use as NIR photosensitizer in photodynamic therapy of cancer cells. Spectrochim. Acta, Part A 2020, 228, 117702 10.1016/j.saa.2019.117702. 31748160

[ref19] Li M.; Sun W.; Tian R.; Cao J.; Tian Y.; Gurram B.; Fan J.; Peng X. Smart J-aggregate of cyanine photosensitizer with the ability to target tumor and enhance photodynamic therapy efficacy. Biomaterials 2021, 269, 120532 10.1016/j.biomaterials.2020.120532. 33228992

[ref20] Zhao X.; Yao Q.; Long S.; Chi W.; Yang Y.; Tan D.; Liu X.; Huang H.; Sun W.; Du J. An Approach to Developing Cyanines with Simultaneous Intersystem Crossing Enhancement and Excited-State Lifetime Elongation for Photodynamic Antitumor Metastasis. J. Am. Chem. Soc. 2021, 143, 12345 10.1021/jacs.1c06275. 34323480

[ref21] Jarman J. B.; Dougherty D. A. Charge-transfer heptamethine dyes for NIR singlet oxygen generation. Chem. Commun. 2019, 55, 5511 10.1039/C9CC01096C. 31020279

[ref22] Stackova L.; Muchova E.; Russo M.; Slavicek P.; Stacko P.; Klan P. Deciphering the Structure–Property Relations in Substituted Heptamethine Cyanines. J. Org. Chem. 2020, 85, 9776 10.1021/acs.joc.0c01104. 32697591

[ref23] Montagnon T.; Tofi M.; Vassilikogiannakis G. Using singlet oxygen to synthesize polyoxygenated natural products from furans. Acc. Chem. Res. 2008, 41, 1001 10.1021/ar800023v. 18605738

[ref24] Mathon B.; Choubert J.-M.; Miege C.; Coquery M. A review of the photodegradability and transformation products of 13 pharmaceuticals and pesticides relevant to sewage polishing treatment. Sci. Total Environ. 2016, 551, 712 10.1016/j.scitotenv.2016.02.009. 26907739

[ref25] Gorman A.; Killoran J.; O’Shea C.; Kenna T.; Gallagher W. M.; O’Shea D. F. In vitro demonstration of the heavy-atom effect for photodynamic therapy. J. Am. Chem. Soc. 2004, 126, 10619 10.1021/ja047649e. 15327320

[ref26] Stackova L.; Stacko P.; Klan P. Approach to a substituted heptamethine cyanine chain by the ring opening of Zincke salts. J. Am. Chem. Soc. 2019, 141, 7155 10.1021/jacs.9b02537. 31017409

[ref27] Autschbach J. Why the Particle-in-a-Box Model Works Well for Cyanine Dyes but Not for Conjugated Polyenes. J. Chem. Educ. 2007, 84, 1840 10.1021/ed084p1840.

[ref28] Champagne B.; Guillaume M.; Zutterman F. TDDFT investigation of the optical properties of cyanine dyes. Chem. Phys. Lett. 2006, 425, 105 10.1016/j.cplett.2006.05.009.

[ref29] Grimme S.; Neese F. Double-hybrid density functional theory for excited electronic states of molecules. J. Chem. Phys. 2007, 127, 154116 10.1063/1.2772854. 17949141

[ref30] Jacquemin D.; Wathelet V.; Perpète E. A.; Adamo C. Extensive TD-DFT Benchmark: Singlet-Excited States of Organic Molecules. J. Chem. Theory Comput. 2009, 5, 2420 10.1021/ct900298e. 26616623

[ref31] Fabian J. TDDFT-calculations of Vis/NIR absorbing compounds. Dyes Pigm. 2010, 84, 36 10.1016/j.dyepig.2009.06.008.

[ref32] Jacquemin D.; Zhao Y.; Valero R.; Adamo C.; Ciofini I.; Truhlar D. G. Verdict: time-dependent density functional theory “not guilty” of large errors for cyanines. J. Chem. Theory Comput. 2012, 8, 1255 10.1021/ct200721d. 26596742

[ref33] Schreiber M.; Buß V.; Fülscher M. P. The electronic spectra of symmetric cyanine dyes: A CASPT2 study. Phys. Chem. Chem. Phys. 2001, 3, 3906 10.1039/b103417k.

[ref34] Jacquemin D.; Perpète E. A.; Scalmani G.; Frisch M. J.; Kobayashi R.; Adamo C. Assessment of the efficiency of long-range corrected functionals for some properties of large compounds. J. Chem. Phys. 2007, 126, 144105 10.1063/1.2715573. 17444699

[ref35] Send R.; Valsson O.; Filippi C. Electronic Excitations of Simple Cyanine Dyes: Reconciling Density Functional and Wave Function Methods. J. Chem. Theory Comput. 2011, 7, 444 10.1021/ct1006295. 26596164

[ref36] Masunov A. E. Theoretical spectroscopy of carbocyanine dyes made accurate by frozen density correction to excitation energies obtained by TD-DFT. Int. J. Quantum Chem. 2010, 110, 3095 10.1002/qua.22923.

[ref37] Le Guennic B.; Jacquemin D. Taking up the cyanine challenge with quantum tools. Acc. Chem. Res. 2015, 48, 530 10.1021/ar500447q. 25710687 PMC4365665

[ref38] Moore B.; Autschbach J. Longest-wavelength electronic excitations of linear cyanines: the role of electron delocalization and of approximations in time-dependent density functional theory. J. Chem. Theory Comput. 2013, 9, 4991 10.1021/ct400649r. 26583416

[ref39] Zhekova H.; Krykunov M.; Autschbach J.; Ziegler T. Applications of Time Dependent and Time Independent Density Functional Theory to the First π to π* Transition in Cyanine Dyes. J. Chem. Theory Comput. 2014, 10, 3299 10.1021/ct500292c. 26588299

[ref40] Penfold T. J.; Gindensperger E.; Daniel C.; Marian C. M. Spin-vibronic mechanism for intersystem crossing. Chem. Rev. 2018, 118, 6975 10.1021/acs.chemrev.7b00617. 29558159

[ref41] Terenziani F.; Painelli A.; Katan C.; Charlot M.; Blanchard-Desce M. Charge instability in quadrupolar chromophores: Symmetry breaking and solvatochromism. J. Am. Chem. Soc. 2006, 128, 15742 10.1021/ja064521j. 17147384

[ref42] Hyun H.; Owens E. A.; Narayana L.; Wada H.; Gravier J.; Bao K.; Frangioni J. V.; Choi H. S.; Henary M. Central C–C bonding increases optical and chemical stability of NIR fluorophores. RSC Adv. 2014, 4, 58762 10.1039/C4RA11225C. 25530846 PMC4267294

[ref43] Ebaston T.; Nakonechny F.; Talalai E.; Gellerman G.; Patsenker L. Iodinated xanthene-cyanine NIR dyes as potential photosensitizers for antimicrobial photodynamic therapy. Dyes Pigm. 2021, 184, 108854 10.1016/j.dyepig.2020.108854.

[ref44] Pham W.; Medarova Z.; Moore A. Synthesis and application of a water-soluble near-infrared dye for cancer detection using optical imaging. Bioconjugate Chem. 2005, 16, 735 10.1021/bc049700+. 15898745

[ref45] Song F.; Peng X.; Lu E.; Zhang R.; Chen X.; Song B. Syntheses, spectral properties and photostabilities of novel water-soluble near-infrared cyanine dyes. J. Photochem. Photobiol., A 2004, 168, 53 10.1016/j.jphotochem.2004.05.012.

[ref46] Hansch C.; Leo A.; Taft R. A survey of Hammett substituent constants and resonance and field parameters. Chem. Rev. 1991, 91, 165 10.1021/cr00002a004.

[ref47] Matikonda S. S.; Hammersley G.; Kumari N.; Grabenhorst L.; Glembockyte V.; Tinnefeld P.; Ivanic J.; Levitus M.; Schnermann M. J. Impact of cyanine conformational restraint in the near-infrared range. J. Org. Chem. 2020, 85, 5907 10.1021/acs.joc.0c00236. 32275153 PMC8459201

[ref48] Wang L.; Jin J.; Chen X.; Fan H.-H.; Li B. K. F.; Cheah K.-W.; Ding N.; Ju S.; Wong W.-T.; Li C. A cyanine based fluorophore emitting both single photon near-infrared fluorescence and two-photon deep red fluorescence in aqueous solution. Org. Biomol. Chem. 2012, 10, 5366 10.1039/c2ob25619c. 22710825

[ref49] Thavornpradit S.; Usama S. M.; Park G. K.; Shrestha J. P.; Nomura S.; Baek Y.; Choi H. S.; Burgess K. QuatCy: A Heptamethine Cyanine Modification With Improved Characteristics. Theranostics 2019, 9, 2856 10.7150/thno.33595. 31244928 PMC6568187

[ref50] Landsman M.; Kwant G.; Mook G.; Zijlstra W. Light-absorbing properties, stability, and spectral stabilization of indocyanine green. J. Appl. Physiol. 1976, 40, 575 10.1152/jappl.1976.40.4.575. 776922

[ref51] Filatov M.; Huix-Rotllant M. Assessment of density functional theory based ΔSCF (self-consistent field) and linear response methods for longest wavelength excited states of extended π-conjugated molecular systems. J. Chem. Phys. 2014, 141, 024112 10.1063/1.4887087. 25028004

[ref52] Peach M. J. G.; Williamson M. J.; Tozer D. J. Influence of Triplet Instabilities in TDDFT. J. Chem. Theory Comput. 2011, 7, 3578 10.1021/ct200651r. 26598256

[ref53] Kautsky H.; de Bruijn H. Frontier Orbitals, Combustion and Redox Transfer from a Fermionic-Bosonic Orbital Perspective. Naturwissenschaften 1931, 19, 1043 10.1007/BF01516190.

[ref54] Wang Z.; Toffoletti A.; Hou Y.; Zhao J.; Barbon A.; Dick B. Insight into the drastically different triplet lifetimes of BODIPY obtained by optical/magnetic spectroscopy and theoretical computations. Chem. Sci. 2021, 12, 2829 10.1039/D0SC05494A. PMC8179375 34164047

[ref55] Semenova O.; Kobzev D.; Yazbak F.; Nakonechny F.; Kolosova O.; Tatarets A.; Gellerman G.; Patsenker L. Unexpected effect of iodine atoms in heptamethine cyanine dyes on the photodynamic eradication of Gram-positive and Gram-negative pathogens. Dyes Pigm. 2021, 195, 109745 10.1016/j.dyepig.2021.109745.

[ref56] Krieg M.; Redmond R. W. Photophysical properties of 3, 3′-dialkylthiacarbocyanine dyes in homogeneous solution. Photochem. Photobiol. 1993, 57, 472 10.1111/j.1751-1097.1993.tb02321.x. 8475181

[ref57] Usama S. M.; Thavornpradit S.; Burgess K. Optimized heptamethine cyanines for photodynamic therapy. ACS Appl. Bio Mater. 2018, 1, 1195 10.1021/acsabm.8b00414. 34996160

[ref58] James N. S.; Chen Y.; Joshi P.; Ohulchanskyy T. Y.; Ethirajan M.; Henary M.; Strekowsk L.; Pandey R. K. Evaluation of polymethine dyes as potential probes for near infrared fluorescence imaging of tumors: Part-1. Theranostics 2013, 3, 692 10.7150/thno.5922. 24019854 PMC3767116

[ref59] Tanielian C.; Golder L.; Wolff C. Production and quenching of singlet oxygen by the sensitizer in dye-sensitized photo-oxygenations. J. Photochem. 1984, 25, 117 10.1016/0047-2670(84)87016-1.

[ref60] Yoshiharu U. Determination Of Quantum Yield Of Singlet Oxygen Formation By Photosensitization. Chem. Lett. 1973, 2, 743 10.1246/cl.1973.743.

[ref61] Gorka A. P.; Schnermann M. J. Harnessing cyanine photooxidation: from slowing photobleaching to near-IR uncaging. Curr. Opin. Chem. Biol. 2016, 33, 117 10.1016/j.cbpa.2016.05.022. 27348157 PMC7383357

[ref62] Strehmel B.; Schmitz C.; Kütahya C.; Pang Y.; Drewitz A.; Mustroph H. Photophysics and photochemistry of NIR absorbers derived from cyanines: key to new technologies based on chemistry 4.0. Beilstein J. Org. Chem. 2020, 16, 415 10.3762/bjoc.16.40. 32273905 PMC7113544

[ref63] Rüttger F.; Mindt S.; Golz C.; Alcarazo M.; John M. Isomerization and Dimerization of Indocyanine Green and a Related Heptamethine Dye. Eur. J. Org. Chem. 2019, 2019, 4791 10.1002/ejoc.201900715.

[ref64] Chen P.; Sun S.; Hu Y.; Qian Z.; Zheng D. Structure and solvent effect on the photostability of indolenine cyanine dyes. Dyes Pigm. 1999, 41, 227 10.1016/S0143-7208(98)00088-6.

[ref65] Feng L.; Chen W.; Ma X.; Liu S. H.; Yin J. Near-infrared heptamethine cyanines (Cy7): from structure, property to application. Org. Biomol. Chem. 2020, 18, 9385 10.1039/D0OB01962C. 33191410

[ref66] Bunce N. J. In Organic Photochemistry and Photobiology; Horspool, W. M., Song P.-S., Eds.; CRC Press: Boca Raton, 1994, p 1181.

[ref67] Luo Y.-R. Handbook of Bond Dissociation Energies in Organic Compounds; CRC Press: Boca Raton, 2002, 10.1201/9781420039863.

[ref68] Nastasa V.; Pascu A.; Boni M.; Smarandache A.; Staicu A.; Pascu M. Insights into the photophysics of zinc phthalocyanine and photogenerated singlet oxygen in DMSO-water mixture. Colloids Surf., A 2016, 505, 197 10.1016/j.colsurfa.2016.04.050.

[ref69] El-Sayed M. A. Triplet state. Its radiative and nonradiative properties. Acc. Chem. Res. 1968, 1, 8 10.1021/ar50001a002.

[ref70] Pokhilko P.; Krylov A. I. Quantitative El-Sayed Rules for Many-Body Wave Functions from Spinless Transition Density Matrices. J. Phys. Chem. Lett. 2019, 10, 4857 10.1021/acs.jpclett.9b02120. 31386377

[ref71] Albrecht A. C. Vibronic—Spin-Orbit Perturbations and the Assignment of the Lowest Triplet State of Benzene. J. Chem. Phys. 1963, 38, 354 10.1063/1.1733665.

[ref72] Perun S.; Tatchen J.; Marian C. M. Singlet and triplet excited states and intersystem crossing in free-base porphyrin: TDDFT and DFT/MRCI study. ChemPhysChem 2008, 9, 282 10.1002/cphc.200700509. 18189251

[ref73] Minaev B.; Ågren H. Theoretical DFT study of phosphorescence from porphyrins. Chem. Phys. 2005, 315, 215 10.1016/j.chemphys.2005.04.017.

[ref74] Tatchen J.; Gilka N.; Marian C. M. Intersystem crossing driven by vibronic spin–orbit coupling: a case study on psoralen. Phys. Chem. Chem. Phys. 2007, 9, 5209 10.1039/b706410a. 19459284

[ref75] Alarcon E.; Edwards A. M.; Aspee A.; Borsarelli C. D.; Lissi E. A. Photophysics and photochemistry of rose bengal bound to human serum albumin. Photochem. Photobiol. Sci. 2009, 8, 933 10.1039/b901056d. 19582268

[ref76] Hoebeke M.; Damoiseau X. Determination of the singlet oxygen quantum yield of bacteriochlorin a: a comparative study in phosphate buffer and aqueous dispersion of dimiristoyl-L-alpha-phosphatidylcholine liposomes. Photochem. Photobiol. Sci. 2002, 1, 283 10.1039/b201081j. 12661969

[ref77] Muraseccosuardi P.; Gassmann E.; Braun A. M.; Oliveros E. Determination Of The Quantum Yield Of Intersystem Crossing Of Rose Bengal. Helv. Chim. Acta 1987, 70, 1760 10.1002/hlca.19870700712.

[ref78] Fang L.; Liu J. A.; Ju S.; Zheng F. G.; Dong W.; Shen M. R. Experimental and theoretical evidence of enhanced ferromagnetism in sonochemical synthesized BiFeO3 nanoparticles. Appl. Phys. Lett. 2010, 97, 242501 10.1063/1.3525573.

[ref79] Young R. H.; Brewer D.; Keller R. A. Determination Of Rate Constants Of Reaction And Lifetimes Of Singlet Oxygen In Solution By A Flash-Photolysis Technique. J. Am. Chem. Soc. 1973, 95, 375 10.1021/ja00783a012.

[ref80] Entradas T.; Waldron S.; Volk M. The detection sensitivity of commonly used singlet oxygen probes in aqueous environments. J. Photochem. Photobiol., B 2020, 204 10.1016/j.jphotobiol.2020.111787. 31958676

[ref81] Wilkinson F.; Helman W. P.; Ross A. B. Quantum Yields For The Photosensitized Formation Of The Lowest Electronically Excited Singlet-state Of Molecular Oxygen In Solution. J. Phys. Chem. Ref. Data 1993, 22, 113 10.1063/1.555934.

[ref82] Atkinson K. M.; Morsby J. J.; Kommidi S. S. R.; Smith B. D. Generalizable synthesis of bioresponsive near-infrared fluorescent probes: sulfonated heptamethine cyanine prototype for imaging cell hypoxia. Org. Biomol. Chem. 2021, 19, 4100 10.1039/D1OB00426C. 33978049 PMC8121178

[ref83] Kumar S.; Watkins D. L.; Fujiwara T. A tailored spirooxazine dimer as a photoswitchable binding tool. Chem. Commun. 2009, 4369 10.1039/b909496b. 19597595

[ref84] Choi H. S.; Nasr K.; Alyabyev S.; Feith D.; Lee J. H.; Kim S. H.; Ashitate Y.; Hyun H.; Patonay G.; Strekowski L.; Henary M.; Frangioni J. V. Synthesis and In Vivo Fate of Zwitterionic Near-Infrared Fluorophores. Angew. Chem., Int. Ed. 2011, 50, 6258 10.1002/anie.201102459. PMC3128676 21656624

[ref85] Schulz-Senft M.; Gates P. J.; Sönnichsen F. D.; Staubitz A. Diversely halogenated spiropyrans - Useful synthetic building blocks for a versatile class of molecular switches. Dyes Pigm. 2017, 136, 292 10.1016/j.dyepig.2016.08.039.

[ref86] Strekowski L.; Mason J. C.; Lee H.; Say M.; Patonay G. Water-soluble pH-sensitive 2,6-bis(substituted ethylidene)-cyclohexanone/hydroxy cyanine dyes that absorb in the visible/near-infrared regions. J. Heterocycl. Chem. 2004, 41, 227 10.1002/jhet.5570410213.

[ref87] Miertuš S.; Scrocco E.; Tomasi J. Electrostatic interaction of a solute with a continuum. A direct utilizaion of AB initio molecular potentials for the prevision of solvent effects. Chem. Phys. 1981, 55, 117 10.1016/0301-0104(81)85090-2.

[ref88] Körzdörfer T.; Sears J. S.; Sutton C.; Brédas J.-L. Long-range corrected hybrid functionals for π-conjugated systems: Dependence of the range-separation parameter on conjugation length. J. Chem. Phys. 2011, 135, 204107 10.1063/1.3663856. 22128928

[ref89] Grimme S. A simplified Tamm-Dancoff density functional approach for the electronic excitation spectra of very large molecules. J. Chem. Phys. 2013, 138, 244104 10.1063/1.4811331. 23822224

[ref90] Neese F. Software update: the ORCA program system, version 4.0. Wiley Interdiscip. Rev.: Comput. Mol. Sci. 2018, 8, e1327 10.1002/wcms.1327.

[ref91] Neese F. The ORCA program system. Wiley Interdiscip. Rev.: Comput. Mol. Sci. 2012, 2, 73 10.1002/wcms.81.

[ref92] Neese F.; Wennmohs F.; Becker U.; Riplinger C. The ORCA quantum chemistry program package. J. Chem. Phys. 2020, 152, 224108 10.1063/5.0004608. 32534543

[ref93] Sandhoefer B.; Neese F. One-electron contributions to the g-tensor for second-order Douglas–Kroll–Hess theory. J. Chem. Phys. 2012, 137, 094102 10.1063/1.4747454. 22957550

[ref94] Neese F. Efficient and accurate approximations to the molecular spin-orbit coupling operator and their use in molecular g-tensor calculations. J. Chem. Phys. 2005, 122, 034107 10.1063/1.1829047. 15740192

