# Supplementary material for: Spin–Vibronic Control of Intersystem Crossing in Iodine-Substituted Heptamethine Cyanines
Source: J Org Chem. 2023 May 5;88(11):6716–28. doi: 10.1021/acs.joc.3c00005 (PMC10242761; doi:10.1021/acs.joc.3c00005)
Supplement: Supplementary file 1 — jo3c00005_si_001.pdf [file jo3c00005_si_001.pdf]

Supporting Information

Spin-Vibronic Control of Intersystem Crossing in Iodine-Substituted Heptamethine Cyanines

Radek Tovtik,[†,§,¶] Eva Muchová,[‡,¶] Lenka Štacková,[†,§] Petr Slavíček,[‡,*] Petr Klán[†,§,*]

[†] Department of Chemistry, Faculty of Science, Masaryk University, Kamenice 5, 625 00 Brno, Czech Republic

[§] RECETOX, Faculty of Science, Masaryk University, Kamenice 5, 625 00 Brno, Czech Republic

[‡] Department of Physical Chemistry, University of Chemistry and Technology, Prague, Technická 5, 166 28 Prague 6, Czech Republic

* Petr.Slavicek@vscht.cz; klan@sci.muni.cz

[¶] These authors contributed equally to this work.

Table of Contents

UV-VIS Absorption and Emission Spectra S2
NMR Spectra S5
Frontier Molecular Orbitals S47
Cartesian Coordinates of Calculated Compounds S48
Geometry Changes between Ground and Excited States S60
Excitation Energies in Excited State Geometries S60
Spin-Orbit Matrix Elements and Energy Differences between Singlet and Triplet States S61
Detection of Photoproducts S63

UV-VIS Absorption and Emission Spectra

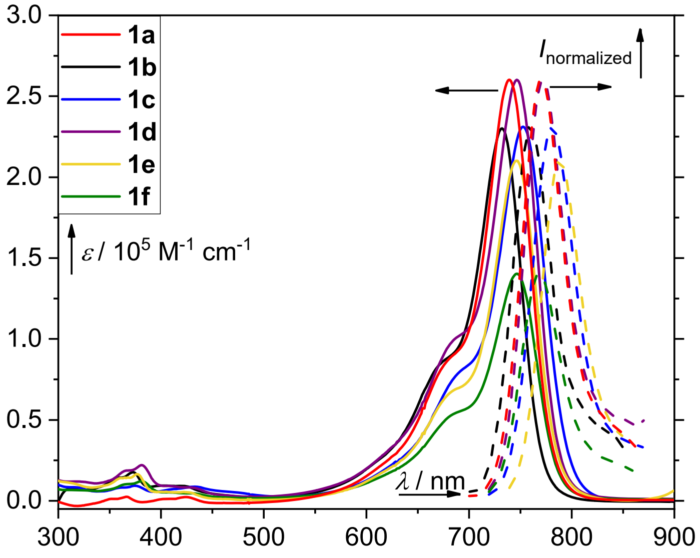

Figure S1: Absorption (—, right y-axis) and normalized emission (---, left y-axis) spectra of **1a**–**f** in methanol.

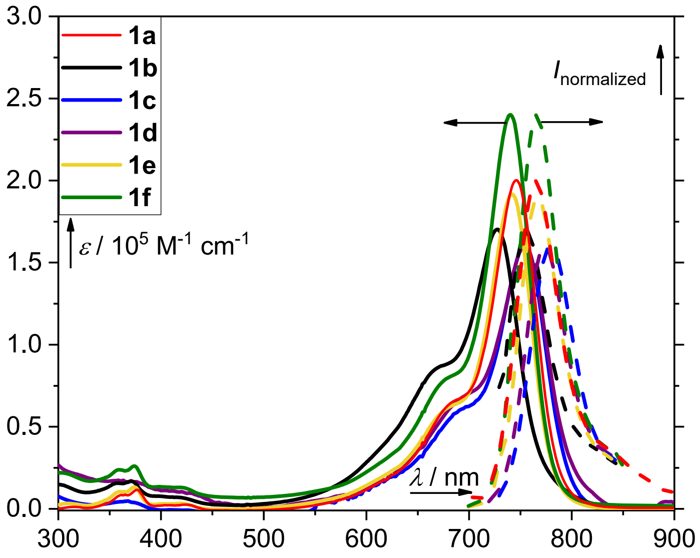

Fig. S2: Absorption (—, right y-axis) and normalized emission (---, left y-axis) spectra of **1a**–**f** in PBS.

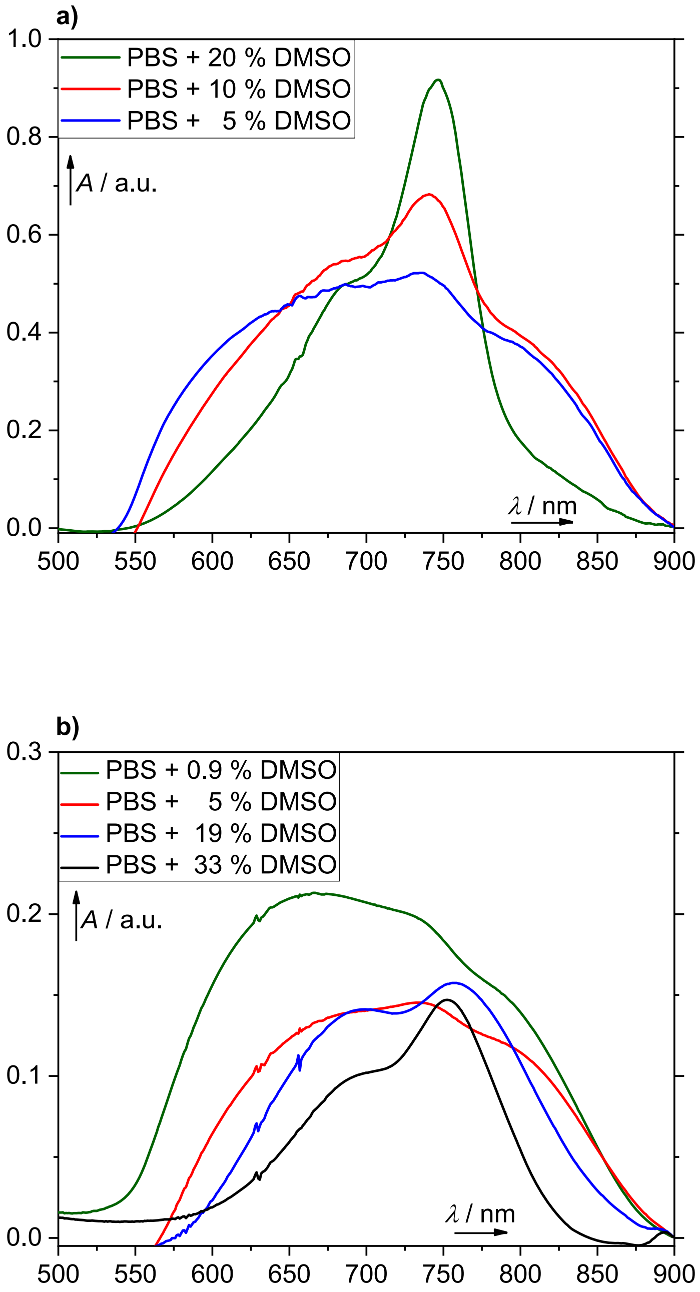
a)
PBS + 20 % DMSO
PBS + 10 % DMSO
PBS + 5 % DMSO
A / a.u.
λ / nm
b)
PBS + 0.9 % DMSO
PBS + 5 % DMSO
PBS + 19 % DMSO
PBS + 33 % DMSO
A / a.u.
λ / nm

c)

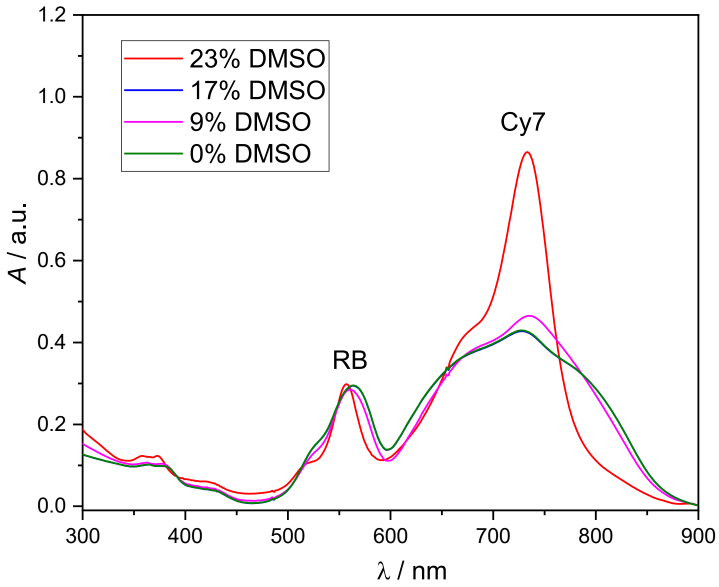

Figure S3. Aggregation of a) **1c** ($c = 1.4 \times 10^{-5}$ M) b) **1d** ($c = 4.8 \times 10^{-6}$ M) in PBS (pH= 7.4, I = 0.1 mM) with different amounts of DMSO (the spikes at 629 and 658 nm are artifacts of a spectrometer) determined by absorption spectroscopy (dye aggregation, a 500–600 nm band, disappears with an increasing amount of DMSO). c) Aggregation of **1b** ($c = 5.8 \times 10^{-6}$ M) in PBS (pH= 7.4, I = 0.1 mM) with different amounts of DMSO in the presence of rose bengal (RB; $c = 4 \times 10^{-6}$ M).

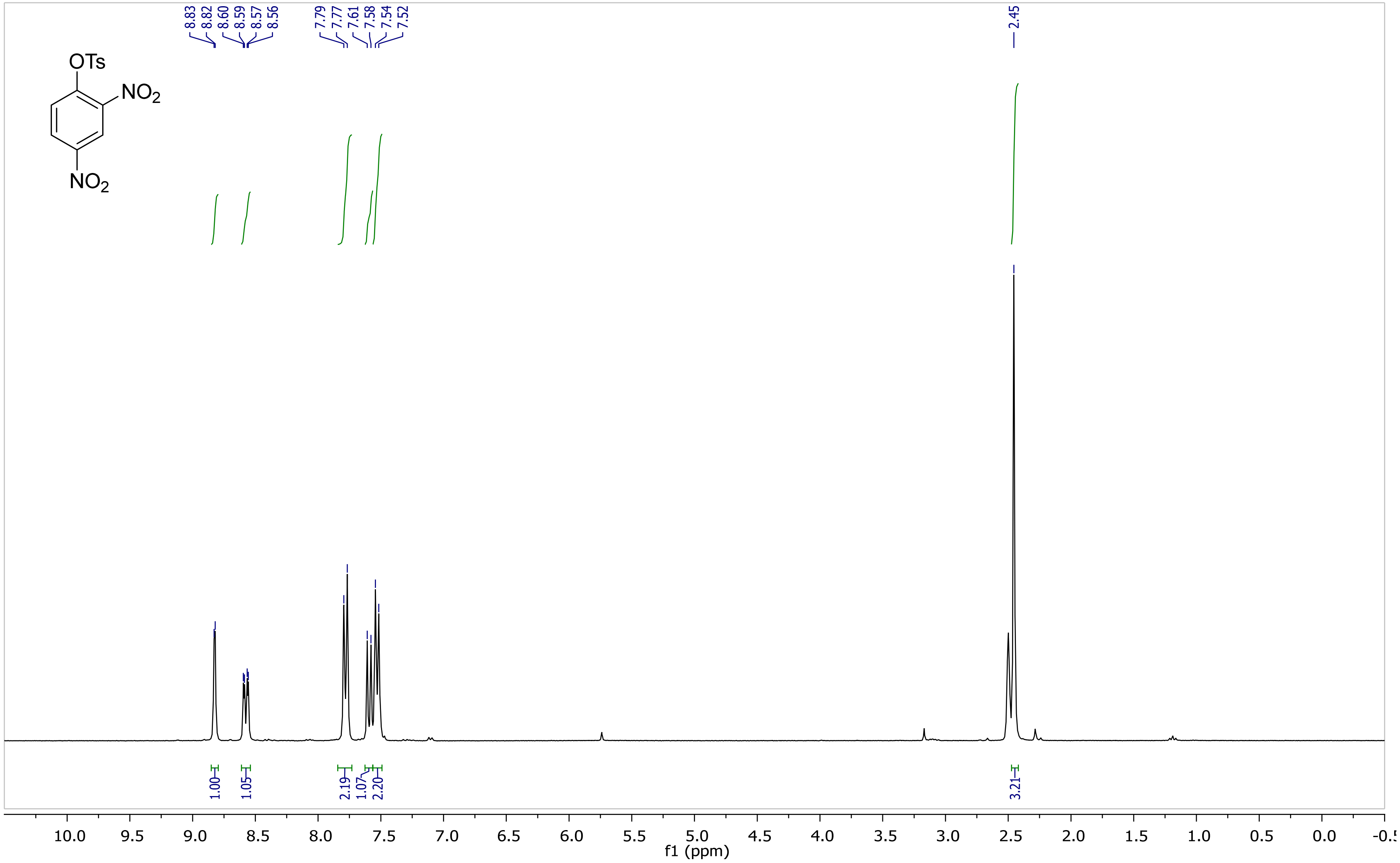

Figure S4. ^{1}H NMR (300 MHz, d_6–DMSO): **4**.

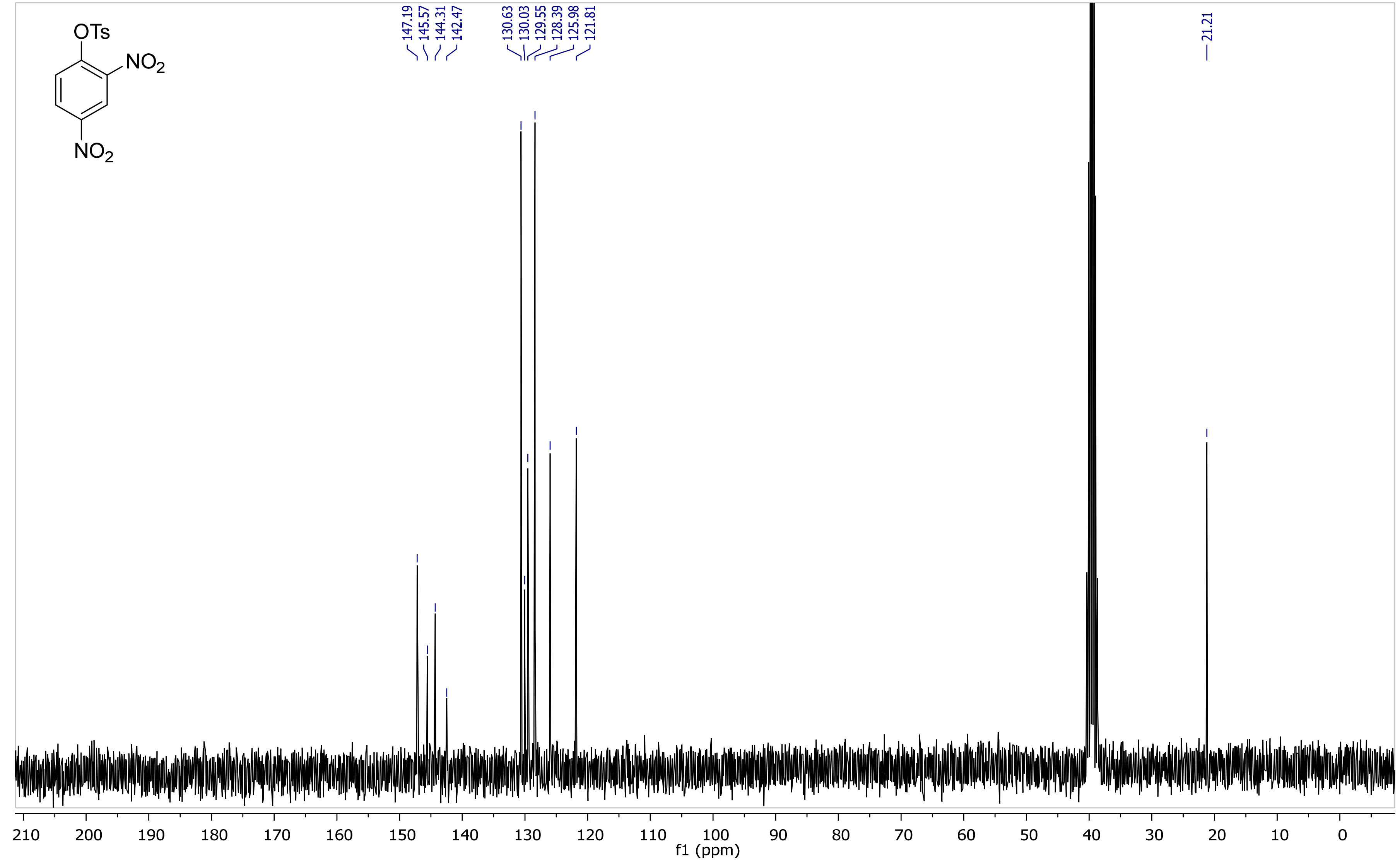

Figure S5. $^{13}C\{^{1}H\}$ NMR (75 MHz, d_6–DMSO): **4**.

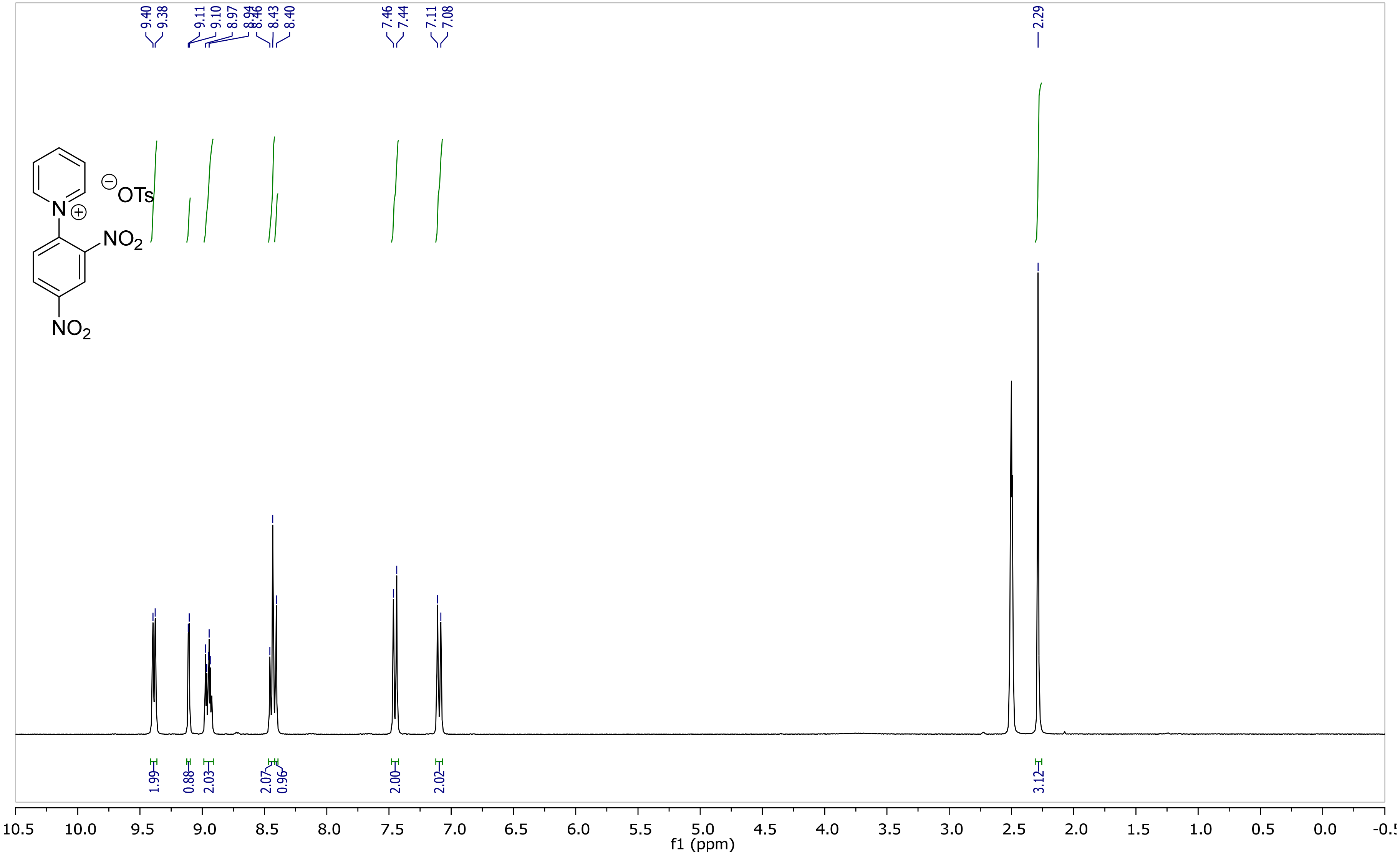

Figure S6. ^{1}H NMR (300 MHz, d_6–DMSO): **5a**.

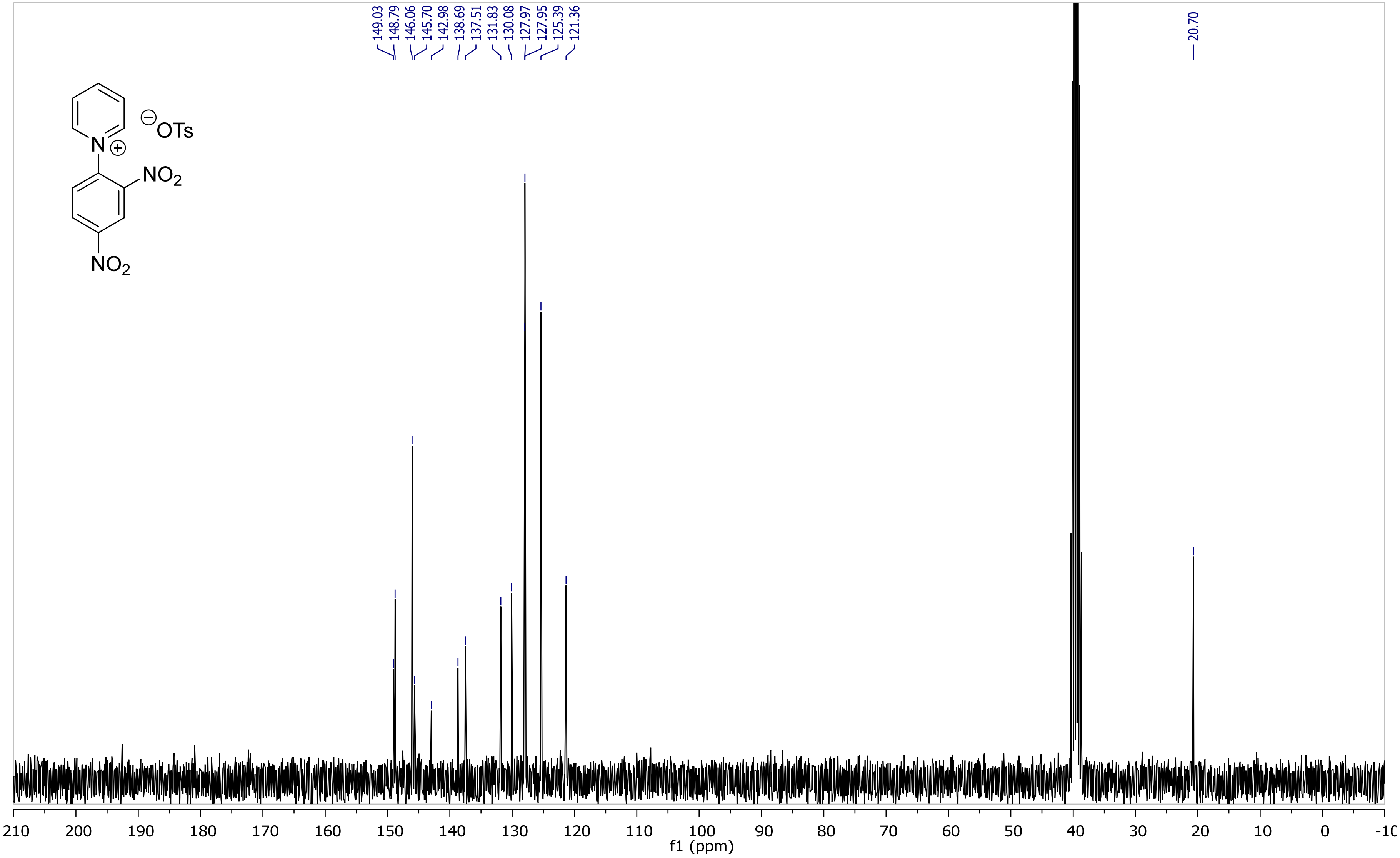

Figure S7. $^{13}C\{^1H\}$ NMR (75 MHz, d_6-DMSO): **5a**.

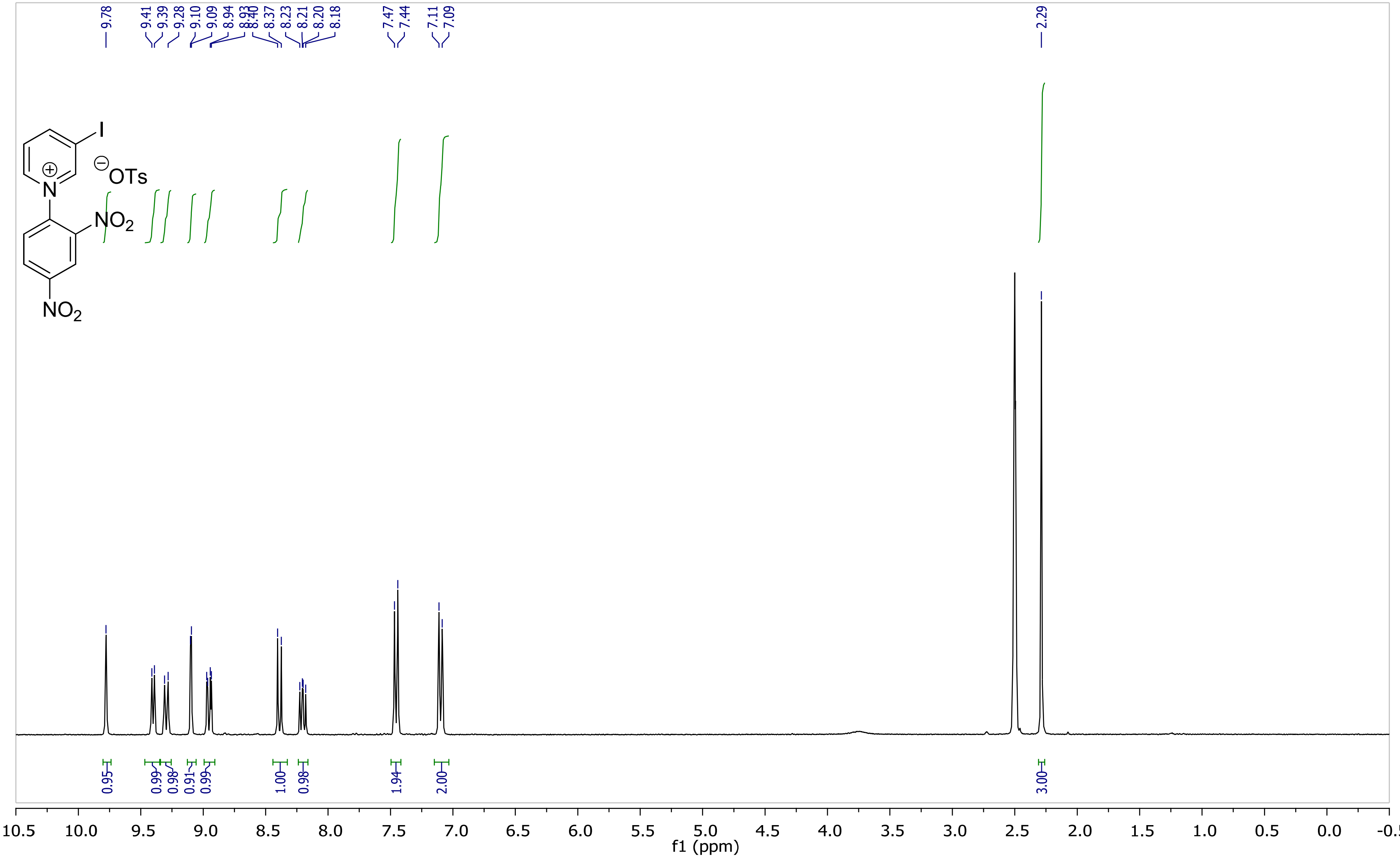

Figure S8. ^{1}H NMR (300 MHz, d_6–DMSO): **5b**.

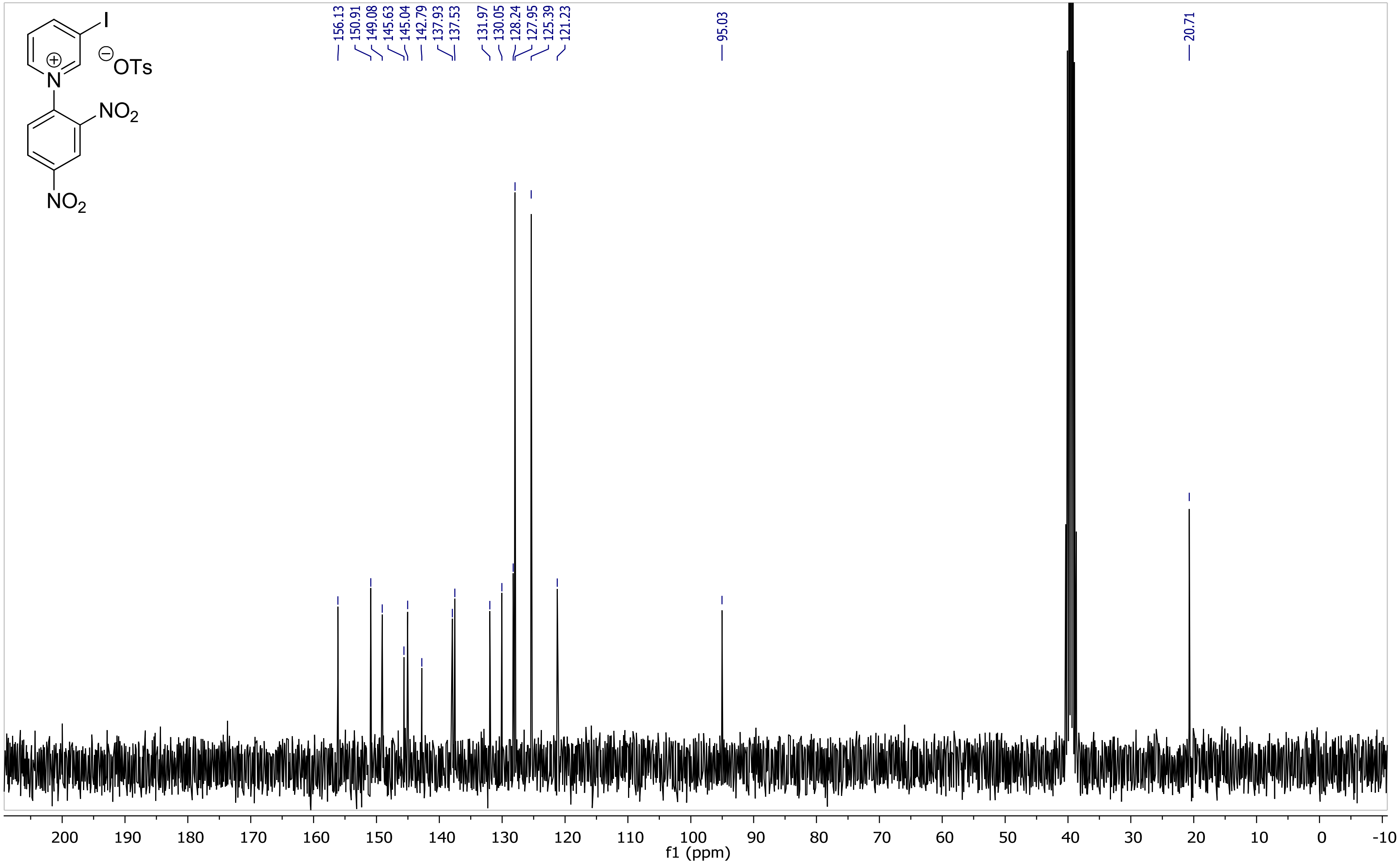

Figure S9. $^{13}C\{^1H\}$ NMR (75 MHz, d_6–DMSO): **5b**.

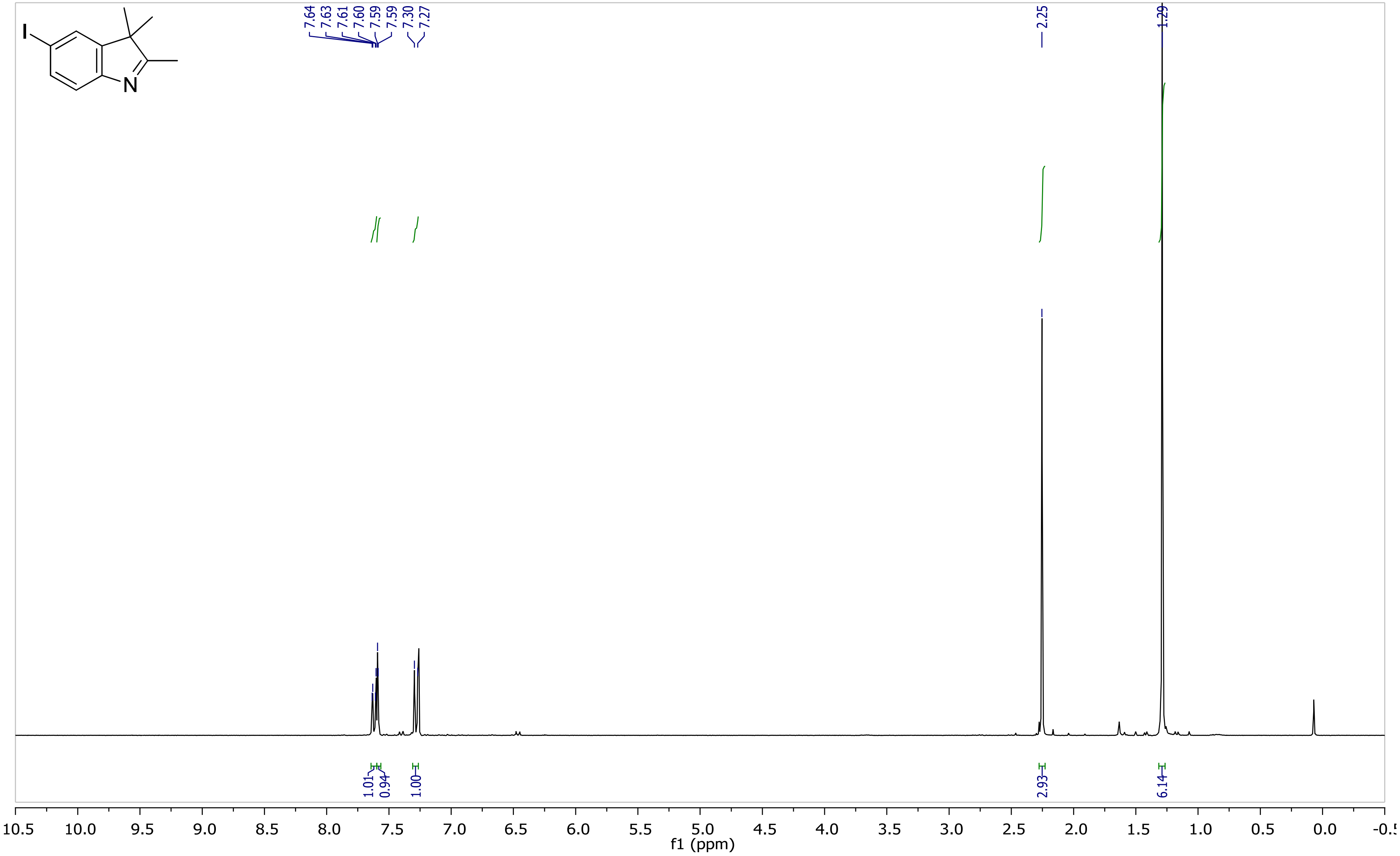

Figure S10. ^{1}H NMR (300 MHz, CCl_3D): **2b**.

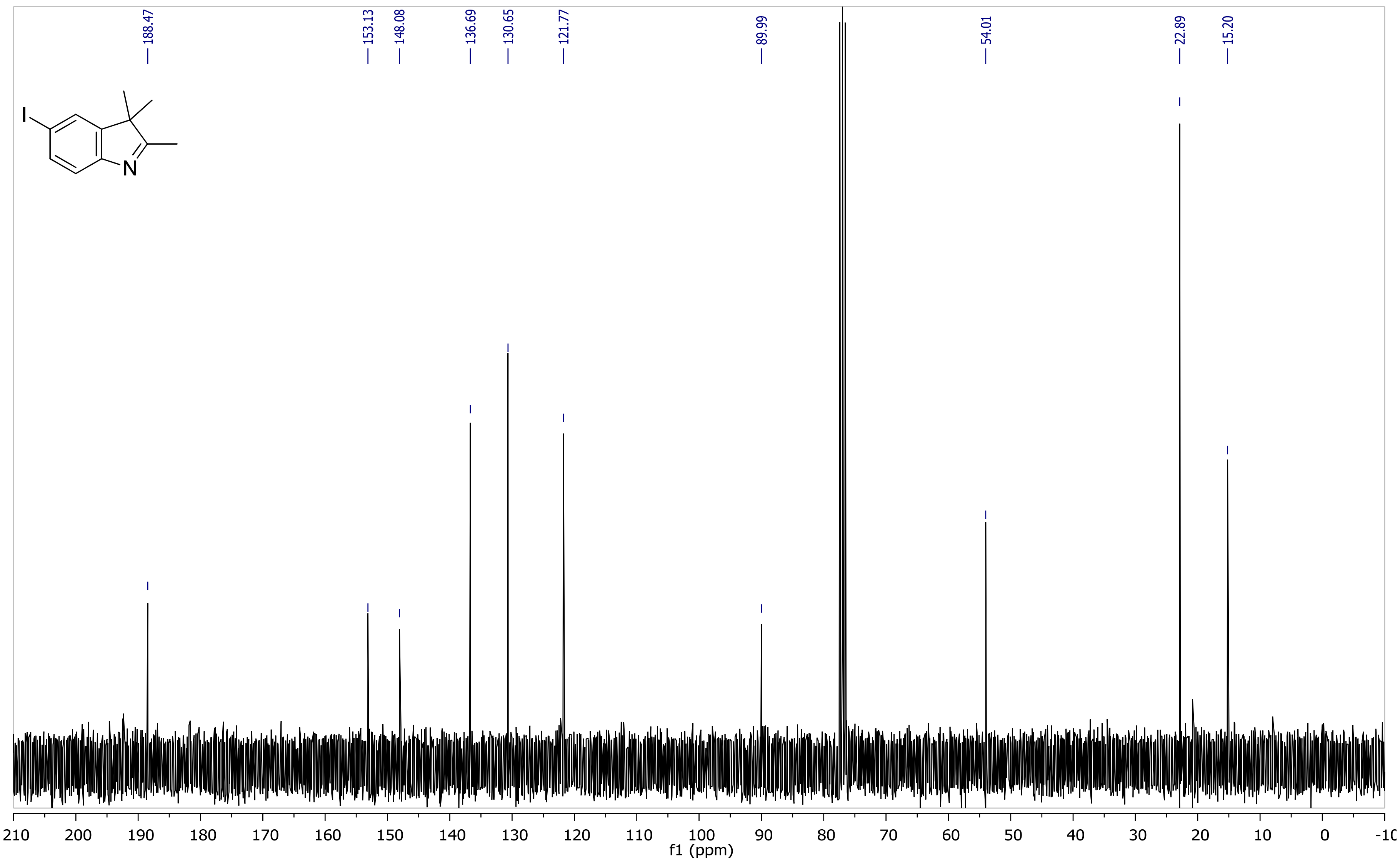

Figure S11. $^{13}C\{^{1}H\}$ NMR (75 MHz, CCl_3D): **2b**.

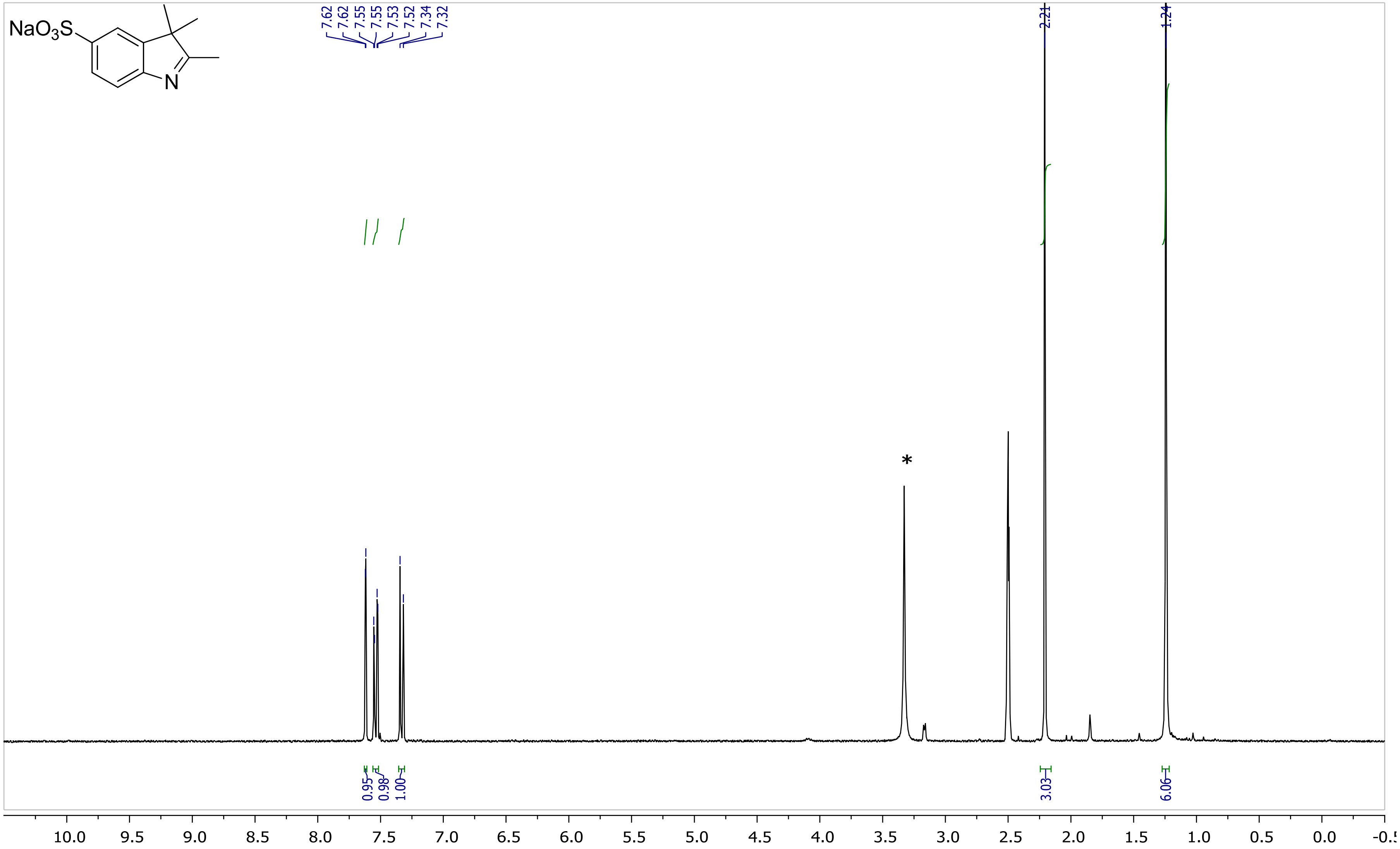

Figure S12. ^{1}H NMR (300 MHz, d_6-DMSO): **2c** (black asterisk denotes water).

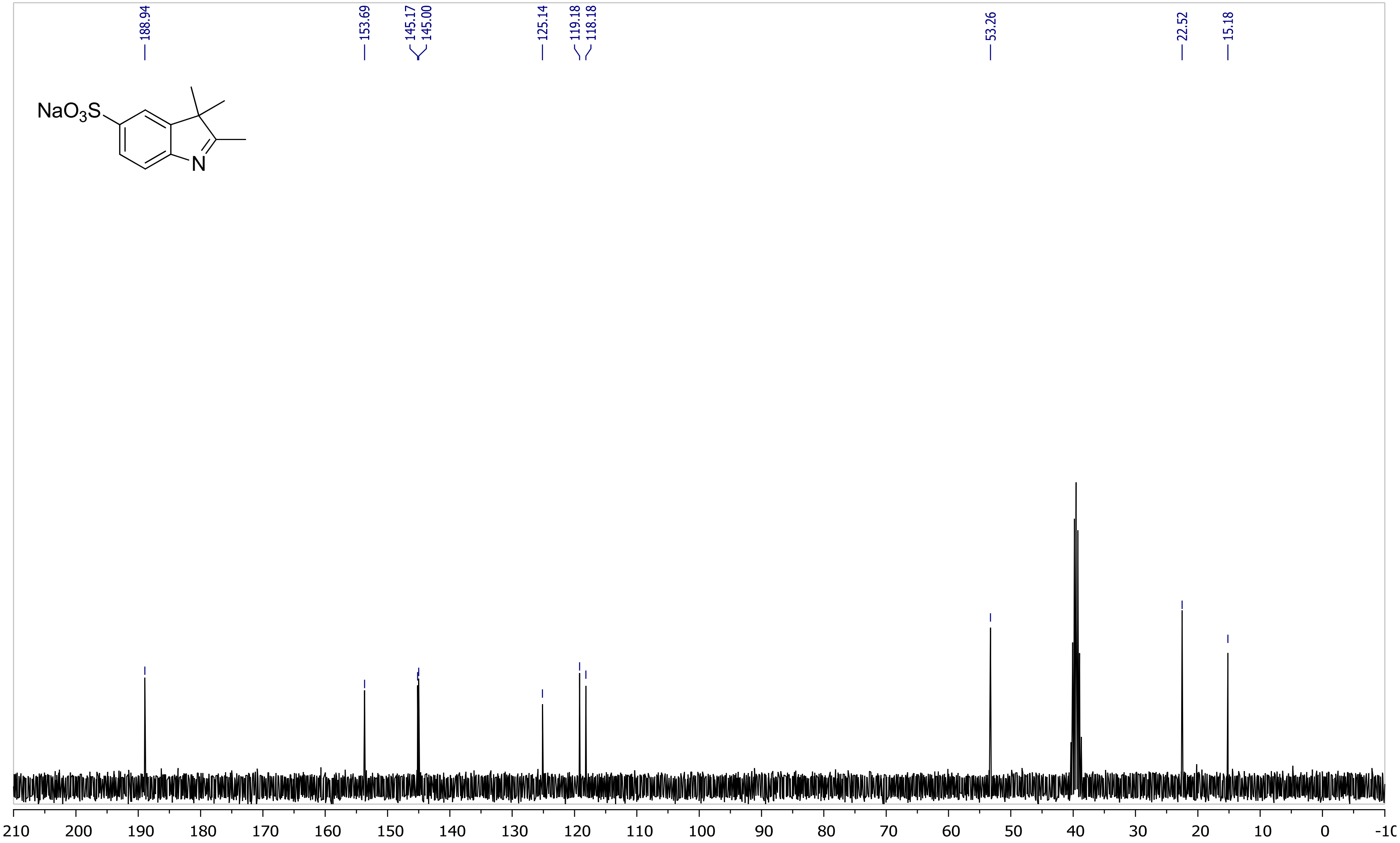

Figure S13. $^{13}C\{^1H\}$ NMR (125 MHz, *d*$_6$-DMSO): **2c**.

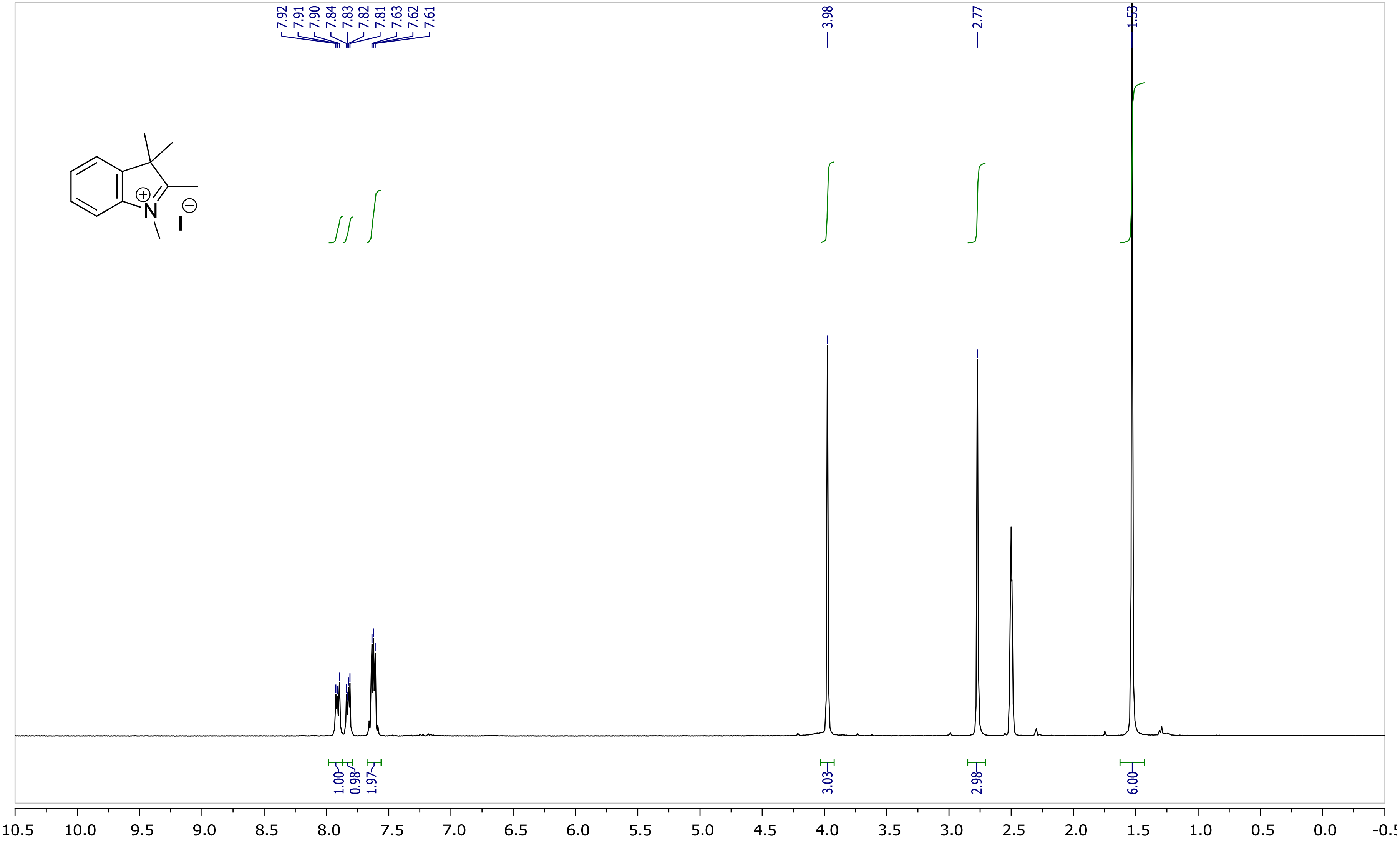

Figure S14. ^{1}H NMR (300 MHz, d_6-DMSO): **3a**.

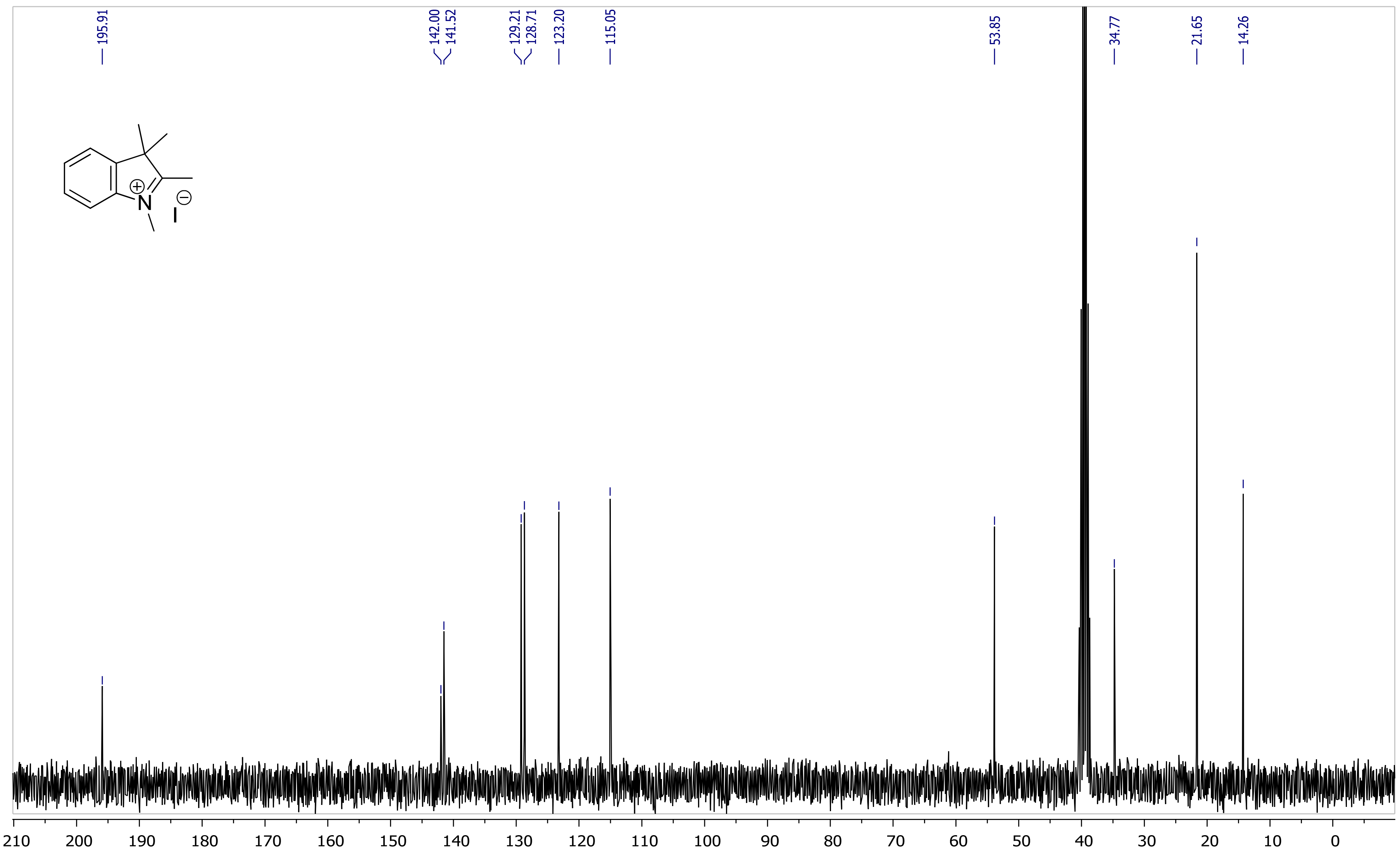

Figure S15. $^{13}C\{^{1}H\}$ NMR (125 MHz, d_6-DMSO): **3a**.

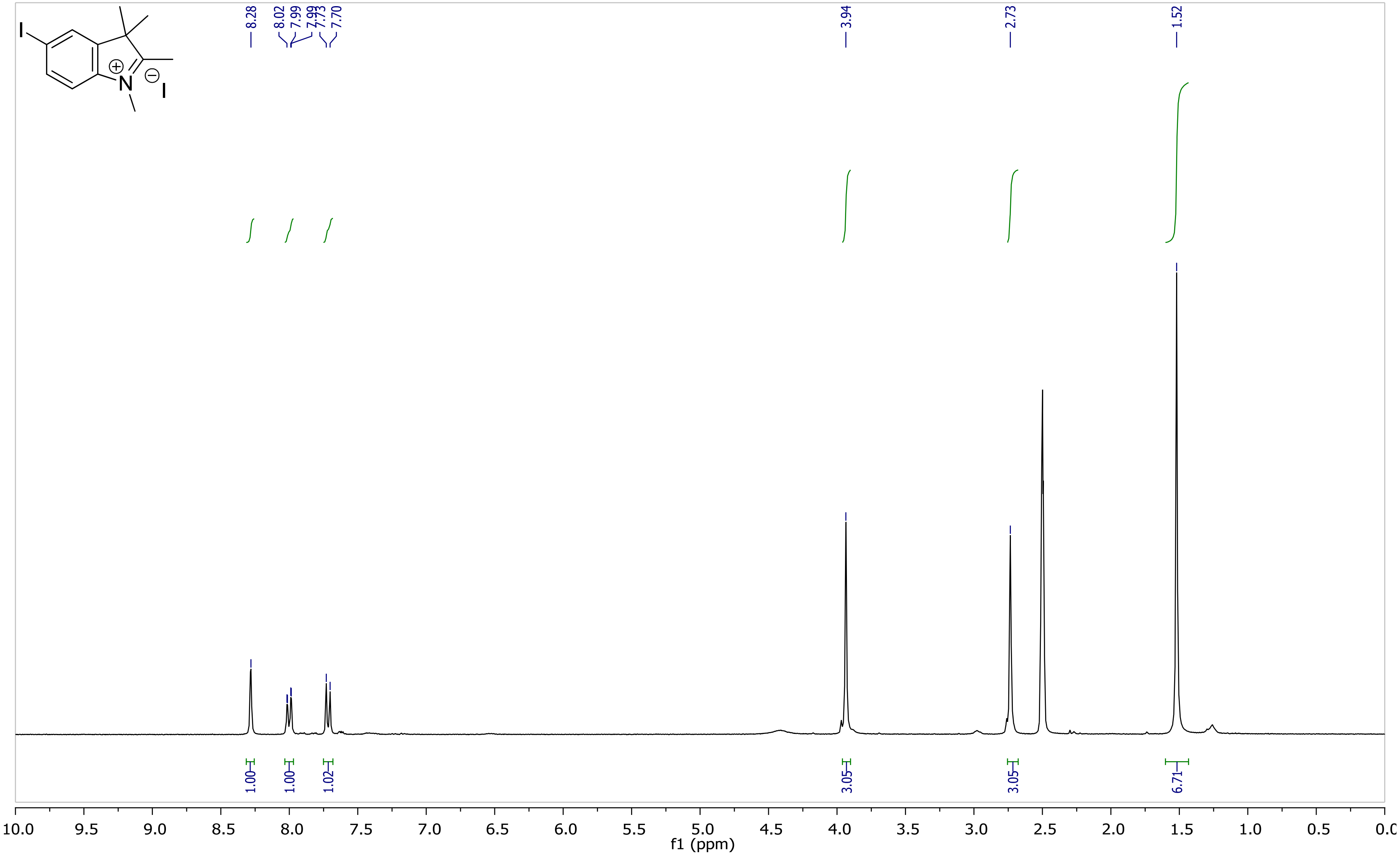

Figure S16. ^{1}H NMR (300 MHz, d_6-DMSO): **3b**.

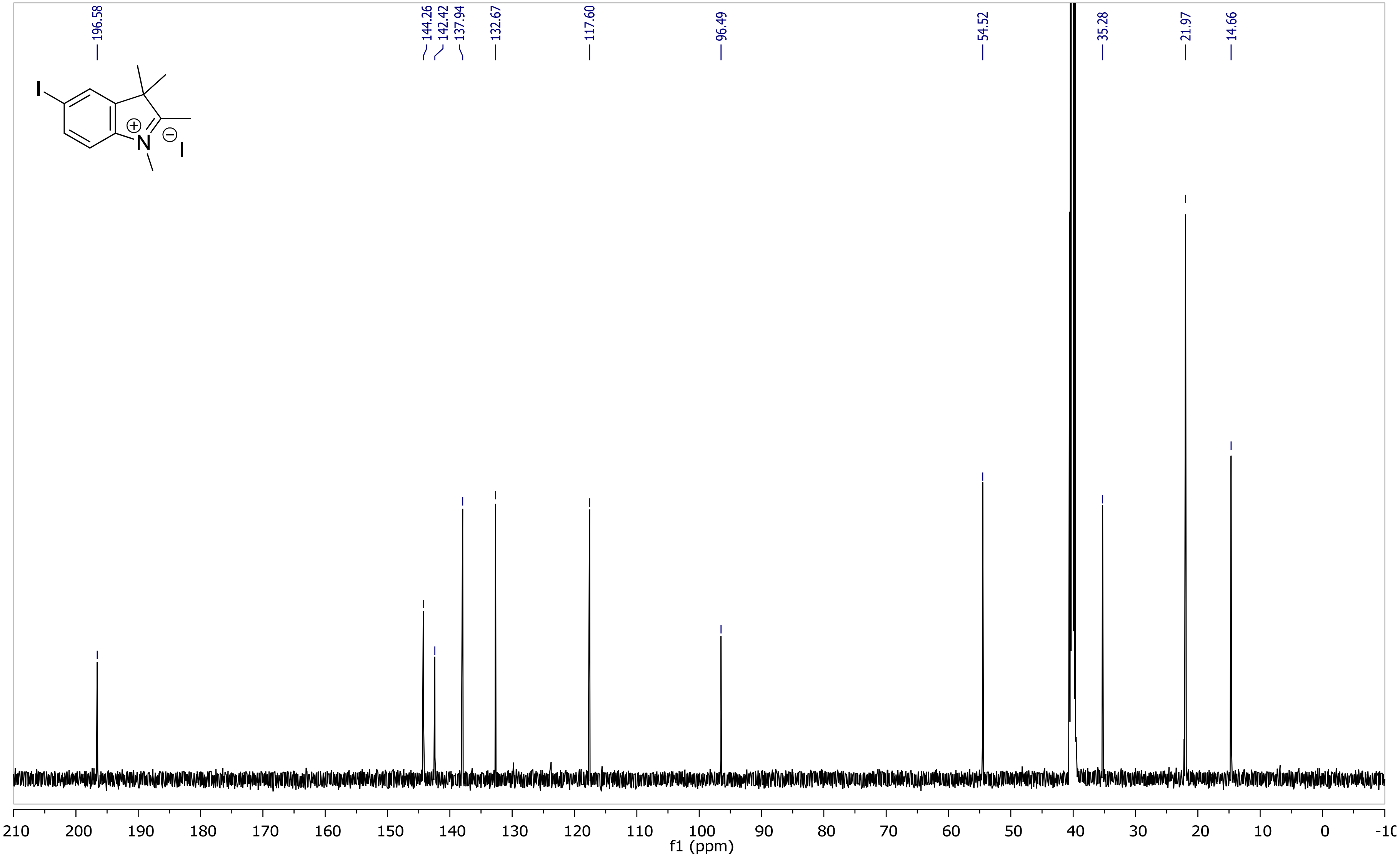

Figure S17. $^{13}C\{^{1}H\}$ NMR (125 MHz, *d*$_6$-DMSO): **3b**.

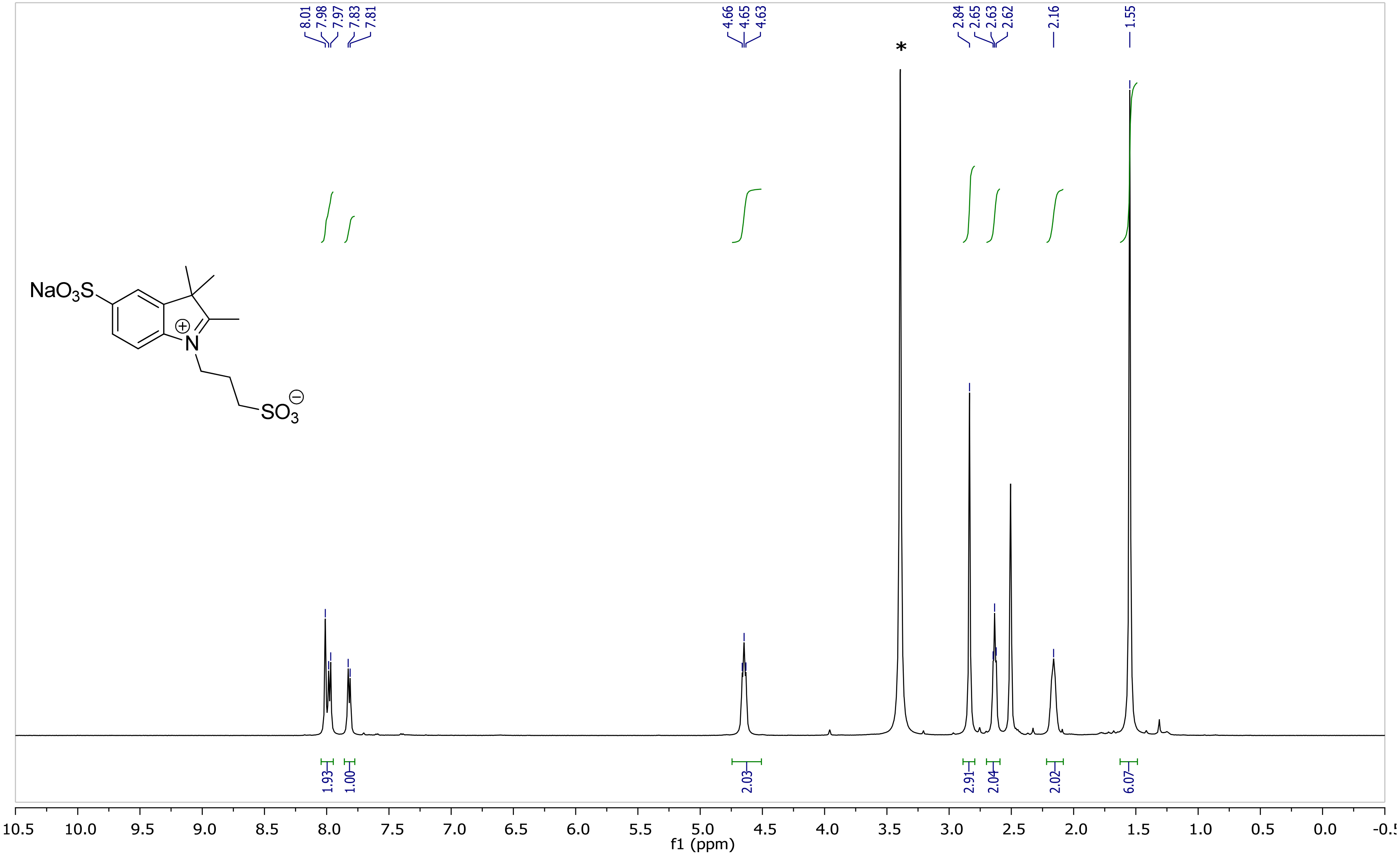

Figure S18. ^{1}H NMR (300 MHz, d_6-DMSO): **3c** (black asterisk denotes water).

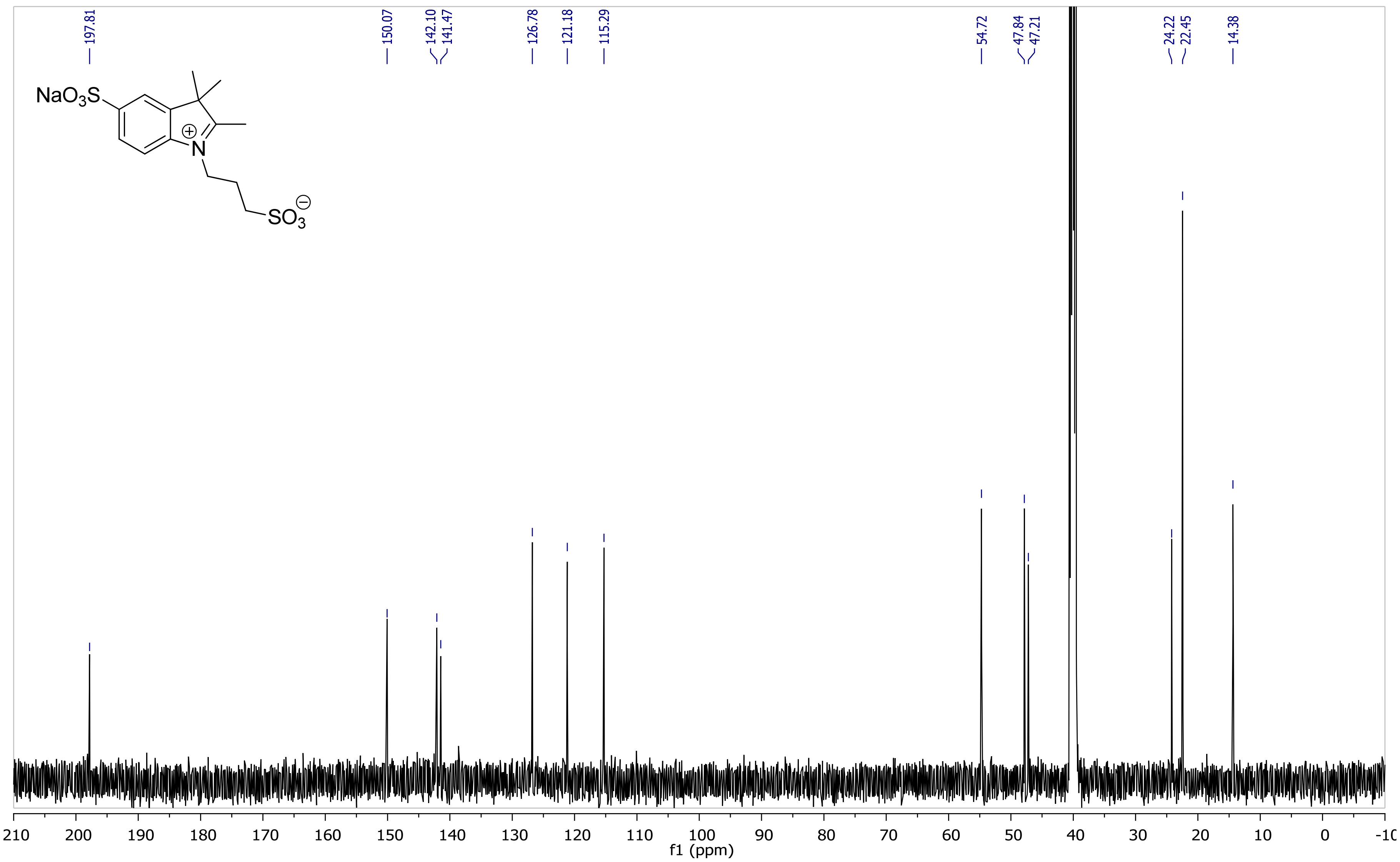

Figure S19. $^{13}C\{^{1}H\}$ NMR (125 MHz, d_6-DMSO): **3c**.

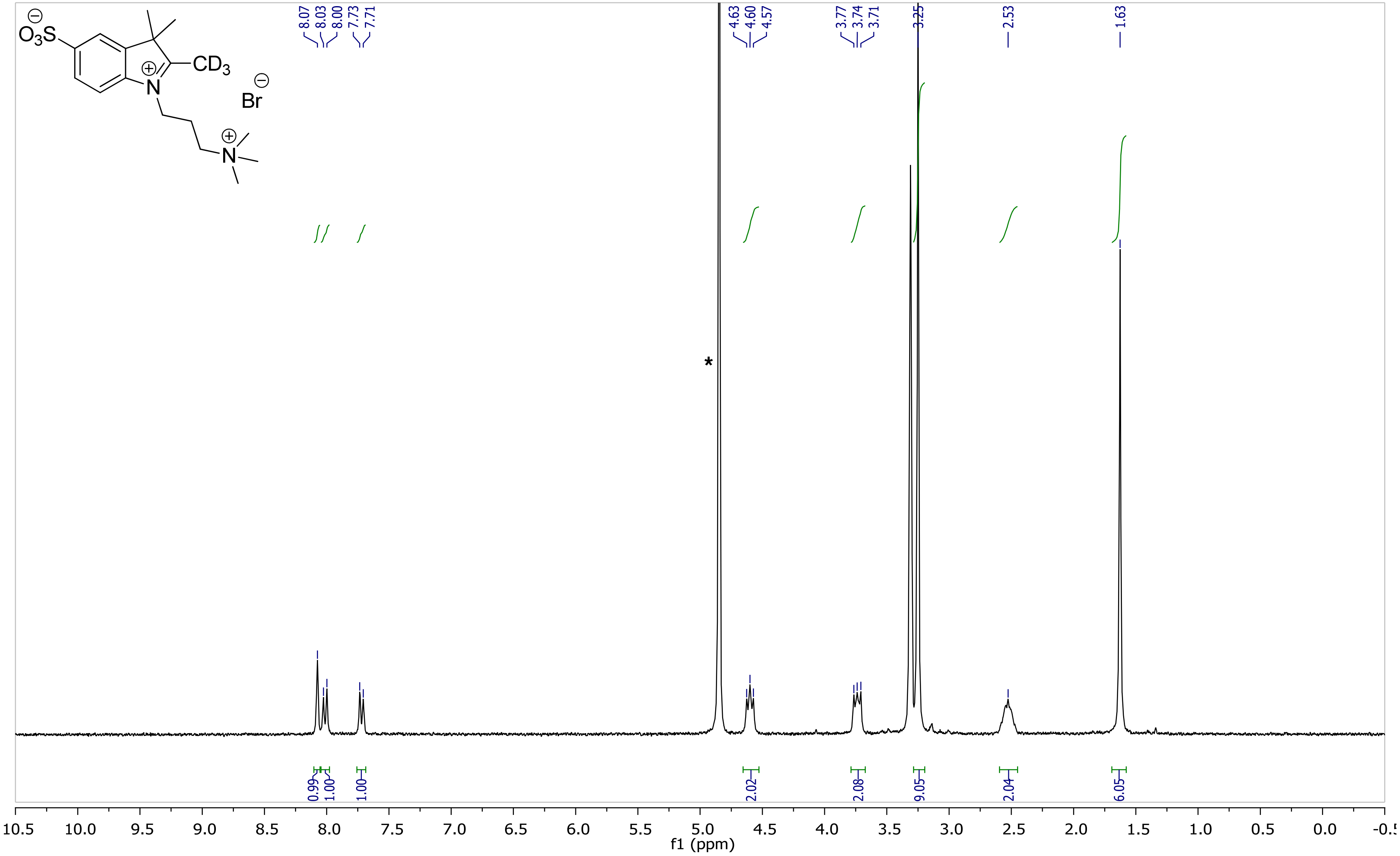

Figure S20. ^{1}H NMR (300 MHz, CD_3OD): **3d** (black asterisk denotes water).

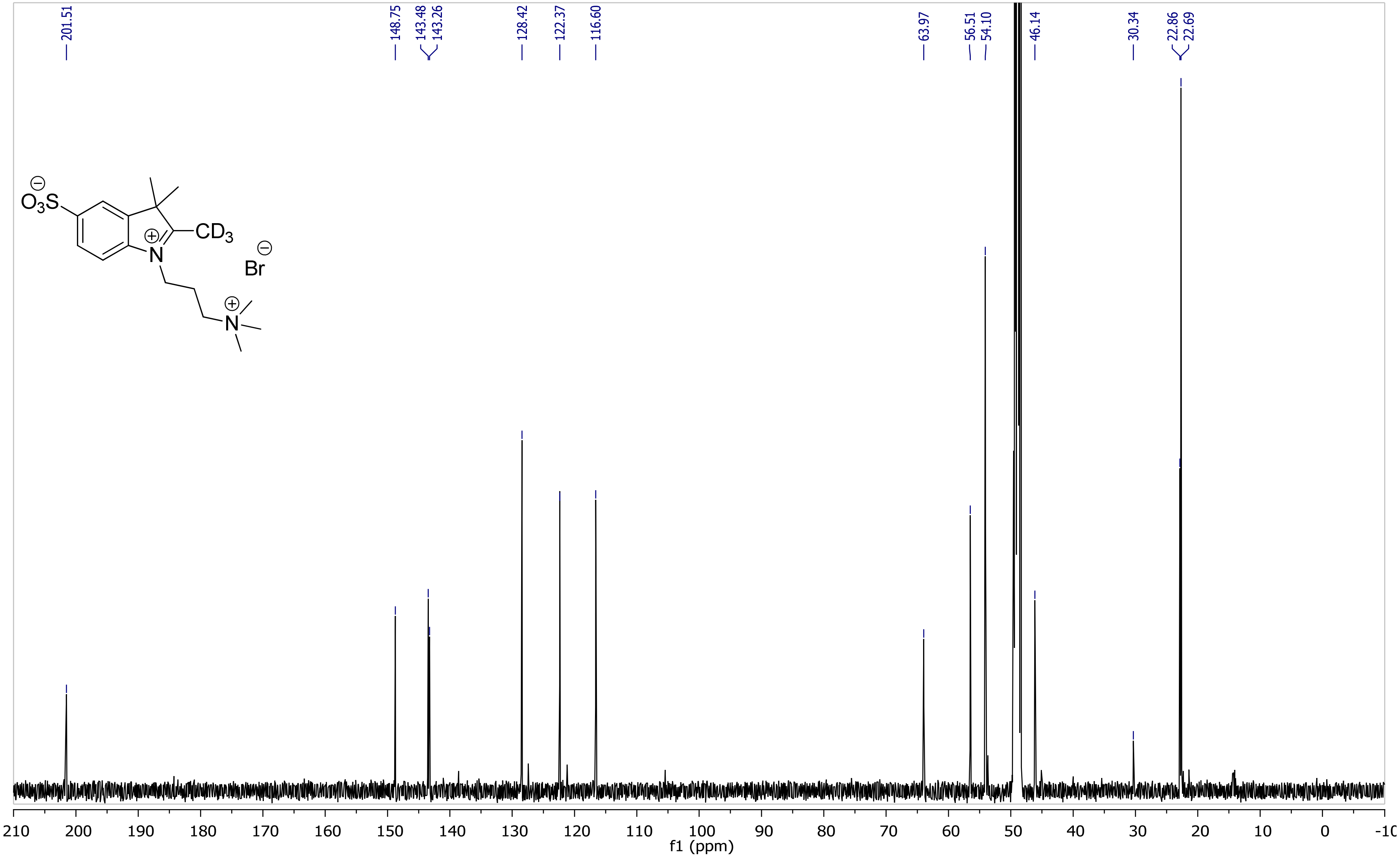

Figure S21. $^{13}C\{^1H\}$ NMR (125 MHz, CD_3OD): **3d**.

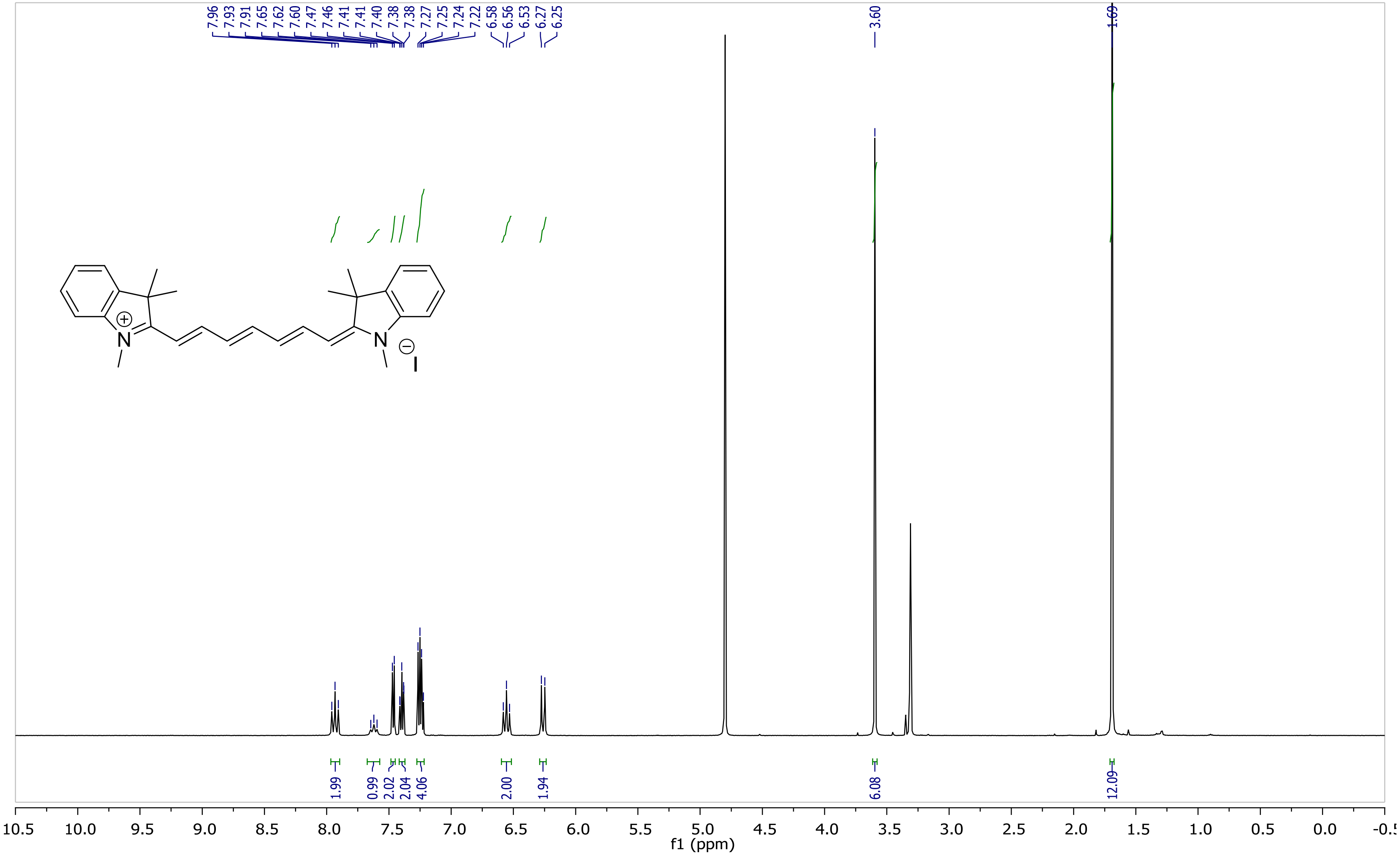

Figure S22. ^{1}H NMR (500 MHz, CD_3OD): **1a** (black asterisk denotes water).

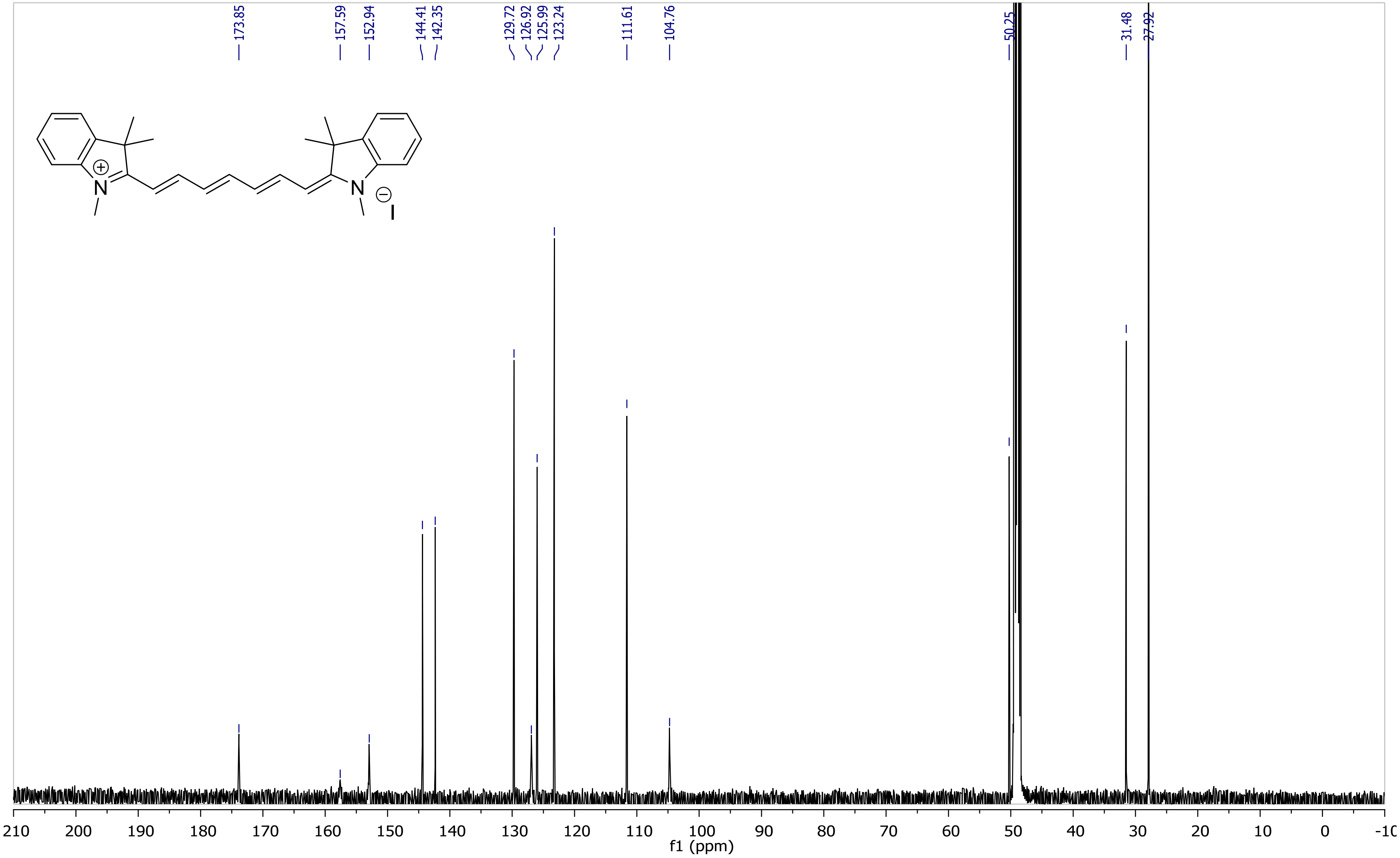

Figure S23. $^{13}C\{^{1}H\}$ NMR (125 MHz, CD_3OD): **1a**.

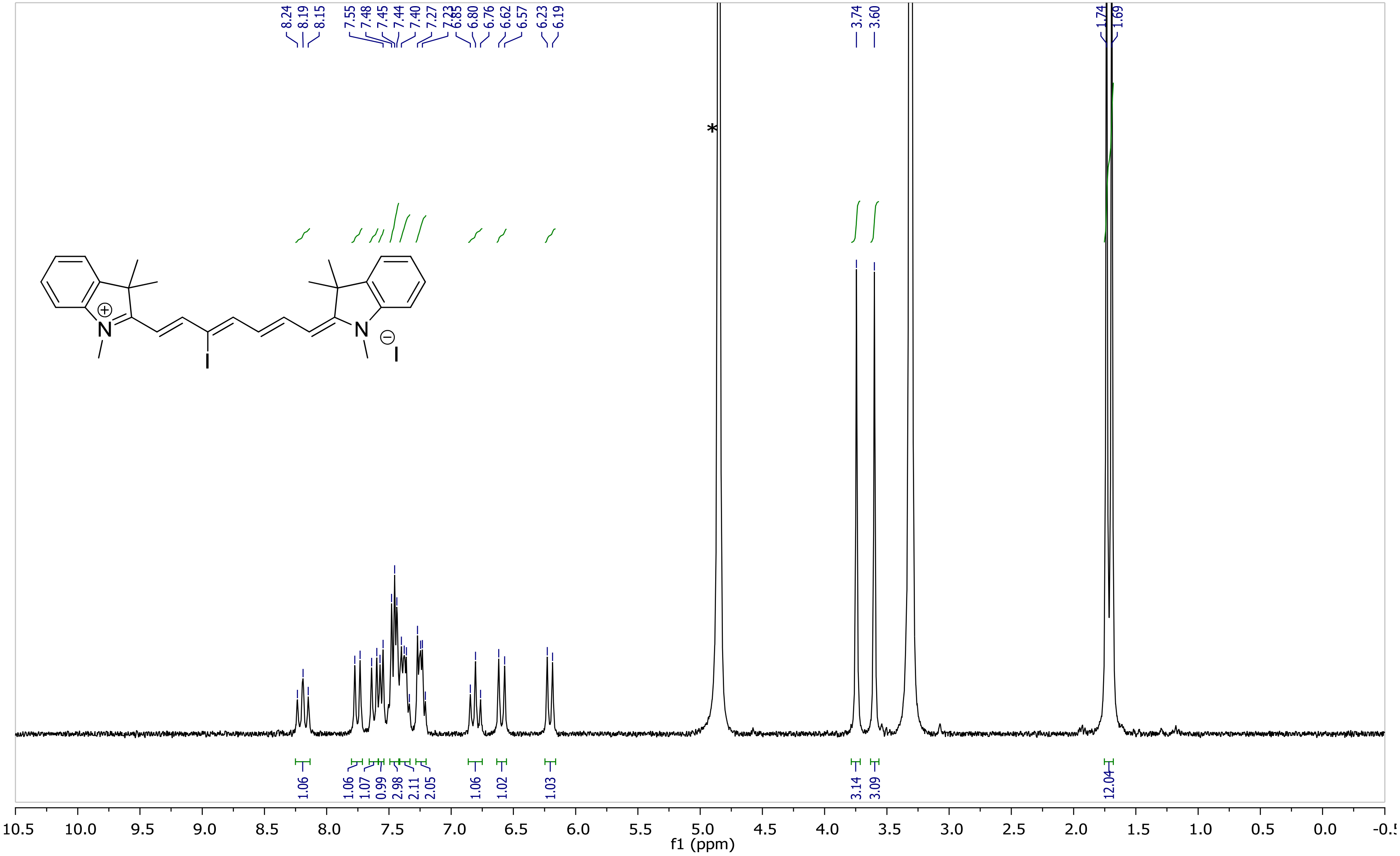

Figure S24. ^{1}H NMR (300 MHz, CD_3OD): **1b** (black asterisk denotes water).

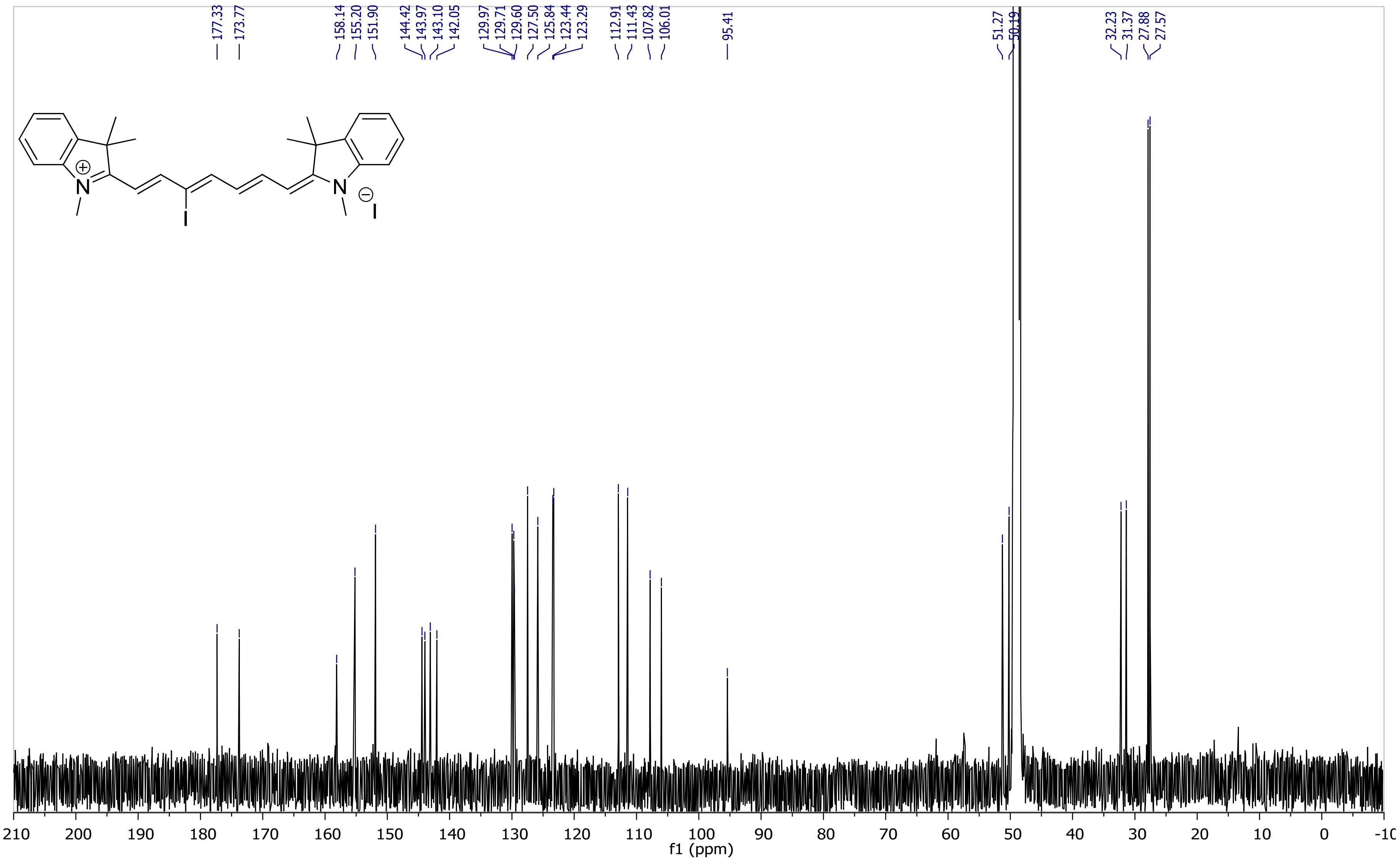

Figure S25. $^{13}C\{^{1}H\}$ NMR (125 MHz, CD_3OD): **1b**.

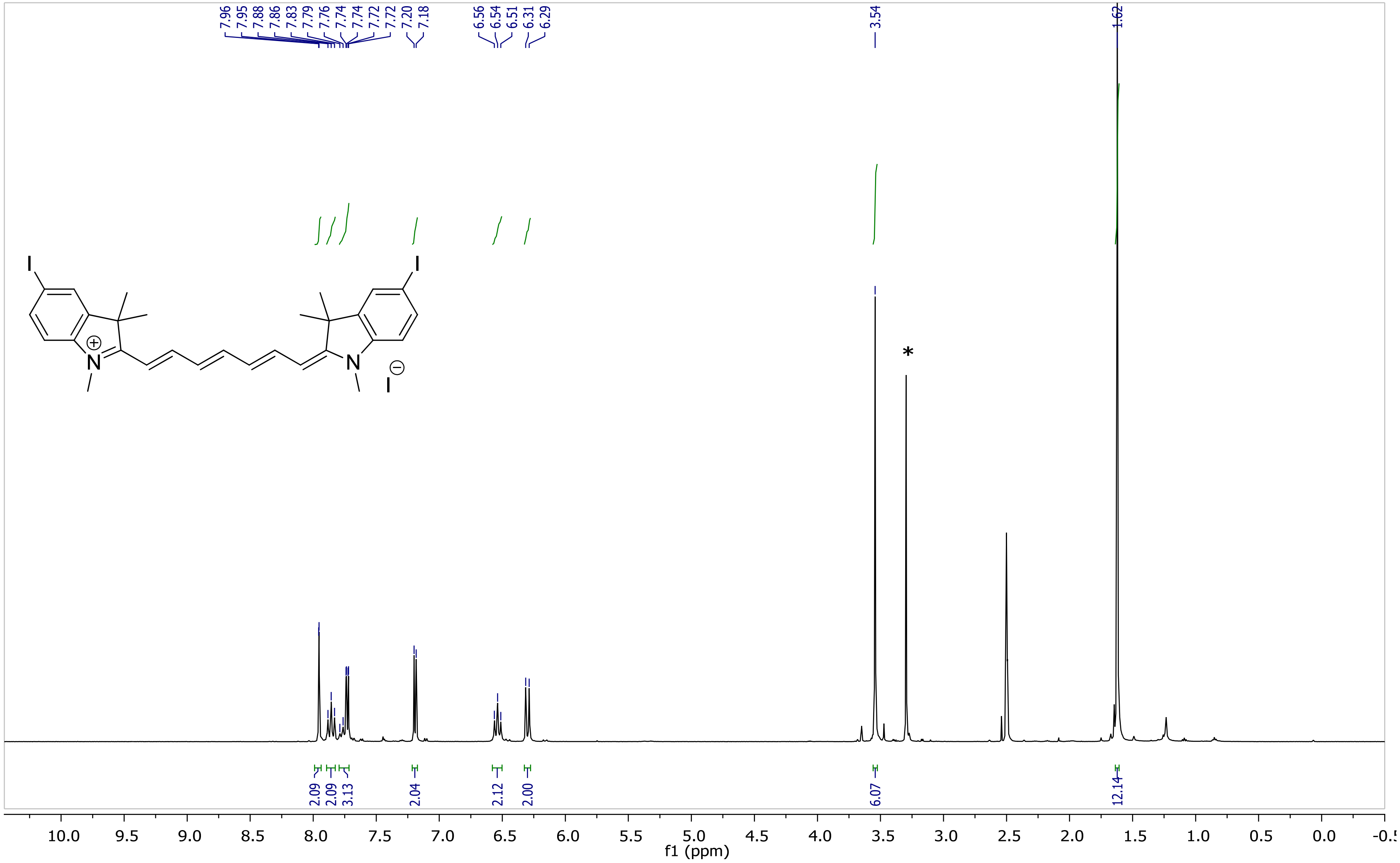

Figure S26. ^{1}H NMR (300 MHz, d_6-DMSO): **1c** (black asterisk denotes water).

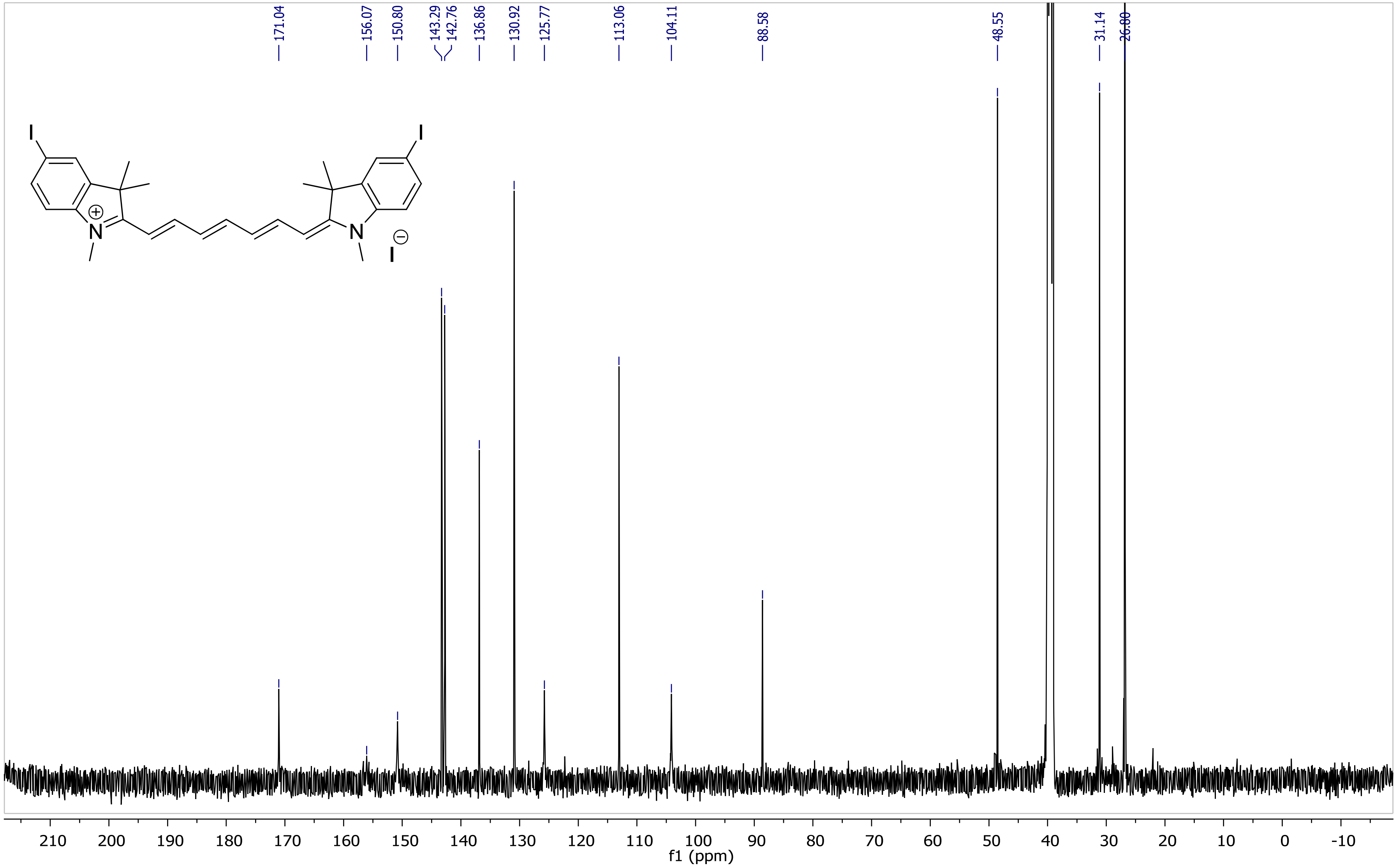

Figure S27. $^{13}C\{^1H\}$ NMR (125 MHz, *d*$_6$-DMSO): **1c**.

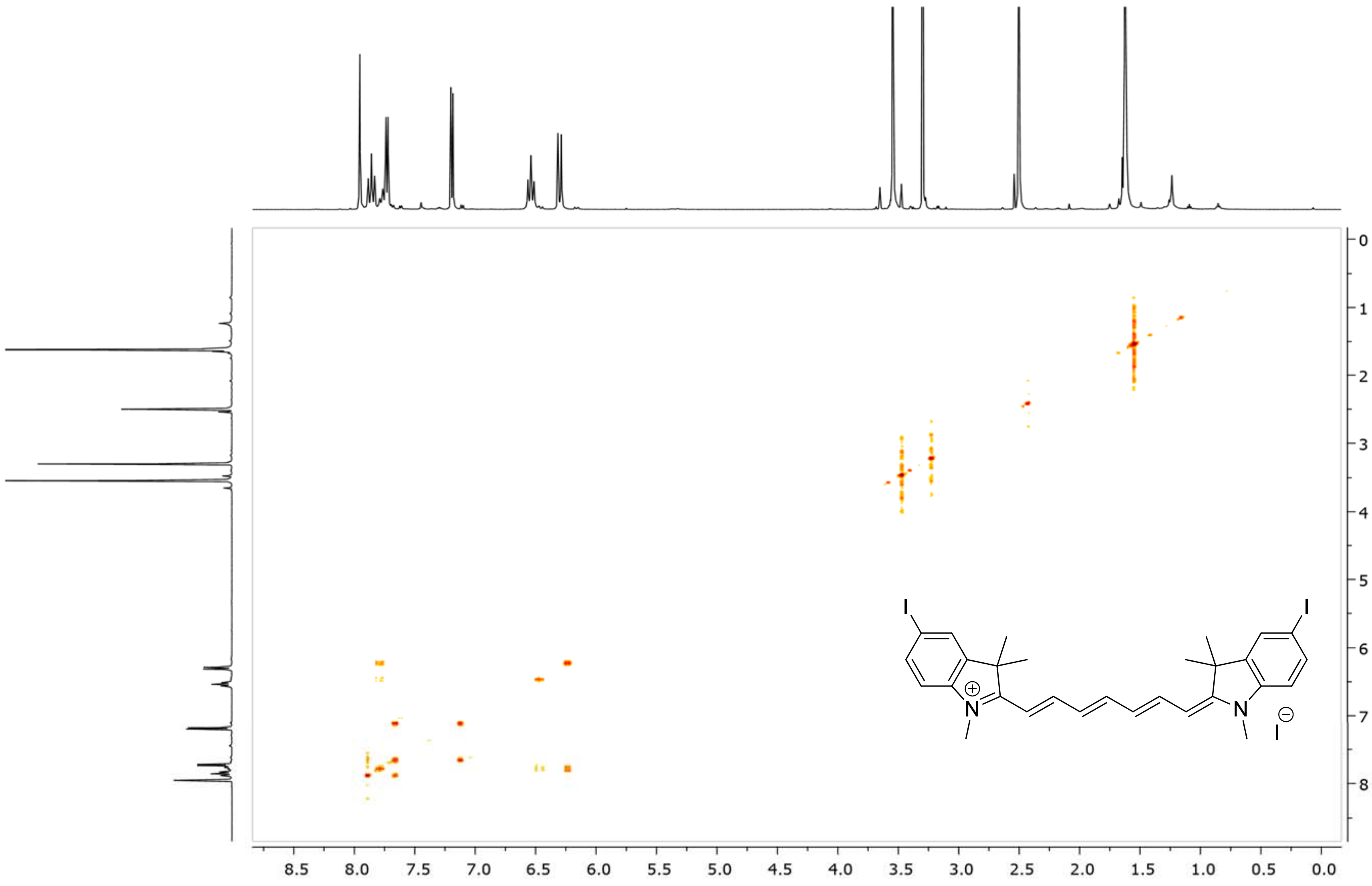

Figure S28. ^{1}H–^{1}H COSY NMR (500 MHz, d_6–DMSO): **1c**.

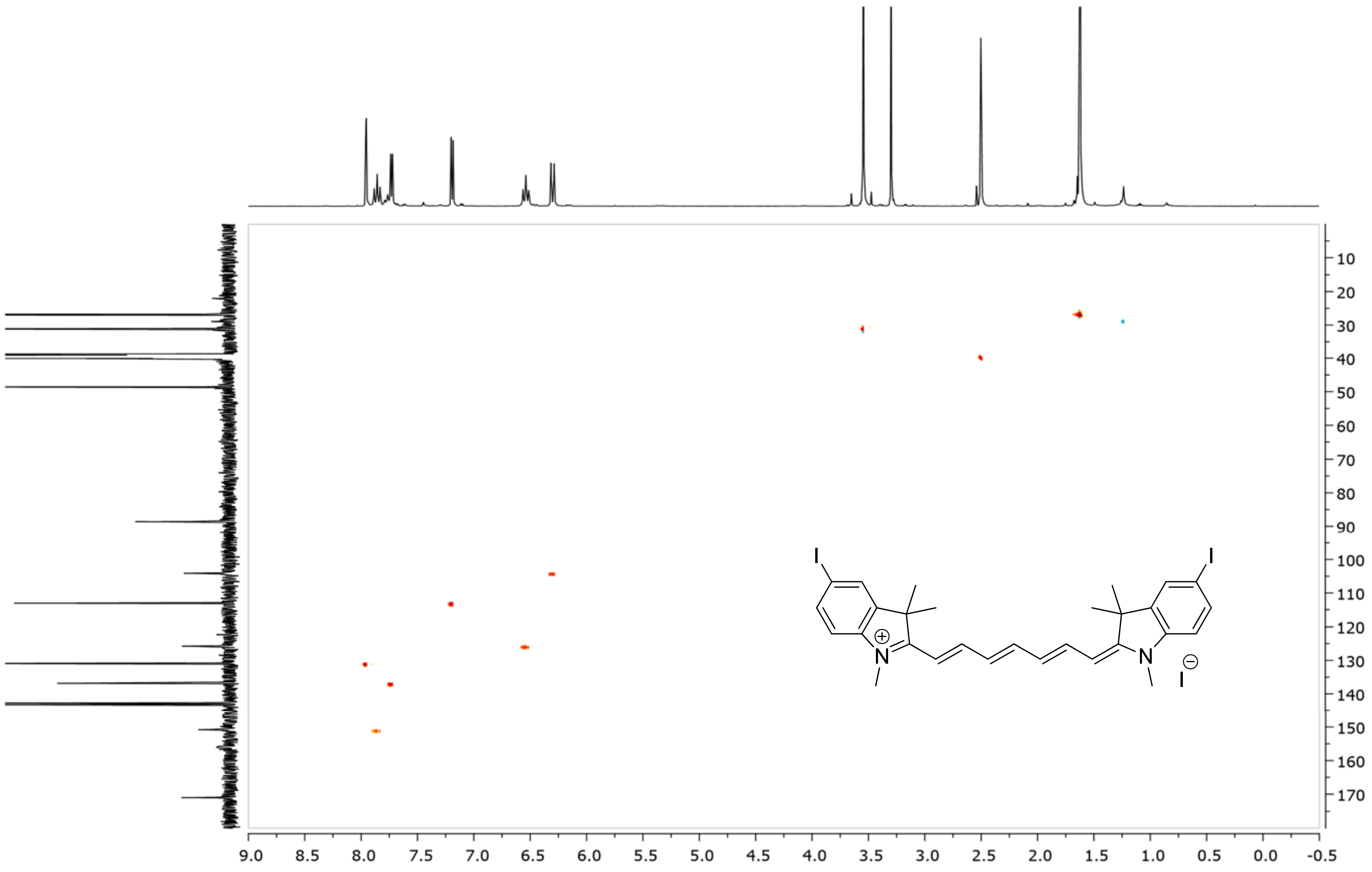

Figure S29. ^1H–^{13}C gHSQC (500 MHz, d_6-DMSO): **1c**.

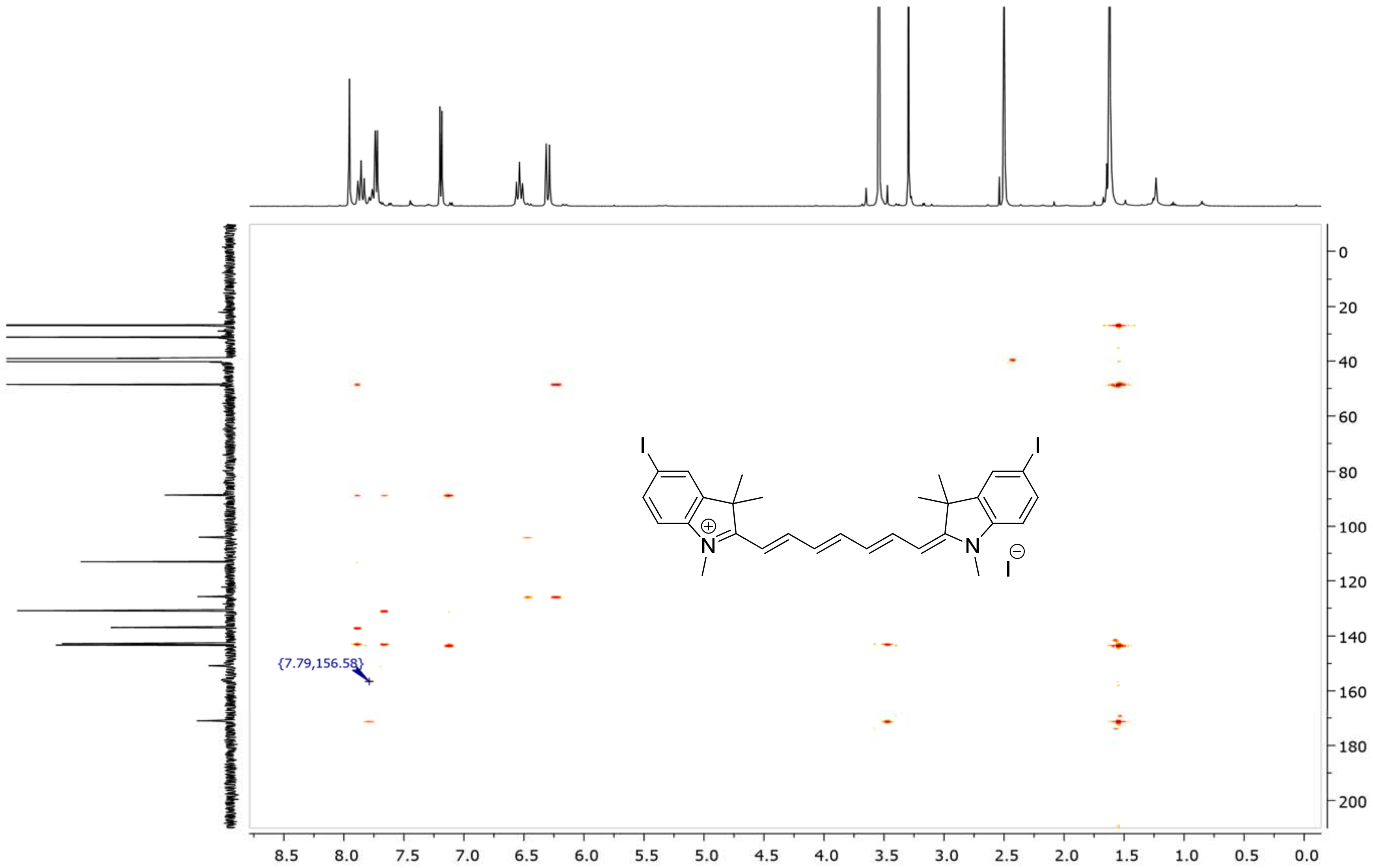

Figure S30. ^{1}H–^{13}C gHMBC (500 MHz, d_6-DMSO): **1c**.

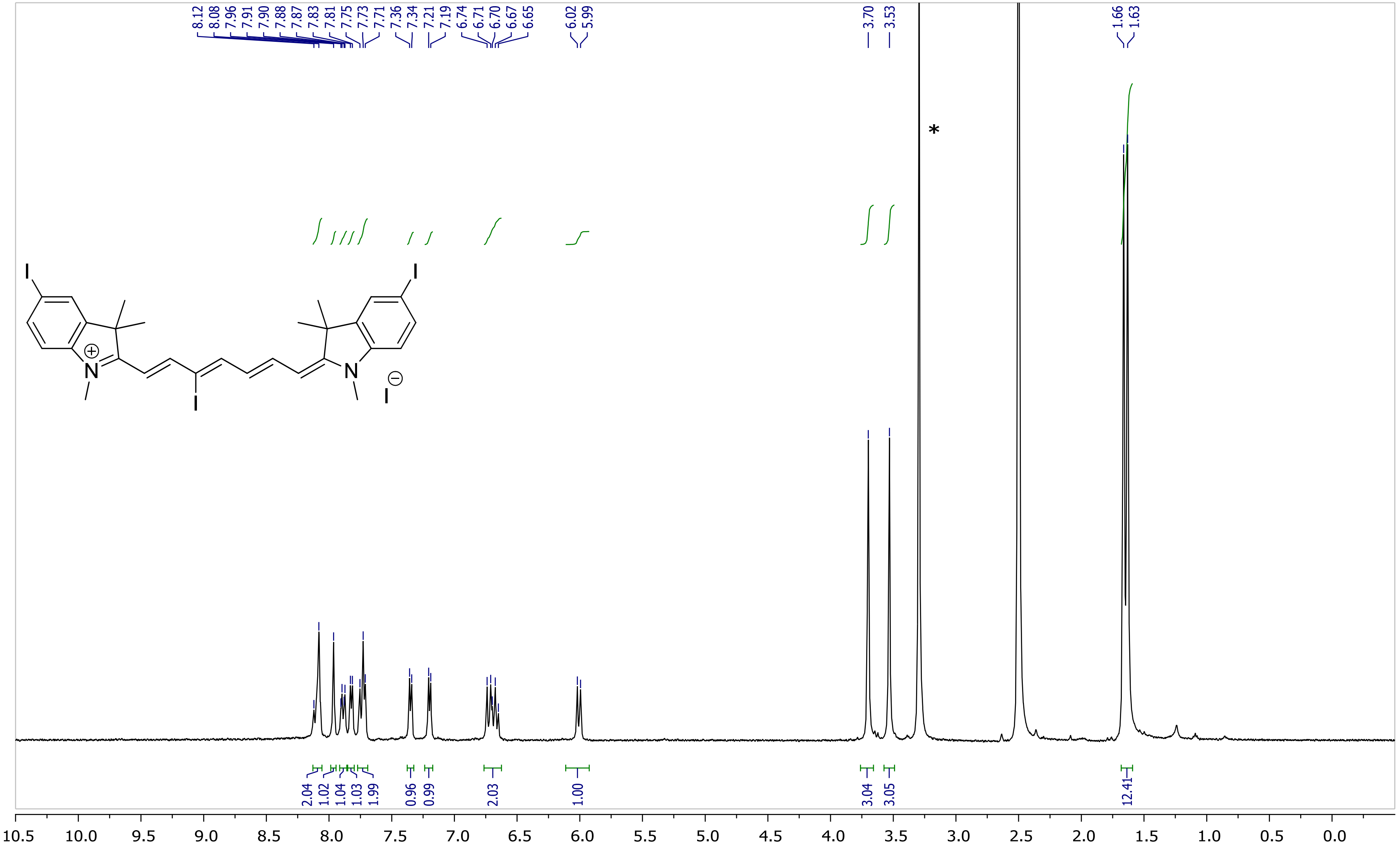

Figure S31. ^{1}H NMR (300 MHz, d_6-DMSO): **1d** (black asterisk denotes water).

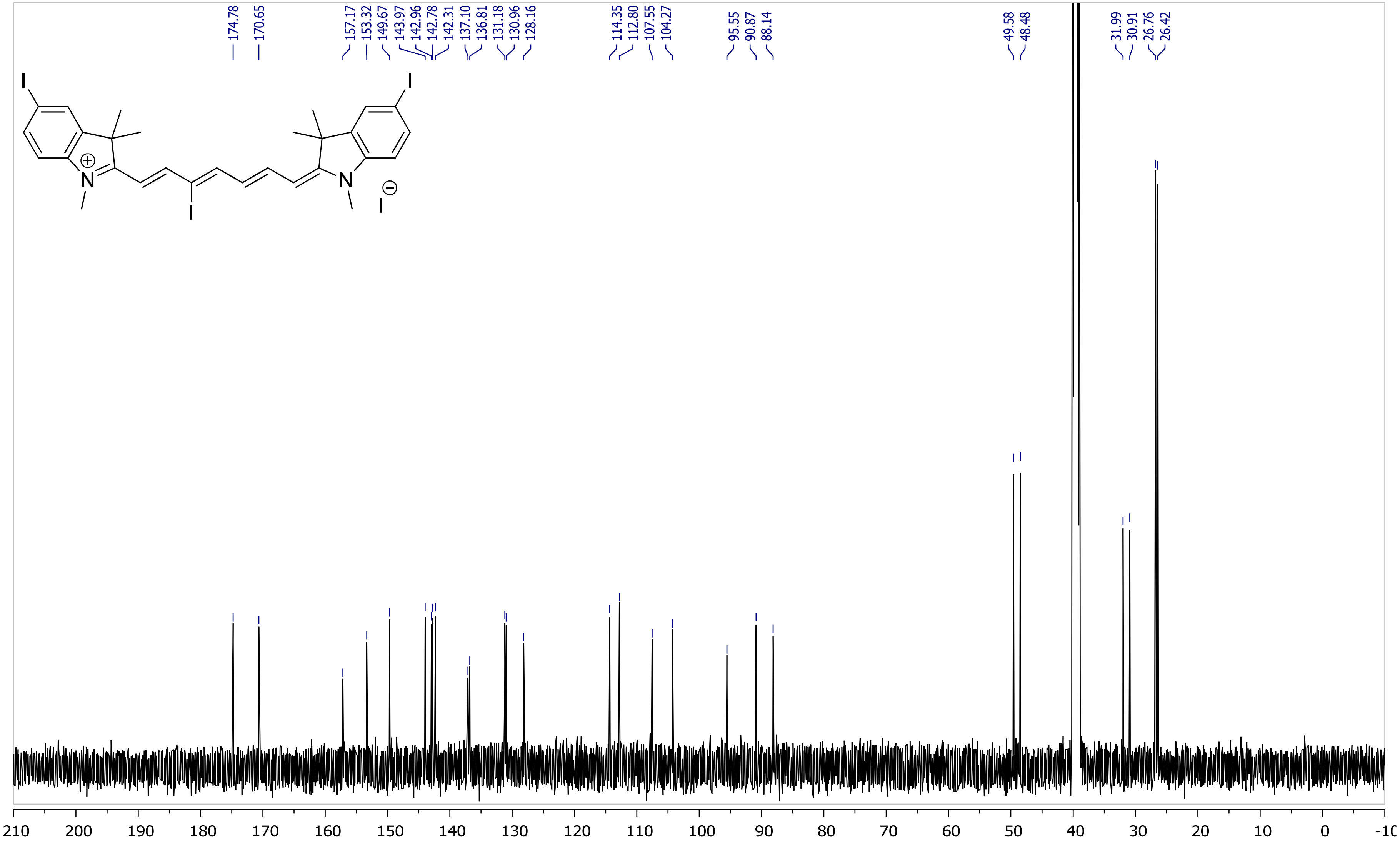

Figure S32. $^{13}C\{^{1}H\}$ NMR (125 MHz, d_6-DMSO): **1d**.

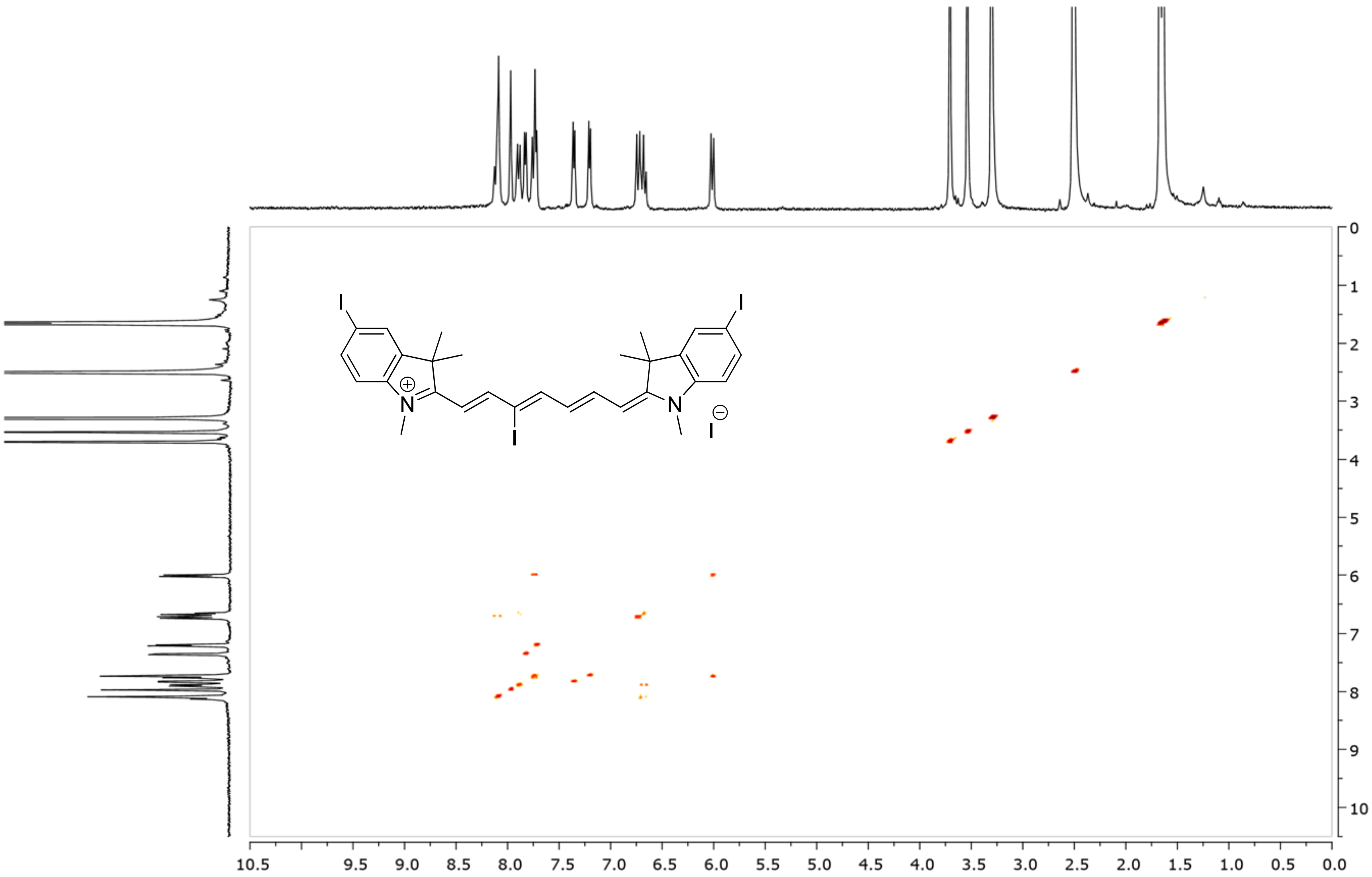

Figure S33. ^{1}H–^{1}H COSY NMR (500 MHz, d_6–DMSO): **1d**.

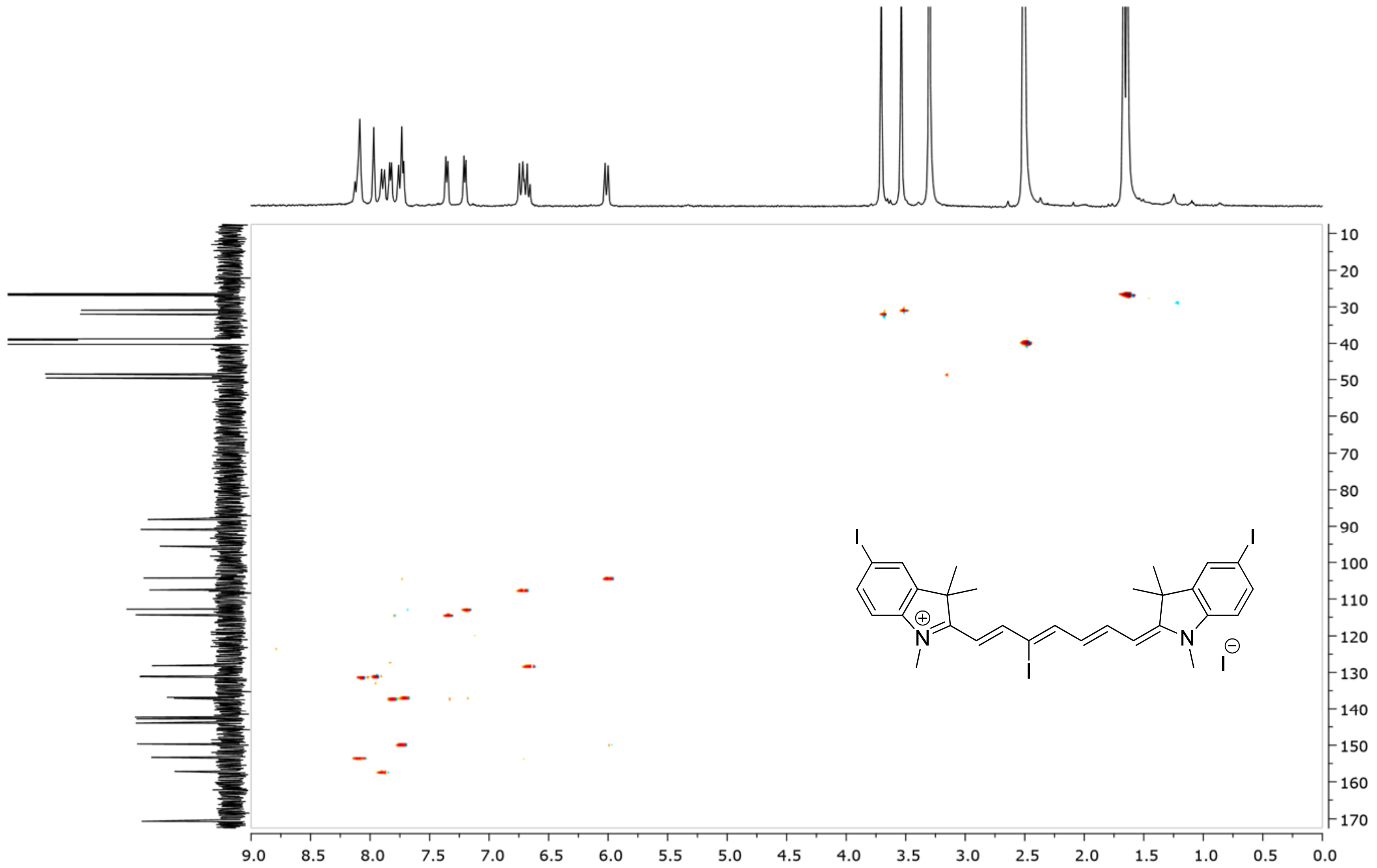

Figure S34. ^{1}H–^{13}C gHSQC (500 MHz, d_6-DMSO): **1d**.

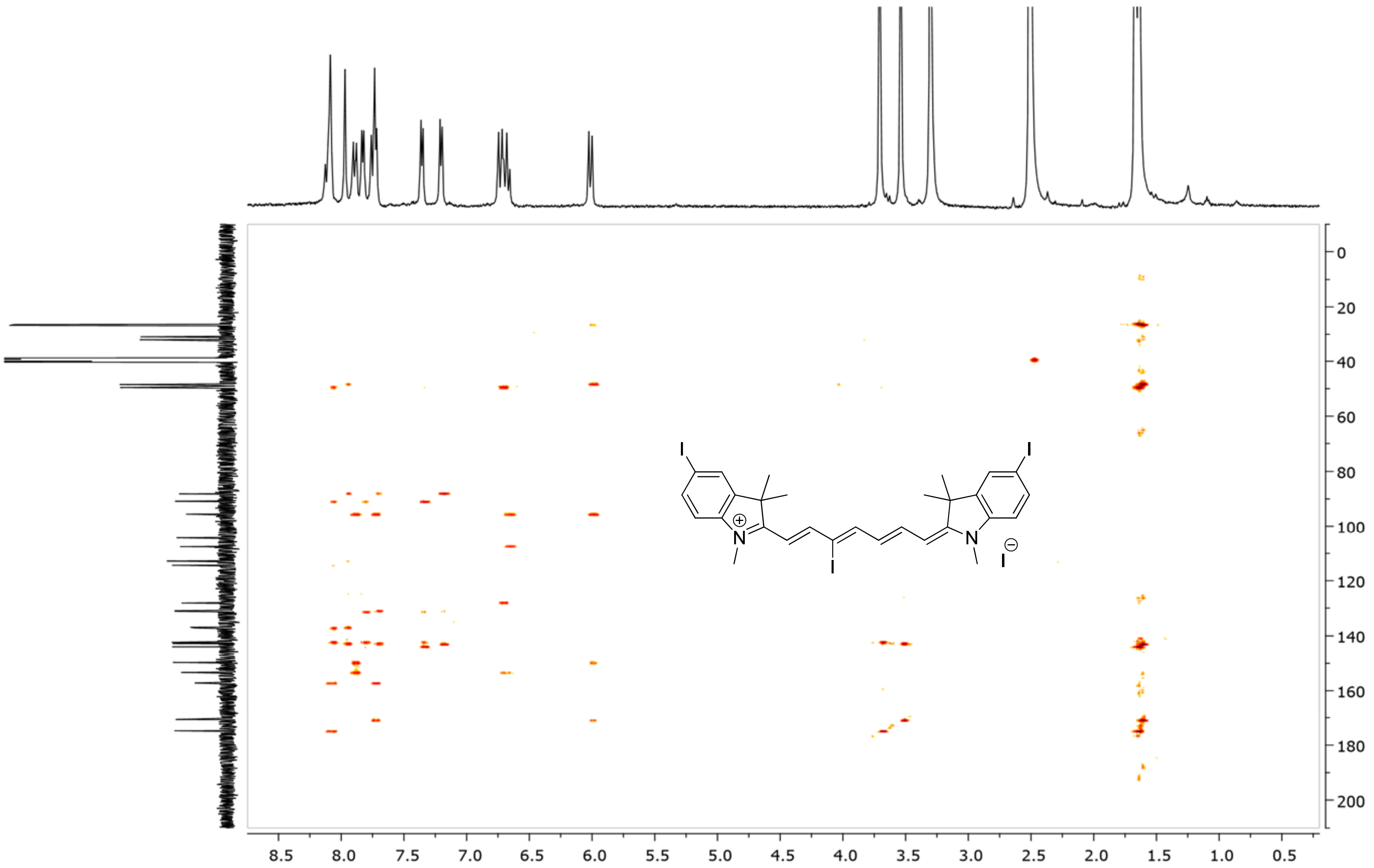

Figure S35. ^{1}H–^{13}C gHMBC (500 MHz, d_6-DMSO): **1d**.

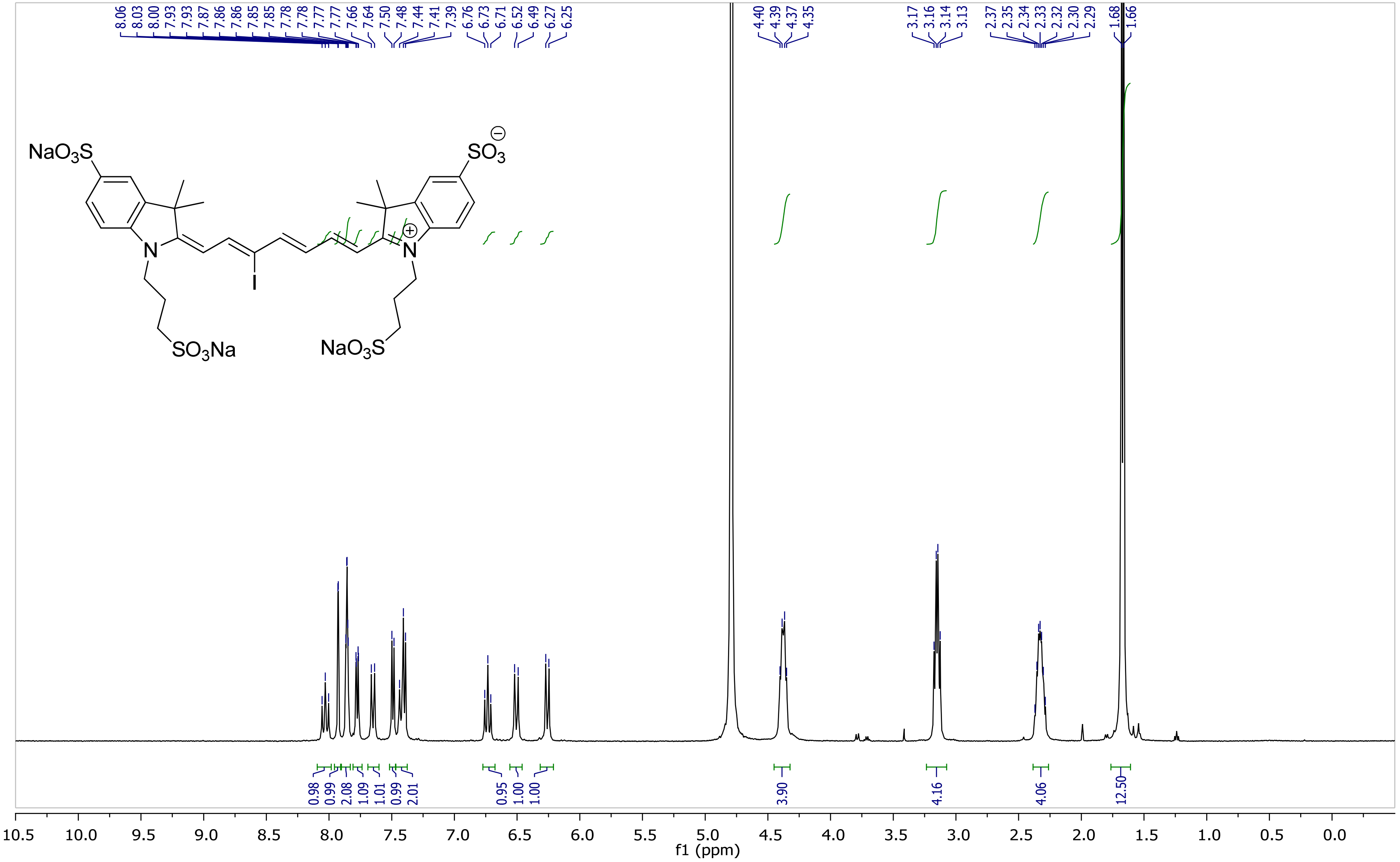

Figure S36. ^{1}H NMR (500 MHz, D_2O): **1e**.

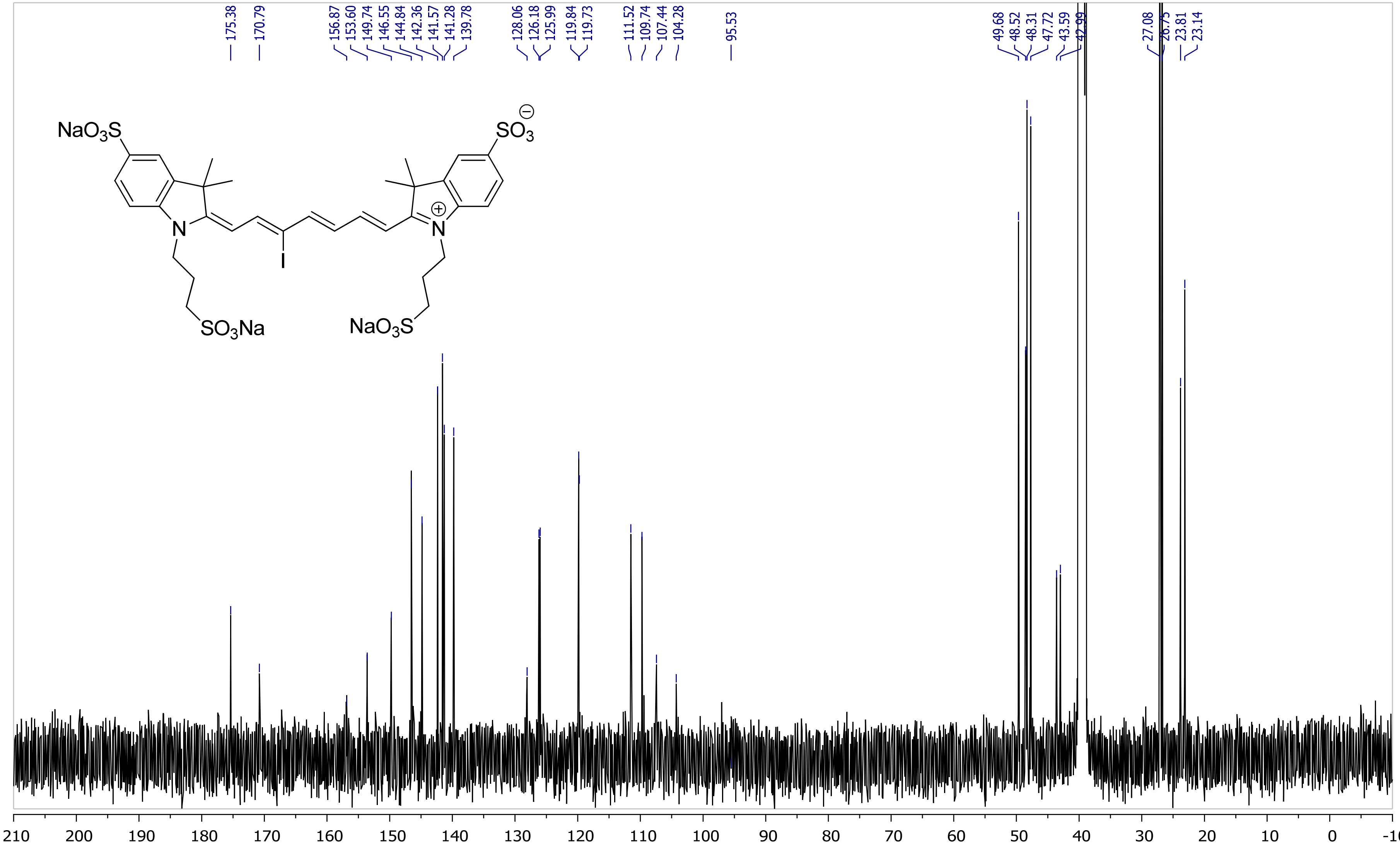

Figure S37. $^{13}C\{^{1}H\}$ NMR (125 MHz, d_6-DMSO): **1e**.

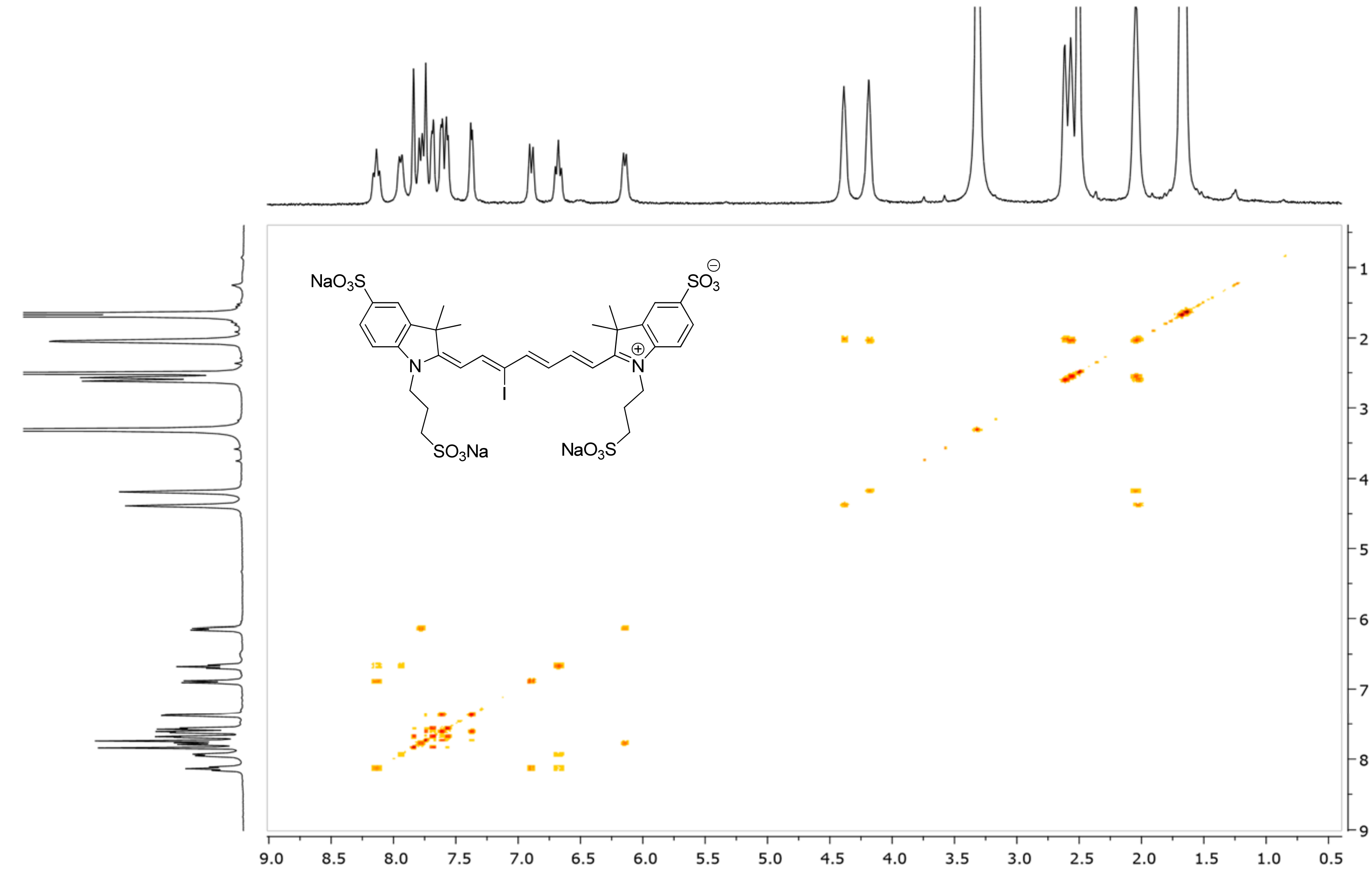

Figure S38. ^{1}H–^{1}H COSY NMR (500 MHz, d_6–DMSO): **1e**.

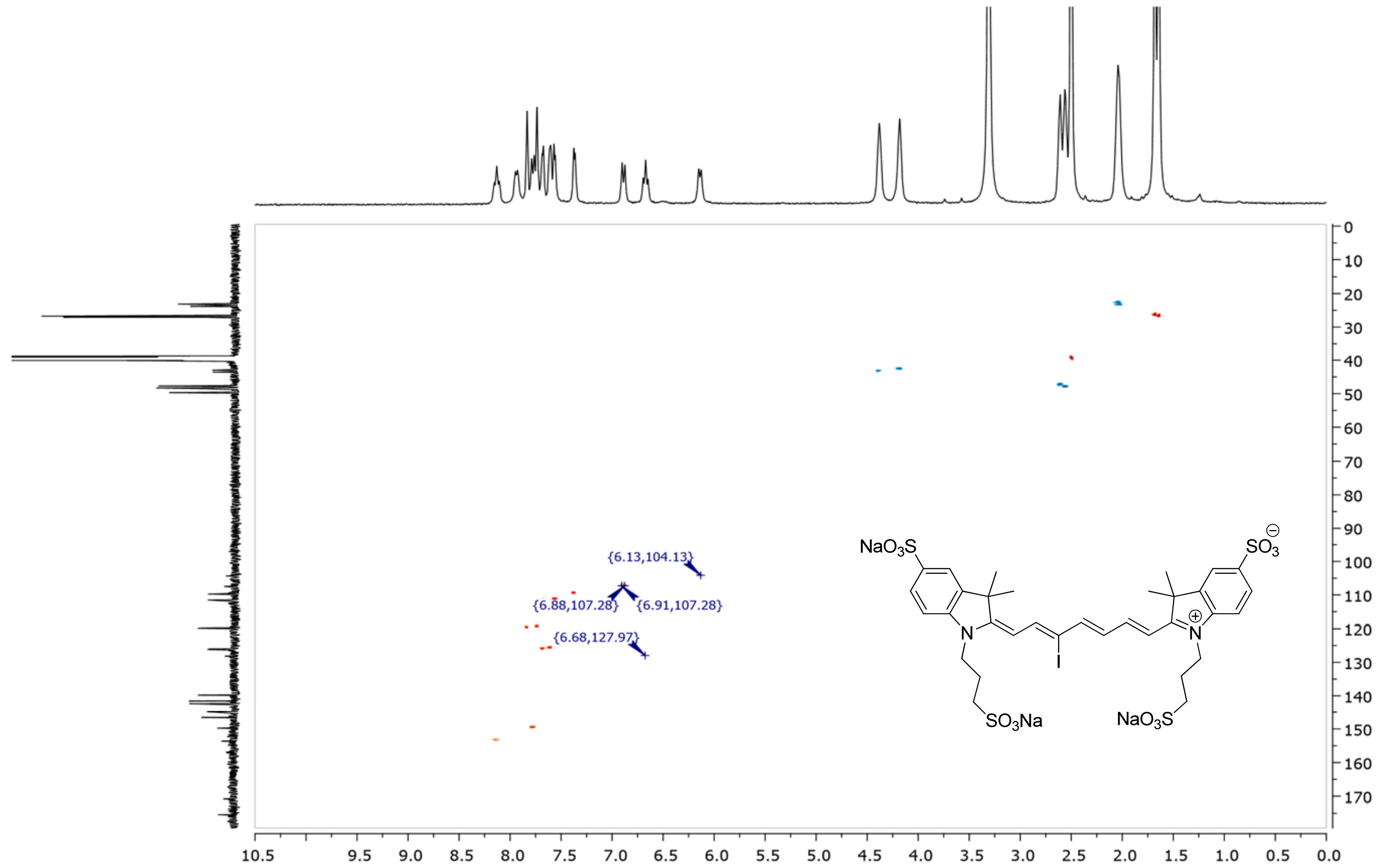

Figure S39. ^{1}H–^{13}C gHSQC (500 MHz, d_6-DMSO): **1e**.

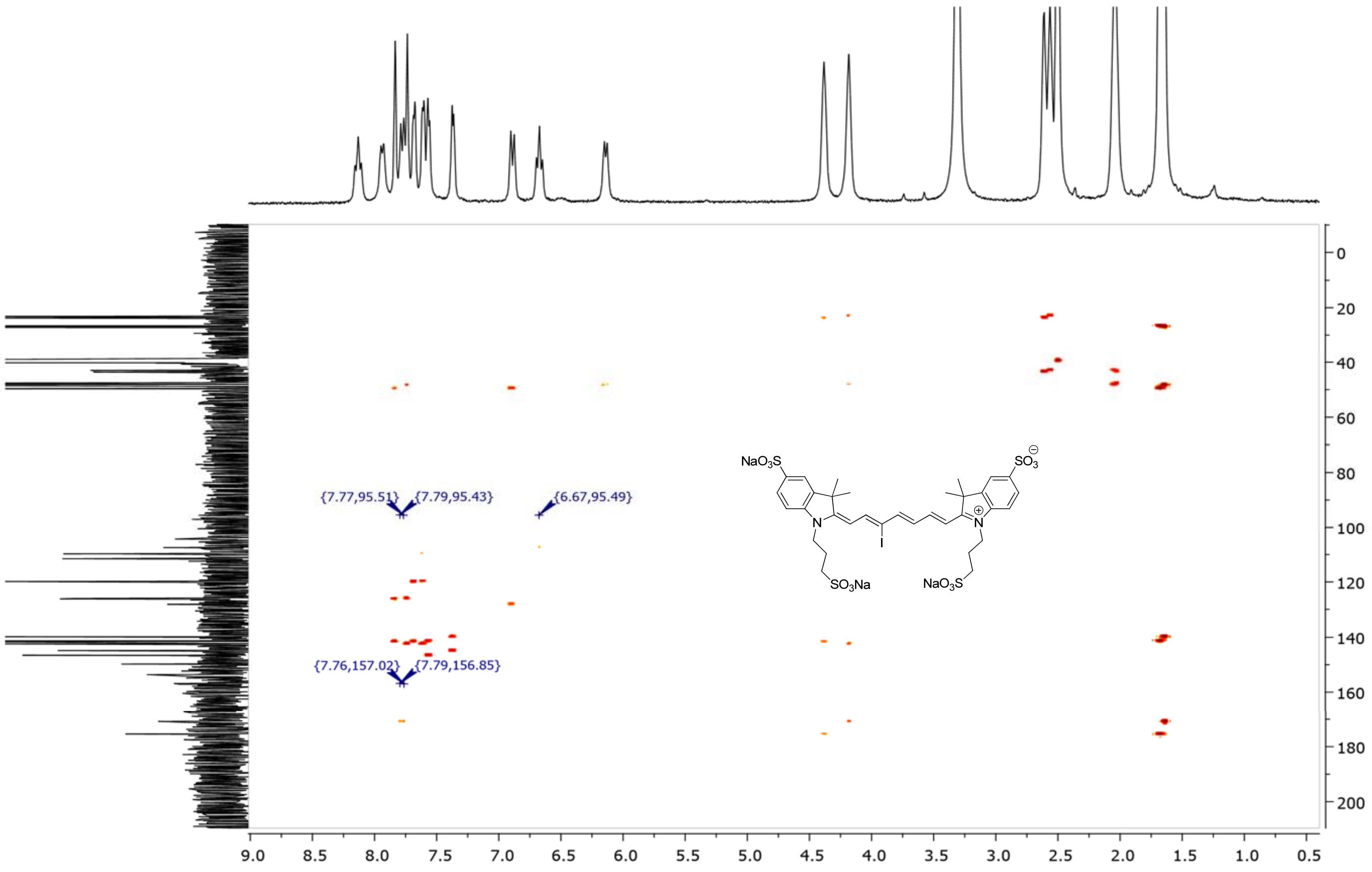

Figure S40. ^{1}H–^{13}C gHMBC (500 MHz, d_6-DMSO):**1e**.

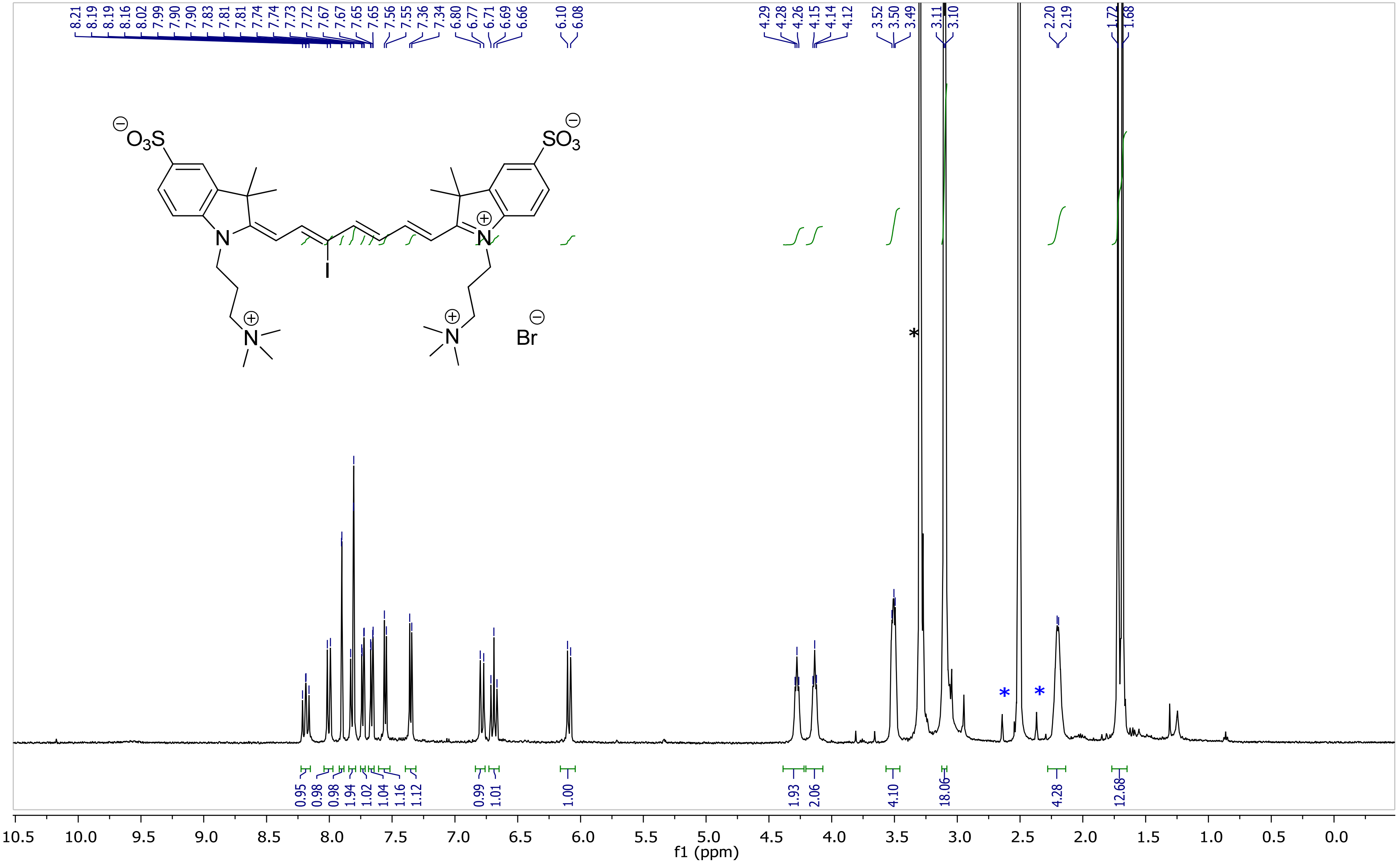

Figure S41. ^{1}H NMR (500 MHz, d_6-DMSO): **1f** (black and blue asterisks denote water and d_6-DMSO satellites, respectively).

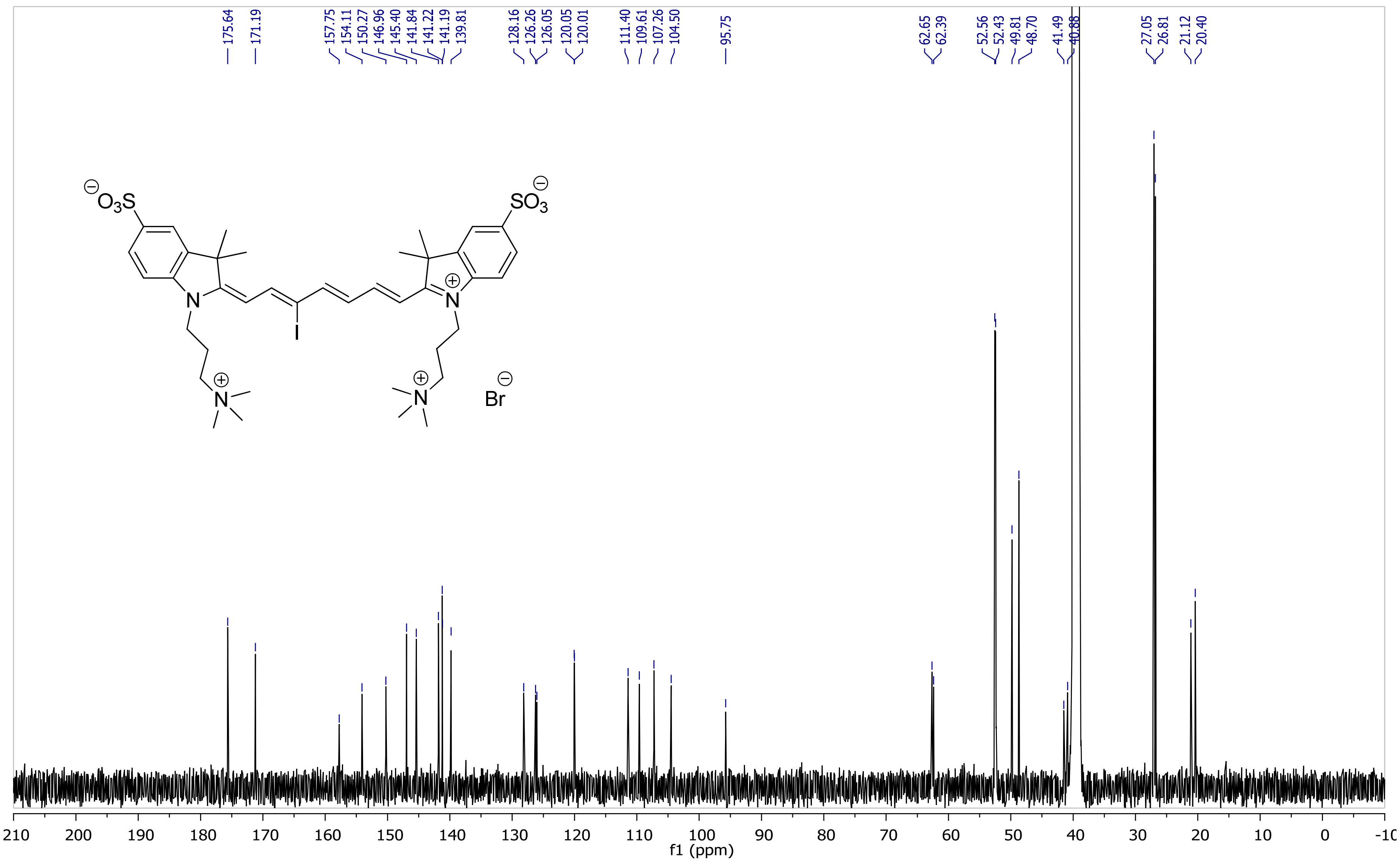

Figure S42. $^{13}C\{^{1}H\}$ NMR (125 MHz, d_6-DMSO): **1f**.

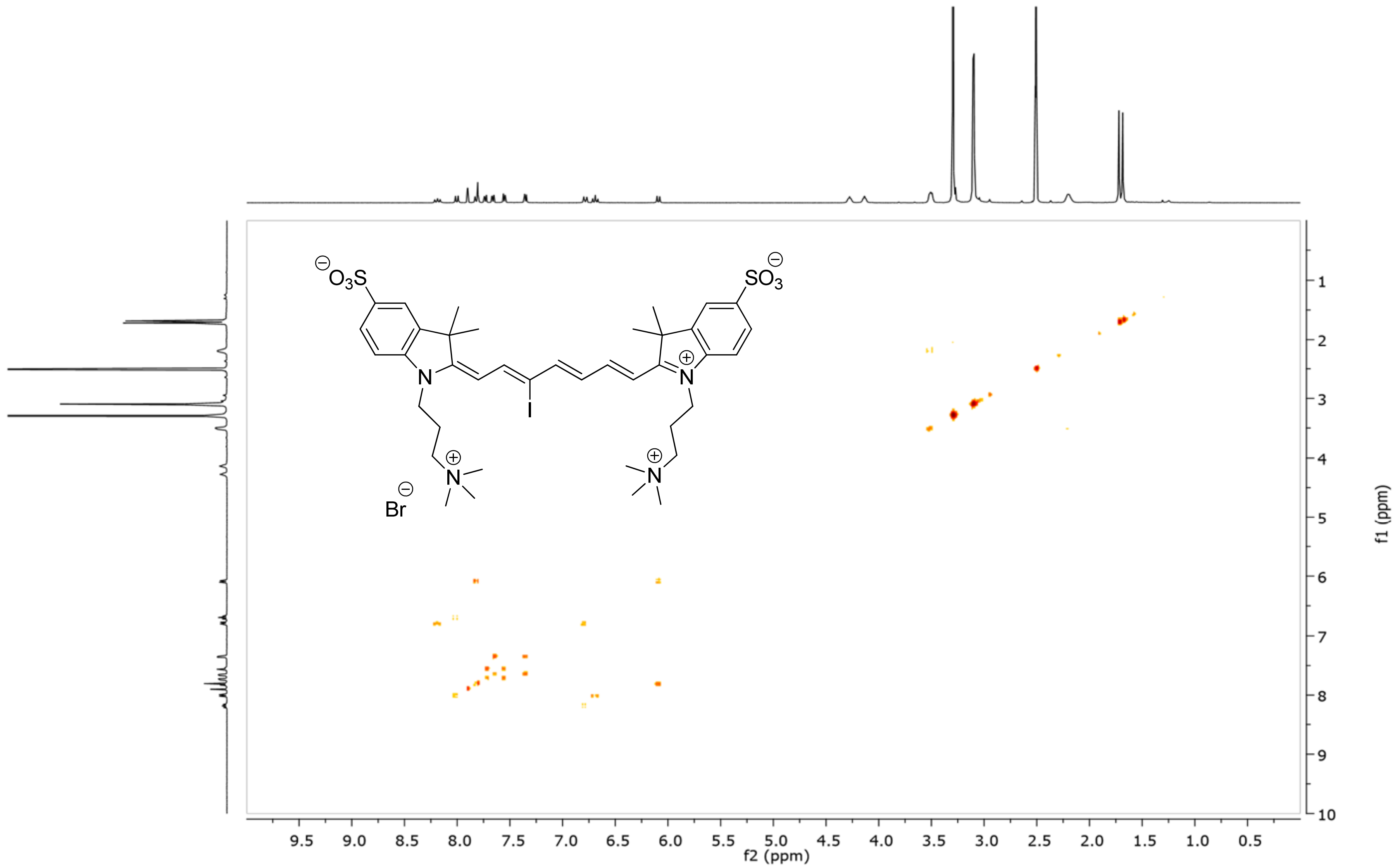

Figure S43. ^1H–^1H COSY NMR (500 MHz, d_6–DMSO): **1f**.

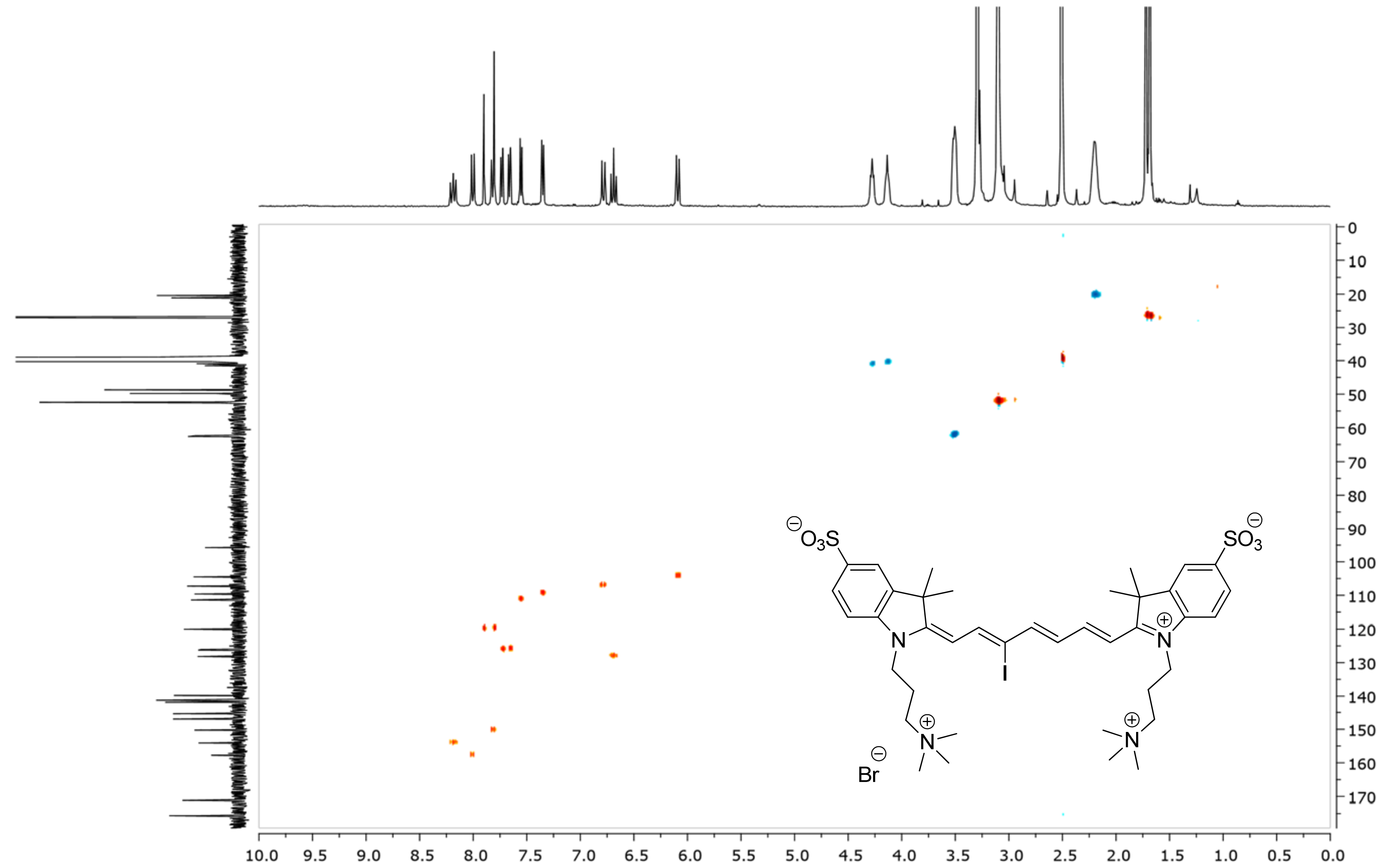

Figure S44. ^{1}H–^{13}C gHSQC (500 MHz, d_6–DMSO): **1f**.

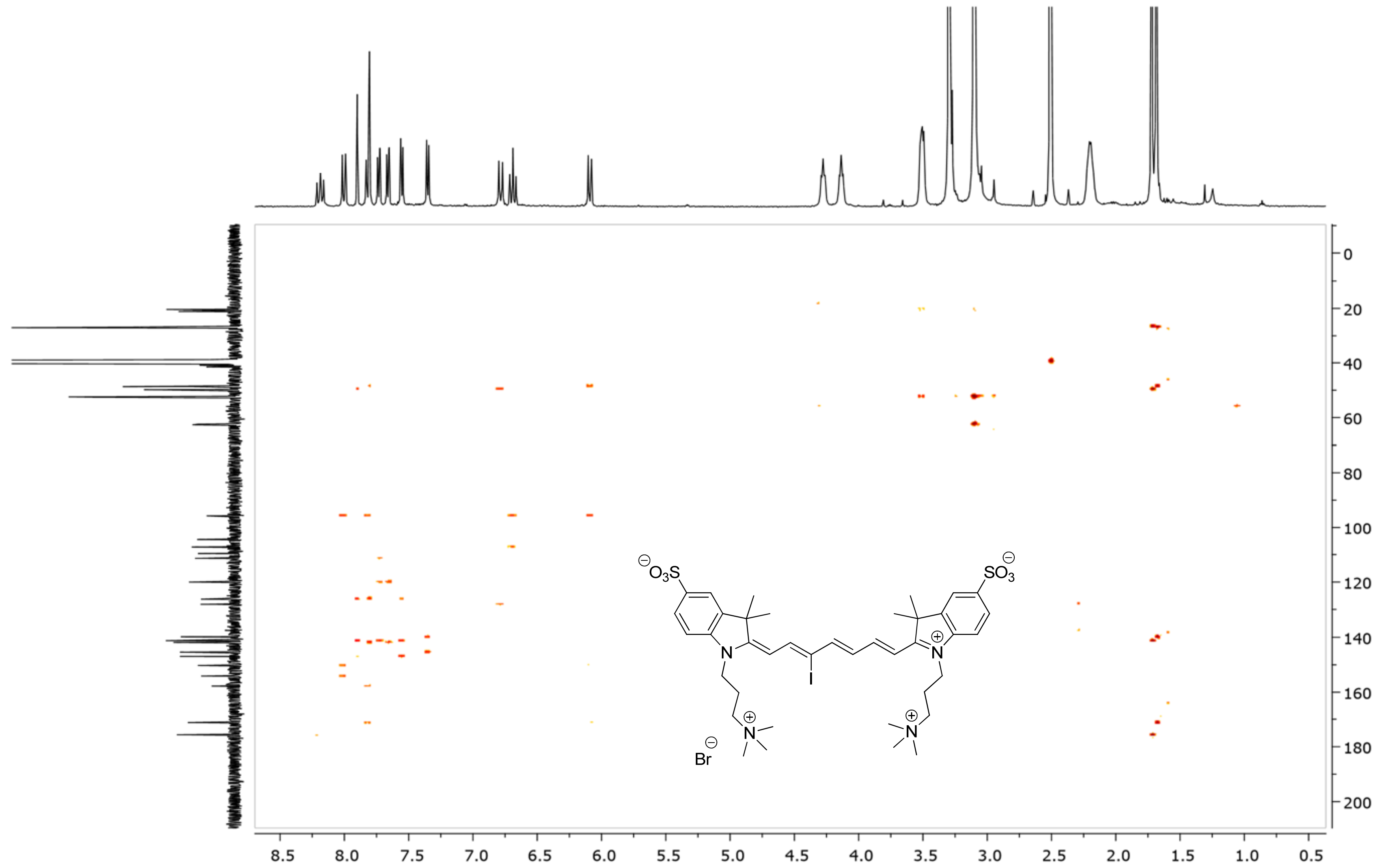

Figure S45. ^{1}H–^{13}C gHMBC (500 MHz, d_6–DMSO): **1f**.

Table S1. Optimized ground state geometries of **1a**, **1b**, **1c**, and **1d** calculated at CAM-B3LYP/def2-TZVP level in water modeled as polarizable continuum. The first number (64) is the number of atoms in the molecule. There are no imaginary frequencies.

64
molecule **1a**

C	0.59943332665212	0.01394968503640	16.23561045254111
C	-0.33159214422440	0.01004924048556	15.23414227740518
C	-1.67038851871755	0.00627455307113	15.55147022279222
C	-2.11711832776628	0.00635638724516	16.84816392073910
C	-1.16847411190619	0.01038890974235	17.85647500500204
C	0.17995477320525	0.01413522964546	17.55709851777701
C	-0.16976231229775	0.00884089505955	13.74625803062630
C	-1.61172575400120	0.00397438150207	13.29010005577955
N	-2.41375885621942	0.00272981176175	14.36332947141416
C	-2.11836206271711	0.00074081924759	12.01513095715962
C	-1.38800049209932	0.00200898920003	10.84038554714480
C	-1.95499449675501	-0.00159407310225	9.58588883973155
C	-1.19582790509703	0.00001986764946	8.43279369367202
C	-1.70044240237172	-0.00377589183147	7.14805769468462
C	-0.88358066824814	-0.00206394983376	6.03949955112465
C	-1.34794838994196	-0.00580887816881	4.73651538487045
C	-0.57976042116477	-0.00470628511415	3.59902317558408
C	0.92607085410769	0.00053484541185	3.46027067210596
C	1.08438819034428	-0.00083513241841	1.97163380778510
C	-0.15609577220140	-0.00628585620069	1.37696330267273
N	-1.13516574646318	-0.00855299101111	2.38046015776136
C	2.20690448189599	0.00230241121576	1.18994080004157
C	2.07622274099029	-0.00001621914612	-0.19041131118536
C	0.82202367303250	-0.00551301115535	-0.76881332434674
C	-0.31883189017085	-0.00876038935954	0.01548763207523
C	1.54426887340899	1.26996120249840	4.05809780656051
C	1.55306452486444	-1.26303078377126	4.06138633626978
C	-2.55836522643786	-0.01402435731423	2.12711017396560
C	-3.85818362804859	-0.00179797991572	14.30760840126921
C	0.57069576243235	-1.25613708186152	13.29514624821458
C	0.56341911247294	1.27704294667025	13.29236112522091
H	-2.77697017031283	-0.00833926007644	7.00595201995372
H	-0.30586652576875	0.00612272822007	10.88177090812989
H	-3.03761091094967	-0.00596749978057	9.50087410302152
H	-0.11384316059331	0.00444419858668	8.54555202014799
H	0.18308691597491	0.00245311113185	6.22874336910199
H	-2.42245942467901	-0.01005319988105	4.60783380421564
H	-2.73086410559839	-0.01793191318518	1.05733822461373
H	-3.01749811886289	-0.90230267943097	2.56021597378499
H	-3.02342524122081	0.87381716278700	2.55482636705116
H	1.08654057703299	2.16066938344703	3.62838376715050
H	1.42450960561091	1.30665382019849	5.13876874441080
H	2.61058743788565	1.29014603381786	3.83412997724863
H	1.43256555790129	-1.29824361733304	5.14202290902807
H	1.10217988302981	-2.15798631074463	3.63326253954943
H	2.61969812261079	-1.27587450130964	3.83833295469223
H	-1.29334082864312	-0.01308497324420	-0.44880233092309
H	0.72493307022685	-0.00734403353510	-1.84628936399062
H	2.95774516272713	0.00249587572044	-0.81623799007709
H	3.19408832574280	0.00661329208032	1.63232358606884
H	-4.21453364691040	-0.89184553846168	13.78966694576400
H	-4.25587224960136	-0.00111548797394	15.31570057151118
H	-4.21991704938865	0.88412831191169	13.78635457598969
H	1.65819198603845	0.01692822163936	16.01190031636332
H	0.90888747436780	0.01724768793703	18.35556480678848
H	-1.49152462878031	0.01052451794479	18.88869910711890
H	-3.16775573693788	0.00341542516878	17.09570115140080
H	0.03874975932366	-2.15034864843258	13.61960298855762
H	0.68404640681914	-1.29404478215982	12.21394078559974
H	1.56476384685433	-1.26797885685408	13.74084031827080

H	0.67706238355929	1.31302483939496	12.21113318653832
H	0.02611395907603	2.16886855391519	13.61450647011419
H	1.55726108319959	1.29573143192878	13.73835357498708
H	-3.19585494629108	-0.00312958866576	11.91121899135812

64

molecule **1b**

C	6.39946562689748	1.17128543851428	0.10623678044562
C	7.35829460696578	0.19762612851055	0.04862178962763
C	6.97972343541128	-1.12167312412509	-0.03027266553482
C	5.66602669752582	-1.51232186329694	-0.05490186322988
C	4.70121943366651	-0.52159004146863	0.00264818341261
C	5.06154420157367	0.80994509589421	0.08260008387090
N	8.13703465899709	-1.91680287047398	-0.07599287120673
C	9.23691323530520	-1.16749038834738	-0.03413305731906
C	8.85076258097039	0.28998949844816	0.05343990132676
C	10.49526242086675	-1.73312044167966	-0.07241562421493
C	11.68588283410666	-1.05347079613659	-0.02961367150325
C	12.92813906154933	-1.66952740768916	-0.07293914003063
C	14.08003560175110	-0.93459696055819	-0.02776820270855
C	15.40470762058313	-1.34933953268655	-0.06063327318512
I	15.85323520252056	-3.36361746630684	-0.18687281733503
C	9.33869333982000	0.92684125782397	1.36086058550589
C	9.33748286488809	1.07755367728483	-1.16962566307782
C	8.12444420209338	-3.36203114389503	-0.15936936490004
C	16.40988372391194	-0.41412064859192	-0.00625640001102
C	17.77289263247264	-0.61126403304200	-0.02392026853384
C	18.72464265356698	0.37782919552127	0.02775645261328
N	20.02939262715599	0.08680289204251	0.00662017454516
C	20.80935128064007	1.25089577196620	0.07131702080625
C	19.97105110157754	2.33919220701705	0.13371498320531
C	18.54737865521852	1.87638268821709	0.11015470958346
C	20.50314290963394	3.59744976495732	0.20304435268477
C	21.88088488559662	3.75380671385625	0.20907438506063
C	22.70653573300493	2.64830444439314	0.14585640942367
C	22.17485040626324	1.37173591608345	0.07587088038451
C	20.56907733204324	-1.25188447834098	-0.07411718611295
C	17.83218559010546	2.43166879143243	-1.12761778095372
C	17.83433089845424	2.29629749343175	1.40099692569188
H	11.68778545677319	0.02665024473287	0.04277176503557
H	12.97009006059630	-2.74968551544986	-0.14522254277318
H	13.96201436945963	0.14395767490385	0.04362912727549
H	16.03434928693376	0.59806023820307	0.05840115581220
H	18.12325045791144	-1.63015447865412	-0.08384919965346
H	21.65138563100370	-1.20067881391930	-0.10324681468424
H	20.26704329155409	-1.83655084740474	0.79479059447786
H	20.21687752589101	-1.74644179291604	-0.97875215682319
H	18.33933108085152	2.11623259824939	-2.03915803480287
H	16.79594116883278	2.10551876072722	-1.17630922099335
H	17.84242035292786	3.52044149884237	-1.08640141079909
H	16.79688919215685	1.97107845372421	1.41394266707599
H	18.34049885258905	1.88234339268142	2.27275585368926
H	17.84848024368040	3.38301942257780	1.47856732790493
H	22.82880869291128	0.51461383248048	0.02711958823333
H	23.78068052514914	2.77679670748884	0.15073224934905
H	22.31159535446736	4.74394592887992	0.26318939816837
H	19.86548729049554	4.46956028982385	0.25249784822953
H	8.64904561117987	-3.79175496373432	0.69266974672711
H	7.09851340146521	-3.71019836739450	-0.15225438173873
H	8.60368746179966	-3.69055421138626	-1.08068882274986
H	6.66969212217956	2.21697732531426	0.16851852480184
H	4.29594018636515	1.57206171068397	0.12720585447174
H	3.65548385133006	-0.79692119889420	-0.01544568989518
H	5.37396884210038	-2.54926832368982	-0.11754264845548
H	8.99327890429764	0.35323177164396	2.22061578733233
H	10.42382669863737	0.99047454418617	1.39907564706950
H	8.93676750308502	1.93653468255328	1.43760818371086

H	10.42269953053912	1.14573962278717	-1.20008083901186
H	8.99253726934466	0.60906700028190	-2.09115119318744
H	8.93452066805060	2.08868948334844	-1.12629073516004
H	10.55107806030269	-2.81149144942675	-0.14318539696794

64
Molecule **1c**

C	-0.41311350999597	-0.10502999155960	0.00351623840882
C	-0.21817956210229	-0.04830722895538	1.35729310281887
C	1.04033362291680	-0.06917733110212	1.90509502224098
C	2.13152630591339	-0.14756215611145	1.09043154944602
C	1.97768291175059	-0.20737999223921	-0.28196493682218
C	0.70472121759300	-0.18477910781097	-0.80564132578273
C	0.94300712928326	0.00152127781353	3.39591125869034
C	-0.55604939746581	0.05460550242264	3.58944132793761
N	-1.15787621572296	0.03163167317242	2.38844410355524
C	-1.28084864403136	0.10925095751062	4.75001216771967
C	-0.77866392536822	0.13076958700676	6.04131903678387
C	-1.57570746794656	0.14909325220978	7.16255475225254
H	-2.65407770392445	0.14773758237830	7.03531340267546
C	-2.58862220749337	0.07330419480614	2.18576043557006
C	1.62561695582343	1.27480509786523	3.90995706535494
C	1.55275201303270	-1.25601668650043	4.02576522926559
C	-1.06017588979958	0.14885339646971	8.44447024243349
C	-1.82314859434695	0.12514844419001	9.59458958000904
C	-1.27272892048169	0.10666661993717	10.85677299933899
C	-2.03417715678676	0.04562913198511	12.01136863673830
C	-1.57430444981041	0.01561635356194	13.30181580020061
N	-2.42162448388825	-0.06994242921681	14.33985360488698
C	-1.72972829375983	-0.08525891296579	15.55404953703003
C	-0.38344176167378	-0.00329643047753	15.29798315428620
C	-0.15454009318529	0.06707684111675	13.82114963046191
C	-2.21447427999279	-0.16556214838598	16.83144652888991
C	-1.30150333155732	-0.16047902902244	17.86872957074896
C	0.05350791622952	-0.07753754760198	17.64048451670257
C	0.50265241001177	0.00102427942974	16.33510435668774
C	-3.86086945746087	-0.13981069412414	14.22269986195108
C	0.66540690852285	-1.14209925125023	13.35516469551796
C	0.53406494692574	1.38587461917101	13.45115958109869
H	-0.19170669834969	0.13433297565823	10.91988124409328
H	-2.90480839913453	0.10678386676809	9.50055214465212
H	0.02179137033783	0.15759217800877	8.55478249438438
H	0.29167548803434	0.12595153216799	6.20614104375856
H	-2.35893537845503	0.12833455690485	4.65731780013930
H	-2.80097506213501	0.06046601372271	1.12278213303522
H	-3.06369135697768	-0.79053307438440	2.65056331157670
H	-3.00491425873576	0.98407284065228	2.61545432017558
H	1.17971494882046	2.15944805860450	3.45554850288813
H	1.54776427018223	1.36263368499808	4.99139329275218
H	2.68210918350681	1.24563052343925	3.64456988907203
H	1.48100924941567	-1.23660773165757	5.11069306999636
H	1.04962585641768	-2.15081037203703	3.65930298986848
H	2.60643242454373	-1.31671756843278	3.75449452766118
H	-1.39559430734450	-0.09089760285082	-0.44110387510885
H	0.55748679703479	-0.23058564816634	-1.87366158463758
I	3.59660141203445	-0.32733371283230	-1.50871057837191
H	3.12331927338069	-0.16451556721587	1.51502450465627
H	-4.15147282232624	-1.01766319776460	13.64637931271919
H	-4.29697108026760	-0.21060866667365	15.21249992005309
H	-4.24493976790674	0.75370640251258	13.73134450498999
H	1.56207349892365	0.06544691517570	16.13998120682247
I	1.36274048763297	-0.07256669861007	19.19773815215214
H	-1.67848730148182	-0.22387945827076	18.87789416517500
H	-3.26948769804292	-0.23133367619961	17.04628168812522
H	0.15778793663065	-2.07229851162918	13.61004320384156
H	0.83639371475376	-1.12166499482536	12.28125839851297
H	1.63410707070289	-1.12955401969764	13.85400053812119

H	0.68975918978600	1.46868273106892	12.37778038027415
H	-0.06102750524060	2.23614680055949	13.78423554190386
H	1.50613411046876	1.43081563890735	13.94155606630482
H	-3.10655363741771	0.01013290837641	11.87153696331420

64

molecule **1d**

C	0.86160755424622	0.01327054307691	15.90312575869585
C	-0.16551323721873	0.00970067322803	15.00500053734879
C	-1.46026041051437	0.00564036924061	15.45752051220711
C	-1.75976160794064	0.00552412422719	16.79208320509163
C	-0.70633091068382	0.00933277026257	17.68593859940280
C	0.60361721046758	0.01315454471929	17.26034328696691
C	-0.15360574475544	0.00898245080176	13.51034311588844
C	-1.63409696201098	0.00447968649532	13.20851254970455
N	-2.32186228272454	0.00224967412398	14.35258463329662
C	-2.27608537119613	0.00278424394426	11.98843451047199
C	-1.67211086002580	0.00356187083269	10.75419673517062
C	-2.37388314668052	0.00246519044679	9.55739112069086
C	-1.72069437597908	0.00177350514167	8.35487701208976
C	-2.23429180509614	0.00216062055622	7.06378668819683
I	-4.28026618446904	0.00404968888044	6.76836397277811
C	-3.76630172483657	-0.00283596973886	14.44978756750229
C	0.53744256109452	-1.25749108951174	12.98886492956368
C	0.53009676191693	1.27895333734352	12.98765078151052
I	2.12586939460201	0.01887310498653	18.60958772140084
C	-1.37388167687250	0.00047849929504	5.99221545463083
C	-1.67208912304015	0.00175569522838	4.64592024218835
C	-0.74841911243779	-0.00144860162563	3.63062955334936
C	0.76092051825853	-0.00921976011148	3.71060848670882
C	1.12670605870181	-0.01001090776492	2.26123820746833
C	-0.01285955845800	-0.00343534647533	1.49578410019605
N	-1.12519162815468	0.00160377924256	2.34467487847667
C	2.34809800588767	-0.01609027650004	1.65448178706010
C	2.44507579966368	-0.01558209933834	0.27581835300279
C	1.28695376264083	-0.00887824125156	-0.46808898122715
C	0.04050040203473	-0.00268361607791	0.12855485396809
I	4.26110602904484	-0.02548892112325	-0.64114042461571
C	1.30293902345954	1.25511883677959	4.38833379910039
C	1.28953111962459	-1.27991494806309	4.38678201576088
C	-2.49889143654328	0.00943820014579	1.89227624453318
H	-0.59189394564555	0.00439217462237	10.67889345746504
H	-3.45665080542582	0.00196601521246	9.59216218004033
H	-0.63396817001406	0.00078070674587	8.39396962001401
H	-0.33518099887444	-0.00226399045264	6.29308825007402
H	-2.71466391733699	0.00516856950488	4.36660379398324
H	-2.51959605152518	0.01279901121176	0.80851578410902
H	-3.01965060031802	-0.87724000349977	2.25274258250194
H	-3.01109353435811	0.89893263862551	2.25794738299697
H	0.90671119218318	2.14902567676602	3.90686067949754
H	1.04880892323611	1.28694111923952	5.44521092263960
H	2.38893584418355	1.26741639103140	4.29948743107001
H	1.03381239882442	-1.31079285969782	5.44334494976106
H	0.88481500428050	-2.16903742861130	3.90351059158620
H	2.37545044624191	-1.30299395539765	4.29905253139852
H	-0.84655453751288	0.00239374112257	-0.48474696971416
H	1.33538603951029	-0.00863402018908	-1.54615451171864
H	3.24761039343119	-0.02165853876365	2.25058668279242
H	-4.17265647688753	-0.89104362121520	13.96778495667013
H	-4.05306060298706	-0.00726601716617	15.49481034602051
H	-4.17875935643297	0.88559978032082	13.97336993222202
H	1.88320745844000	0.01641497437368	15.55599213684114
H	-0.93395724075427	0.00900491942387	18.74076405076631
H	-2.77415258237057	0.00243767604603	17.15827132612218
H	0.03348841337800	-2.15020005304342	13.35901517557144
H	0.55145466358152	-1.29042224248242	11.90197760271405
H	1.56759980564438	-1.27044777039038	13.34333719496094

H	0.54452200890425	1.31061091826691	11.90072442189650
H	0.02055633593603	2.16909204646662	13.35638451096815
H	1.55996926294732	1.29861270691085	13.34266029716409
H	-3.35806541228442	0.00064180360133	11.99987388100535

Table S2. Optimized ground state geometries of **1a**, **1b**, **1c**, and **1d** calculated at CAM-B3LYP/def2-SVP level in the gas phase. The first number (64) is the number of atoms in the molecule. There are no imaginary frequencies.

64

molecule **1a**

C	0.659528	0.011337	16.271733
C	-0.308412	0.008132	15.281145
C	-1.657597	0.004786	15.626070
C	-2.082599	0.004862	16.947555
C	-1.099339	0.008122	17.938508
C	0.255308	0.011270	17.609782
C	-0.175261	0.007610	13.771199
C	-1.646611	0.003735	13.341586
N	-2.426599	0.001617	14.440871
C	-2.182219	0.002379	12.056670
C	-1.460012	0.003228	10.859726
C	-2.032402	0.001670	9.592886
C	-1.268177	0.002498	8.427024
C	-1.769720	0.000789	7.126409
C	-0.942346	0.001584	6.009017
C	-1.394785	-0.000129	4.686058
C	-0.599728	-0.000015	3.543139
C	0.929223	-0.001023	3.433314
C	1.117633	-0.004281	1.929241
C	-0.128374	-0.004090	1.307586
N	-1.130211	-0.000173	2.304162
C	2.272558	-0.007271	1.164530
C	2.159218	-0.010372	-0.228498
C	0.904470	-0.010627	-0.835399
C	-0.265550	-0.007582	-0.073717
C	1.542561	1.273376	4.043008
C	1.541422	-1.273345	4.048329
C	-2.551504	0.001481	2.025589
C	-3.874572	-0.002810	14.412938
C	0.556121	-1.264355	13.302724
C	0.550161	1.282475	13.301398
H	-2.850955	-0.001363	6.981748
H	-0.371769	0.005130	10.901467
H	-3.119846	-0.000398	9.505717
H	-0.180504	0.004596	8.542963
H	0.129987	0.003754	6.200866
H	-2.473445	-0.002404	4.544435
H	-2.711412	0.017453	0.947710
H	-3.028999	-0.897419	2.433151
H	-3.030635	0.887362	2.458462
H	1.093390	2.173561	3.611060
H	1.412361	1.313274	5.128666
H	2.616910	1.299058	3.830976
H	1.411275	-1.308552	5.134164
H	1.091398	-2.174937	3.620222
H	2.615729	-1.300925	3.836334
H	-1.236155	-0.008486	-0.562627
H	0.830451	-0.013456	-1.920909
H	3.056092	-0.012833	-0.843575
H	3.255678	-0.007341	1.632884
H	-4.249575	-0.892845	13.894326
H	-4.258910	-0.011836	15.432539
H	-4.255929	0.891945	13.907071
H	1.719400	0.013933	16.020524

H	1.002272	0.013793	18.400251
H	-1.400319	0.008278	18.983890
H	-3.134435	0.002684	17.221097
H	0.028596	-2.166443	13.629614
H	0.658251	-1.302464	12.214077
H	1.561400	-1.287520	13.736374
H	0.652328	1.319859	12.212752
H	0.018345	2.182424	13.627212
H	1.555272	1.310879	13.735146
H	-3.266690	0.000465	11.966202

64
molecule **1b**

C	6.25012159018823	1.13888034921594	0.10486871077870
C	7.23255680277237	0.16418387182300	0.04605016724122
C	6.87564325048820	-1.17865019535301	-0.04485487469294
C	5.55109073107706	-1.59213886439162	-0.08124872759610
C	4.56847681819347	-0.60224490111128	-0.02172925979922
C	4.90892888470028	0.74668504679291	0.07048456205476
N	8.05502373362847	-1.95673723774847	-0.08882929882114
C	9.15852593486228	-1.18917482398269	-0.03476823231345
C	8.74336992452691	0.28273305277171	0.06216074790851
C	10.44078072223734	-1.73715915042546	-0.06915175302178
C	11.64149626888333	-1.02997150387859	-0.01905233164108
C	12.90319488562759	-1.62214364690884	-0.05873717165983
C	14.06736737215463	-0.86637875580377	-0.01111922691914
C	15.40205047654519	-1.28877483731707	-0.04079894619765
I	15.84674919890055	-3.34248587552047	-0.16412891623199
C	9.21329498948182	0.91712849382231	1.38448516937848
C	9.22655727993195	1.09001425841909	-1.15698691045253
C	8.06893364130651	-3.40279221047673	-0.18344509473148
C	16.43382151471792	-0.35034679488436	0.00936435805970
C	17.80747752244055	-0.57907007808441	-0.01085340843924
C	18.79888679750480	0.40140139234597	0.03386318689964
N	20.10551572395957	0.08318892133489	0.00773610133574
C	20.92435342988601	1.23387796976863	0.06292908192784
C	20.10611194482869	2.35879292545814	0.12537057744597
C	18.65381232188501	1.92480568627641	0.11340176558409
C	20.67062117232721	3.62237093275454	0.18631227273288
C	22.06343215080645	3.73866618942504	0.18390625151635
C	22.86857299768630	2.60218183384706	0.12183245541636
C	22.30944945642876	1.32436260186585	0.06017929505331
C	20.61339324551561	-1.27228492828975	-0.06762219252285
C	17.93938447980459	2.49226479205301	-1.12720736601087
C	17.95948887793429	2.36112832637683	1.41700618187295
H	11.61110720427827	0.05606201922033	0.05588756016882
H	12.97364582892820	-2.70628096957567	-0.13151139333039
H	13.94076957401114	0.21781462176824	0.05861634268886
H	16.08511579281732	0.67938333847350	0.07030015653098
H	18.13119842960907	-1.61377457323285	-0.06868077212444
H	21.70265741480610	-1.25208879277839	-0.10198523748453
H	20.30315191089392	-1.85269519343762	0.80919251577642
H	20.24738223416694	-1.77154590058901	-0.97188310980300
H	18.43102523380058	2.16652838033060	-2.04971821929914
H	16.89008458335435	2.18671930424030	-1.17437440441246
H	17.97090058811105	3.58675592327003	-1.09734938178891
H	16.91134440456213	2.04997842246512	1.44955815913000
H	18.46656905044017	1.94461033623217	2.29344838236536
H	17.98977015085552	3.45297973269999	1.49860583094490
H	22.95028242627177	0.44811679018426	0.01298296113266
H	23.95138790514117	2.70848714733957	0.12128574317021
H	22.52353051770299	4.72302138172035	0.23122532959427
H	20.04758560477580	4.51391218970944	0.23533293643016
H	8.59117734606214	-3.84142146580392	0.67438903936791
H	7.04570504570269	-3.77715551656480	-0.19090781124216
H	8.56327495591832	-3.72667084386680	-1.10663444755598
H	6.50958143795877	2.19422742123899	0.17700560582524

H	4.12453380877122	1.49871858307509	0.11560754242325
H	3.52053145725609	-0.89292791336228	-0.04849377492399
H	5.26846758732200	-2.63896012595750	-0.15403489728972
H	8.87549826970000	0.33137381213551	2.24568226494219
H	10.30276976228474	1.00303013859652	1.43485883479264
H	8.79131899697114	1.92349248134616	1.47722179307231
H	10.31612157950950	1.18754160142764	-1.18037340854510
H	8.90401538313930	0.62277172408336	-2.09331046649665
H	8.79896831216672	2.09746193840378	-1.12016968716358
H	10.52048006147777	-2.81972783296693	-0.14431516105137

64
molecule **1c**

C	-0.26273194153685	-0.00556224349950	-0.07133512355458
C	-0.13296664198141	-0.00299933704716	1.31013011119050
C	1.11317478319991	-0.00964943539983	1.93060712603453
C	2.27155694712111	-0.01771178351731	1.17347742425323
C	2.15953322139238	-0.01947638399849	-0.22135767199411
C	0.90606666704207	-0.01368912363667	-0.83394883745101
C	0.92643439300565	-0.00649205138559	3.43508107647760
C	-0.60297057790874	0.00084145639989	3.54408892061566
N	-1.13386998797795	0.00567481112549	2.30392946247628
C	-1.39779170841735	0.00150919378018	4.68594223173437
C	-0.94513614728490	0.00068520008884	6.00954051382912
C	-1.77270967956347	0.00162430003855	7.12624755187338
H	-2.85398163788891	0.00289110925010	6.98173382332235
C	-2.55511276189541	0.01519954526524	2.02386054710847
C	1.54478068216321	1.26581902576572	4.04384610532021
C	1.53443775166236	-1.28132266558816	4.04836522150032
C	-1.27083277787133	0.00112793868997	8.42693585659803
C	-2.03569665796333	0.00251446120237	9.59255407335421
C	-1.46313528048677	0.00224073557154	10.85891550434104
C	-2.18577932729610	0.00355720952312	12.05633329233380
C	-1.65020624693298	0.00364495800687	13.34022335270808
N	-2.43086593161097	0.00304497346466	14.44053013859382
C	-1.66217189811263	0.00641770930927	15.62288248355734
C	-0.31311878180554	0.00708227816879	15.27910517842431
C	-0.17821119088442	0.00511945059086	13.76910549488698
C	-2.07985329352135	0.00905628142035	16.94611647616781
C	-1.09789237406614	0.01171171881055	17.93766175338315
C	0.25675269645304	0.01188364833203	17.60308887552218
C	0.65983333627972	0.00965243199507	16.26300413228181
C	-3.87914518309169	0.00064281188672	14.41389853886995
C	0.55119281251389	-1.26857492202998	13.30293559693234
C	0.54938399499240	1.27873600416448	13.29989366925018
H	-0.37489974500195	0.00075381981879	10.90098498842426
H	-3.12313657637279	0.00381415313877	9.50514805992659
H	-0.18322064092085	-0.00038735800251	8.54299802573521
H	0.12727528439931	-0.00053225140175	6.20093273294928
H	-2.47646794360293	0.00223841494584	4.54423806451400
H	-2.71423640098956	0.05055346332800	0.94625256844203
H	-3.03545540748598	-0.88922594606259	2.41556435275272
H	-3.03195960706244	0.89501310011598	2.47092301220403
H	1.10097545908483	2.16779505251563	3.61001855935926
H	1.41177617070404	1.30739254128487	5.12902723934830
H	2.61989325160481	1.28636449819209	3.83505448599124
H	1.40424942930042	-1.31641244264682	5.13408048421443
H	1.08123896239960	-2.18132861858772	3.62018858105948
H	2.60873984733251	-1.31297301394441	3.83689426170626
H	-1.22896231222369	-0.00237714720657	-0.56866748195445
H	0.83247885352797	-0.01586433579589	-1.91850934189946
I	3.89303804842238	-0.03153204499786	-1.39842647361733
H	3.25195577712838	-0.02283046694835	1.64507610508348
H	-4.25517447685467	-0.88496117317953	13.88876336601348
H	-4.26305150910604	-0.01653279745764	15.43363152821893
H	-4.26003334484442	0.89986272817080	13.91572275583099
H	1.71765144268224	0.00999887217750	16.00804436069950

I 1.70320917476584 0.01598619972329 19.11877299397651
H -1.39828797190310 0.01387986693362 18.98222586530622
H -3.12915597043444 0.00959305674659 17.22894666002790
H 0.02239750756372 -2.16965499887379 13.63064739251933
H 0.65298928278596 -1.30791835453736 12.21440286677681
H 1.55676299935290 -1.29313973491223 13.73585759239207
H 0.64839087087266 1.31697850495030 12.21106924816326
H 0.02074860661394 2.17978964104675 13.62792898434771
H 1.55595389252144 1.30444208025318 13.73043842701700
H -3.27027221198752 0.00499238446509 11.96598283452878

64
molecule **1d**
C 0.85878105009636 0.00719773452001 16.02638642440253
C -0.18538102350547 0.00671501774456 15.11821175271941
C -1.50419107325895 0.00741627885180 15.56327054386876
C -1.82209403247385 0.00893014530816 16.91373261090742
C -0.76854641645175 0.00947369135858 17.82856119334684
C 0.55733284910177 0.00855741477618 17.39291416966408
C -0.16615303665730 0.00566870560248 13.60254124747082
C -1.66584648268081 0.00608569398226 13.28757055886606
N -2.36032852213628 0.00634111239595 14.44101511277956
C -2.29960411253526 0.00618964659328 12.04599114571540
C -1.67329474833533 0.00411502724272 10.79961340277820
C -2.35034060914823 0.00439623075144 9.58109762321311
C -1.67236850704054 0.00227493458029 8.36900292662939
C -2.18410378756734 0.00201598775317 7.06542599594218
I -4.26611232886447 0.00503297799043 6.75903293066438
C -3.80732941751950 0.00607633702330 14.52533289624003
C 0.52289873343101 -1.26873073190906 13.07993689360059
C 0.52435424994098 1.27859109746014 13.07829979196163
I 2.11331943357778 0.00950345842694 18.79534658891885
C -1.31381435135507 -0.00017899371466 5.97492671188032
C -1.63235066960827 -0.00091924762798 4.61839114563151
C -0.71787119299569 -0.00300005553557 3.56601744728311
C 0.81423418171243 -0.00537324476122 3.61143114558814
C 1.15073497675264 -0.00765640818328 2.13325426758199
C -0.02707140466532 -0.00638188509714 1.39159567069488
N -1.12204477499787 -0.00325808513715 2.28161860406753
C 2.37893168734528 -0.01056429219134 1.49543669219743
C 2.40612273444038 -0.01224169245480 0.09649206981906
C 1.21959507989397 -0.01106107746062 -0.63800138112076
C -0.01923389901558 -0.00812172710878 0.00414554377110
I 4.24762902140223 -0.01669623153676 -0.90225280157842
C 1.36507342322295 1.26891294382113 4.27838274139420
C 1.36138830037777 -1.27957255487908 4.28150307127564
C -2.50995383636517 -0.00112278677616 1.86254608213597
H -0.58494153979612 0.00201175697591 10.75468057878277
H -3.43918981188483 0.00634258634202 9.58394091093843
H -0.57977323040802 0.00052473193272 8.42321414724413
H -0.26166727912197 -0.00136433796821 6.25595867834879
H -2.68758671051308 0.00012608173924 4.36225488283383
H -2.56233052490782 0.00248736932100 0.77372937768405
H -3.02726373641085 -0.89398377995945 2.23209786121685
H -3.02581043759030 0.89022742680045 2.23757781746559
H 0.96250735464255 2.16967055795118 3.80374838689432
H 1.12739016818338 1.31073301323864 5.34540524987216
H 2.45533384001812 1.29321370119573 4.17520630920400
H 1.12449564779474 -1.31761654941497 5.34883868866216
H 0.95556640990980 -2.18035022668976 3.80969012784867
H 2.45148184004444 -1.30771997298281 4.17755532688805
H -0.93150470399204 -0.00738447585968 -0.58606727650472
H 1.25478609391057 -0.01249811758148 -1.72450286645434
H 3.30732455170307 -0.01157885144552 2.06234568910330
H -4.22325536934552 -0.88298129092867 14.03774202118456
H -4.11093082998407 -0.00209692459152 15.57173989590511
H -4.22348136929251 0.90231874548401 14.05105822450013

H	1.89454633518647	0.00665793568730	15.69326587830998
H	-0.98941562556876	0.01072630143793	18.89281494907914
H	-2.84734161735297	0.00992833208206	17.27376839091465
H	0.01839389666121	-2.16971618957990	13.44402098173963
H	0.54210048323391	-1.30499162655390	11.98658364262869
H	1.55816458928614	-1.29681330661420	13.43599349806039
H	0.54389606840974	1.31331801161735	11.98493995498849
H	0.02071774542526	2.18061727715025	13.44100576594215
H	1.55956968076957	1.30609850028643	13.43455696419693
H	-3.38765241312756	0.00797989911853	12.04058812221031

Table S3. Optimized S_1 excited state geometries of **1a**, **1b**, **1c**, and **1d** calculated at CAM-B3LYP/def2-SVP level in the gas phase. The first number (64) is the number of atoms in the molecule. There are no imaginary frequencies.

64
molecule **1a**

C	0.47859745058542	0.01284002313141	16.46469576639716
C	-0.41219381685982	0.00859075466165	15.40902768013209
C	-1.80360747646188	0.00444291973116	15.65542345854620
C	-2.31138959505313	0.00476925820244	16.96251461808498
C	-1.40121609406400	0.00904271431382	18.00909167505751
C	-0.01660329584268	0.01303418970308	17.77289532694815
C	-0.15876719139195	0.00733980485082	13.91538250599641
C	-1.59283114166278	0.00286018386555	13.37523375016180
N	-2.48213457000552	0.00043450566770	14.46082574570119
C	-2.03152921437416	0.00146037909545	12.08467336693820
C	-1.22659403802343	0.00306388410323	10.90067982869806
C	-1.74816829369283	0.00157311060643	9.62527525220862
C	-0.96613359998189	0.00289447155911	8.46000515889782
C	-1.48192893786770	0.00126440521742	7.15515408097701
C	-0.70114610882078	0.00229789634005	6.01951012982608
C	-1.23720209960163	0.00092871399351	4.69200803938431
C	-0.53680842456601	0.00044780531333	3.52254126077656
C	0.97839330535459	-0.00149241952253	3.29503541824549
C	1.04376545136610	-0.00518681430616	1.78136896794235
C	-0.26501294069718	-0.00376151088406	1.24880621503797
N	-1.17876266860844	0.00114795413747	2.27475606311809
C	2.13597311797677	-0.00937366993022	0.93568236794684
C	1.92570998771663	-0.01231910119235	-0.44714860943517
C	0.62128563952565	-0.01123547716144	-0.96829931943370
C	-0.48791817190419	-0.00709591555426	-0.13554757137949
C	1.64973432812979	1.26878192208520	3.85367596011415
C	1.64798735190728	-1.26999663106012	3.85962853444344
C	-2.61077840806575	0.00436999498429	2.12146490367369
C	-3.91462767124967	-0.00468474468846	14.31034778807834
C	0.61741919072928	-1.26070579831936	13.50839970063059
C	0.61101974453514	1.27820256476085	13.50516427765522
H	-2.56820515630702	-0.00105492676191	7.03823669138380
H	-0.14265497742215	0.00541899604713	11.00014281922537
H	-2.83435486035935	-0.00084440772426	9.50761406248278
H	0.12054745894807	0.00532971122032	8.57741933838123
H	0.37939840264845	0.00472531491958	6.15377186632926
H	-2.32447798854086	0.00036592048805	4.62851041559263
H	-2.87158873388636	0.01571501080595	1.06297011617174
H	-3.05592937311870	-0.89027818753463	2.57727592693179
H	-3.05229889283637	0.89166608642003	2.59447348775653
H	1.17536117367500	2.17251407536150	3.45762457402482
H	1.59713284767018	1.30741995215578	4.94599501975362
H	2.70730542465044	1.28776222067975	3.56766538433392
H	1.59600531061358	-1.30329558785815	4.95211771394345
H	1.17204987425434	-2.17491682696348	3.46819595425651
H	2.70537831603901	-1.29199581335135	3.57318103954380
H	-1.48994627806540	-0.00687472622340	-0.55508625587058
H	0.47717655720725	-0.01394013325579	-2.04676768650900

H	2.77566947573407	-0.01565324660989	-1.12545261332969
H	3.14969152658784	-0.01044274373541	1.33278949605895
H	-4.24668286994340	-0.89487532852315	13.75949940789113
H	-4.39178265478765	-0.01123879470471	15.29061026845777
H	-4.25456033020687	0.88728205134945	13.76711477832924
H	1.55326196419676	0.01603950140557	16.28857154088777
H	0.67220088017920	0.01636211281392	18.61436365139432
H	-1.76791059849358	0.00940331815912	19.03327990954923
H	-3.37915751982935	0.00197512989045	17.16344741100368
H	0.07234478971009	-2.16596822651828	13.79530213954106
H	0.79485028447429	-1.29780832302186	12.42962462029874
H	1.59141211252162	-1.27797395903214	14.00940350129354
H	0.78849718539509	1.31340135701364	12.42634122274105
H	0.06134003301831	2.18144761766362	13.78961108476260
H	1.58483882327388	1.30168963000986	14.00626520063790
H	-3.10770201603161	-0.00119114829036	11.91698557138061

64

molecule **1b**

C	6.09139556445440	1.00480390651170	0.09805902255164
C	7.12090128975457	0.08599266677059	0.04286082720646
C	6.83451352618227	-1.29647746825743	-0.05195165160684
C	5.51227322573904	-1.76472515785386	-0.09389072487232
C	4.49344547579821	-0.82676178694562	-0.03769547612117
C	4.76975855758453	0.54867379410476	0.05796674097567
N	8.00666140444060	-2.00768732944610	-0.09063921802400
C	9.11912263499830	-1.14940094526896	-0.03063362264262
C	8.62085388051335	0.29632817861424	0.06623011331787
C	10.39543950719993	-1.62574446309103	-0.06291334376968
C	11.60258297920219	-0.85961558460219	-0.00805203633526
C	12.86018023487146	-1.42578900906764	-0.05451196989413
C	14.03815916749610	-0.67166341496178	-0.00403368145248
C	15.37661180892997	-1.11041420120861	-0.04119826486314
I	15.76278428107545	-3.18051101351546	-0.18060964979554
C	9.04410126882013	0.96848250920098	1.38750952786999
C	9.05820746200735	1.14339513658882	-1.14517143036015
C	8.11798697857055	-3.44083596939946	-0.18446959526317
C	16.42683140394891	-0.20941341772655	0.01136602987925
C	17.82137788153233	-0.48665235322937	-0.01248880660424
C	18.83431252769971	0.43392766152608	0.03210756819164
N	20.17128479385772	0.04761796082143	0.00384362184547
C	21.00905717145702	1.14244701765280	0.05982232594463
C	20.23656463791992	2.31952851639840	0.12558087878532
C	18.76457493474661	1.96197300636102	0.11391345679186
C	20.85657671435148	3.55319268264308	0.18867064615961
C	22.25366502443715	3.61141024723754	0.18611569926057
C	23.01417513280719	2.43435777128840	0.12129871358600
C	22.40827126078798	1.18665707532438	0.05756107302711
C	20.60009151271951	-1.32752809113258	-0.07124382627110
C	18.07743257214193	2.57582135286693	-1.12180961301911
C	18.09146784762784	2.44164999767366	1.41519607698611
H	11.53650668330807	0.22382682549742	0.07498562296105
H	12.93964771828445	-2.50972372066073	-0.13617924003389
H	13.92239574636169	0.41272466271696	0.07493871252111
H	16.11398987850195	0.83121331270627	0.07866903575554
H	18.09933358145432	-1.53536024808760	-0.07309493455216
H	21.68918353730011	-1.37530789743630	-0.09859227116460
H	20.24946675163971	-1.89503509542239	0.80095055731632
H	20.21047226737905	-1.80778586645883	-0.97810873725618
H	18.55046458419959	2.23228191566407	-2.04744877422978
H	17.01565661837378	2.31587023498980	-1.16700590260189
H	18.15518052562218	3.66828731738336	-1.08563790257766
H	17.03136283546339	2.17296031446016	1.44700030136531
H	18.57820235295821	2.00752085180381	2.29445320838845
H	18.16430157814869	3.53238304202961	1.49070859182371
H	23.01181402075206	0.28484487797048	0.00852554551857
H	24.10032721315319	2.49922099582987	0.12096442906974

H	22.75661561164654	4.57440102984282	0.23524518696342
H	20.27240912133142	4.47033394306451	0.24000016934392
H	8.66596270184602	-3.84657083992352	0.67628315873993
H	7.12514734313725	-3.89099578724932	-0.20091461795037
H	8.64553087953352	-3.73402059754559	-1.10218755409656
H	6.29793580080139	2.07137796268950	0.17201765128750
H	3.94806323961988	1.25969254461433	0.10038364529053
H	3.45891079960227	-1.16165209824621	-0.06875037575552
H	5.28109240904486	-2.82353502149302	-0.16893816121789
H	8.73899346390344	0.36896049141803	2.25132329747340
H	10.12727019820717	1.11257899653874	1.43878125901893
H	8.57067196676844	1.95278685717607	1.47308488156846
H	10.14154658337718	1.29432189934411	-1.16358357036332
H	8.76465070523199	0.66731978207606	-2.08646471263369
H	8.58335582885772	2.12960166296159	-1.10109354582744
H	10.53131179051563	-2.70351062413220	-0.14022836562926

64

molecule **1c**

C	-0.49413602083139	-0.01902578626982	-0.13353216599365
C	-0.27021917166962	-0.00902809545144	1.24957181502764
C	1.03945810134774	-0.01437075184591	1.77278639940399
C	2.12875327299235	-0.02836224512295	0.93100055472800
C	1.91272186182283	-0.03751351962817	-0.45750389456413
C	0.60714768371227	-0.03288978375093	-0.97635961147971
C	0.98199945634341	-0.00212952423288	3.28868737127558
C	-0.53262525471152	0.00393786679506	3.52322276821040
N	-1.17970781934671	0.00626083459972	2.27779960752109
C	-1.22604306297889	0.00568438528460	4.69585287969779
C	-0.68683694999984	0.00936476958221	6.02025349055468
C	-1.46469659770639	0.00436208762146	7.15824587104785
H	-2.55114734390298	-0.00396418700628	7.04245768850470
C	-2.61272144525633	0.01722868832484	2.13009957918057
C	1.65524862755036	1.27125761820860	3.83613557083296
C	1.65152169247193	-1.26781615843145	3.85711637270581
C	-0.94936575196919	0.00855602629392	8.46238403881669
C	-1.73202751347024	0.00221243855933	9.62621146033011
C	-1.21480191508562	0.00567727868424	10.90371340092267
C	-2.02250390286485	-0.00189347962331	12.08370410323410
C	-1.59096584485271	0.00041738011132	13.37581515010467
N	-2.48413619005947	-0.00970743158304	14.45847879868033
C	-1.80951551369823	-0.00132529663614	15.65412572827412
C	-0.41941483587576	0.01244762345716	15.41614860611555
C	-0.15870689047819	0.01379829578854	13.92188005734200
C	-2.31696418993435	-0.00477762378762	16.96024590278563
C	-1.41575121350868	0.00498881337390	18.01431334804914
C	-0.03060881346514	0.01832971477164	17.77949294092245
C	0.47025054355314	0.02220161697162	16.46667987898003
C	-3.91648416830421	-0.02582604485231	14.30296334189975
C	0.62542748880293	-1.24931425165672	13.51765021140060
C	0.60291730343375	1.29003034109911	13.51604698257225
H	-0.13116754119216	0.01415313905300	11.00518737301466
H	-2.81803267164097	-0.00642431075321	9.50646376488520
H	0.13706813310178	0.01701937259752	8.58035539310632
H	0.39379168614409	0.01792196353672	6.15299775412144
H	-2.31360962895565	0.00436485769396	4.63612825795547
H	-2.87805652651416	0.04699526825310	1.07288256358629
H	-3.06116369975319	-0.88206098466824	2.57319988607275
H	-3.04799779723846	0.89937001943251	2.61756936159156
H	1.18358810176002	2.17227626703160	3.43072680461726
H	1.59793575404855	1.31996294704721	4.92770115170200
H	2.71418504553121	1.28606167880563	3.55432077238298
H	1.59806262184756	-1.29687683273981	4.94945627952535
H	1.17512839227064	-2.17409497515269	3.46935151194395
H	2.70947698353542	-1.29193387982043	3.57234314530364
H	-1.49558988455982	-0.01724094717771	-0.55435486339840
H	0.45492552427690	-0.04100858122287	-2.05276050109687

I 3.53235314133545 -0.05876095369146 -1.75463662466972
H 3.14210239850428 -0.03248612821469 1.32688442618595
H -4.23890631072673 -0.91448008798549 13.74442175372372
H -4.39741868086551 -0.04510344689733 15.28142321624543
H -4.26316024547575 0.86741049215064 13.76645064046635
H 1.54400553958784 0.03278269092940 16.29096977109988
I 1.28203661535503 0.03259076333768 19.38666366596533
H -1.78931095790659 0.00245316219321 19.03517327982797
H -3.38423457466333 -0.01444792231323 17.16342715491224
H 0.08616404670262 -2.15746158263109 13.80651026147647
H 0.80045717611533 -1.28726856896938 12.43867134868111
H 1.60081415127443 -1.26016577639883 14.01643207952061
H 0.77565306932577 1.33068390372067 12.43682065773150
H 0.04858890710062 2.18886977019916 13.80545574283677
H 1.57874736441232 1.31776812939254 14.01335038458523
H -3.09812375479794 -0.01070404638637 11.91233133901308

64
molecule **1d**
C 0.71964969712745 0.00901060131061 16.21771161181683
C -0.26200419362061 0.00736181404632 15.25298615789074
C -1.62506137548709 0.00600419795267 15.61731549689483
C -2.01192886295699 0.00658919970587 16.96447604343626
C -1.01862789891345 0.00821086721596 17.93156242558794
C 0.34010117759607 0.00936546097699 17.57095408442296
C -0.13907733606792 0.00666207037057 13.74119415661477
C -1.61520461817202 0.00544566686227 13.32967957451310
N -2.40604976613687 0.00407342404953 14.48857447101526
C -2.16555113338598 0.00562198174403 12.08221617001230
C -1.47454336245723 0.00512369916465 10.83336451565149
C -2.12023250052120 0.00554500706832 9.61236280825544
C -1.44493837632246 0.00424948234777 8.38875292200515
C -1.97408133392728 0.00420096554880 7.08316626020122
I -4.07247094959574 0.00727053003990 6.83335366489120
C -3.84715894066244 0.00179330102822 14.46452484789708
C 0.59425797403911 -1.26284183002880 13.26712071778502
C 0.59279644814381 1.27653239159387 13.26589575092423
I 1.79261757521910 0.01178393605404 19.05070775035027
C -1.14740444490401 0.00202728114286 5.97451353361764
C -1.52234777417125 0.00143352892658 4.60130206403395
C -0.67882374979685 -0.00152543916458 3.52695695212715
C 0.85313269026435 -0.00540781971388 3.48397218097584
C 1.10018131000360 -0.00890226837035 1.98746015451687
C -0.13288573689902 -0.00656071979713 1.30602354314016
N -1.16392403290026 -0.00172754055147 2.21690041923050
C 2.28643766768499 -0.01367278980899 1.28746778149509
C 2.24363439392169 -0.01623110481638 -0.11640016449405
C 1.01307762349959 -0.01401015060155 -0.79306404350481
C -0.18484626295813 -0.00920535629017 -0.09310063373461
I 4.01261726485352 -0.02342945627290 -1.20394711755894
C 1.45181756036059 1.26464690710934 4.11843510415118
C 1.44573647768514 -1.27582697989214 4.12333676838608
C -2.56927546017014 0.00174514601355 1.89205469334551
H -0.38590498536038 0.00390557575461 10.82972936208071
H -3.21019950558114 0.00676611750233 9.60344724643099
H -0.35238722869102 0.00283033124207 8.43122787447033
H -0.08548362045932 0.00055063689470 6.21539398014537
H -2.58997070509552 0.00334866126659 4.39924664972780
H -2.70081820243534 0.00439698339997 0.80947980694967
H -3.06643951685870 -0.88865689652340 2.29853530581073
H -3.06238298227149 0.89270458855177 2.30209091283702
H 1.03260265567483 2.16804181945202 3.66383105998577
H 1.26057868358440 1.30753388163869 5.19482971253035
H 2.53744653204673 1.28108074017125 3.96895693541549
H 1.25465599100774 -1.31350809893149 5.19995498429576
H 1.02200935678022 -2.17896505863154 3.67242515714670
H 2.53122659682644 -1.29816993426343 3.97364492559468

H	-1.12643296694850	-0.00773582355869	-0.63467827506158
H	0.99554151993767	-0.01616487652580	-1.88011639540288
H	3.24289621609198	-0.01548319395104	1.80568283761324
H	-4.22813246322836	-0.88716223231492	13.94488328025123
H	-4.23668067235807	-0.00512691960858	15.48293088060671
H	-4.23193195878851	0.89502449631936	13.95496607234554
H	1.77297271650900	0.01004301250835	15.94520097332850
H	-1.29705654587807	0.00869869700102	18.98233878623105
H	-3.05623673007666	0.00598820454127	17.26334325661974
H	0.07710718702114	-2.16687124469488	13.60483744534875
H	0.66877952609502	-1.30174917351815	12.17641977751787
H	1.61158809966171	-1.28147035184721	13.67358243745344
H	0.66751816922572	1.31443722214750	12.17520115241865
H	0.07449493467811	2.18028341421970	13.60258851140318
H	1.61003079227355	1.29681528692096	13.67252380804962
H	-3.25251964375520	0.00576012987255	12.01219387196099

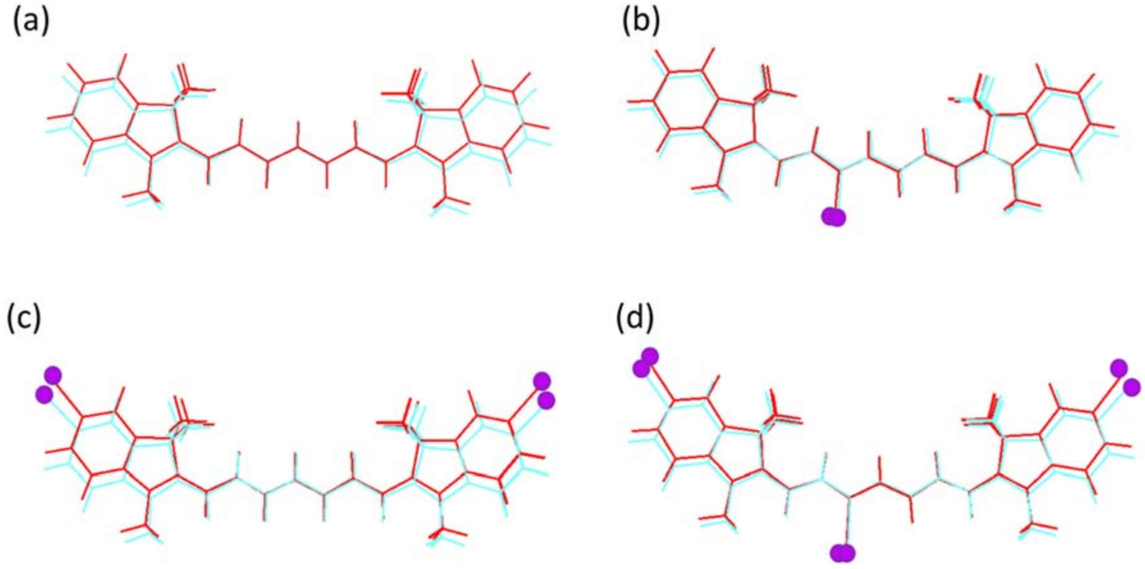

Figure S46. Overlap between the S_1 excited (turquoise) and ground state (red) optimized geometries of **1a** (a), **1b** (b), **1c** (c), and **1d** (d) calculated at CAM-B3LYP/def2-SVP level in the gas phase. The cyan circle shows the iodine atom.

Table S4. Excitation energies in eV (nm) for molecules **1a–d** calculated for S_1 minimized structures at the TDDFT DKH CAM-B3LYP/def2-TZVP level in water as a polarizable continuum with or without Tamm-Dancoff approximation.

Cy7	TDDFT S_1	TDA S_1	TDDFT T_1	TDA T_1	TDDFT T_2	TDA T_2
**1a**	2.176 (570)	2.354 (527)	0.718 (1727)	1.100 (1127)	2.010 (617)	2.140 (579)
**1b**	2.173 (571)	2.345 (529)	0.808 (1534)	1.144 (1084)	1.984 (625)	2.128 (583)
**1c**	2.141 (579)	2.308 (537)	0.727 (1705)	1.107 (1120)	2.017 (615)	2.139 (580)
**1d**	2.135 (581)	2.295 (540)	0.815 (1521)	1.148 (1080)	1.983 (625)	2.120 (585)

Table S5. SOCMEs in cm^{-1} for **1a–1d** calculated for the S_1 optimal structures at the DKH CAM-B3LYP/def2-TZVP and def2-SVP levels in water as a polarizable continuum.

	SOCME T_2-S_1		SOCME T_1-S_1	
Cy7	def2-TZVP	def2-SVP	def2-TZVP	def2-SVP
**1a**	0.010	0.010	0.000	0.000
**1b**	0.432	0.425	0.054	0.054
**1c**	0.037	0.030	0.010	0.010
**1d**	0.000	0.000	0.000	0.000

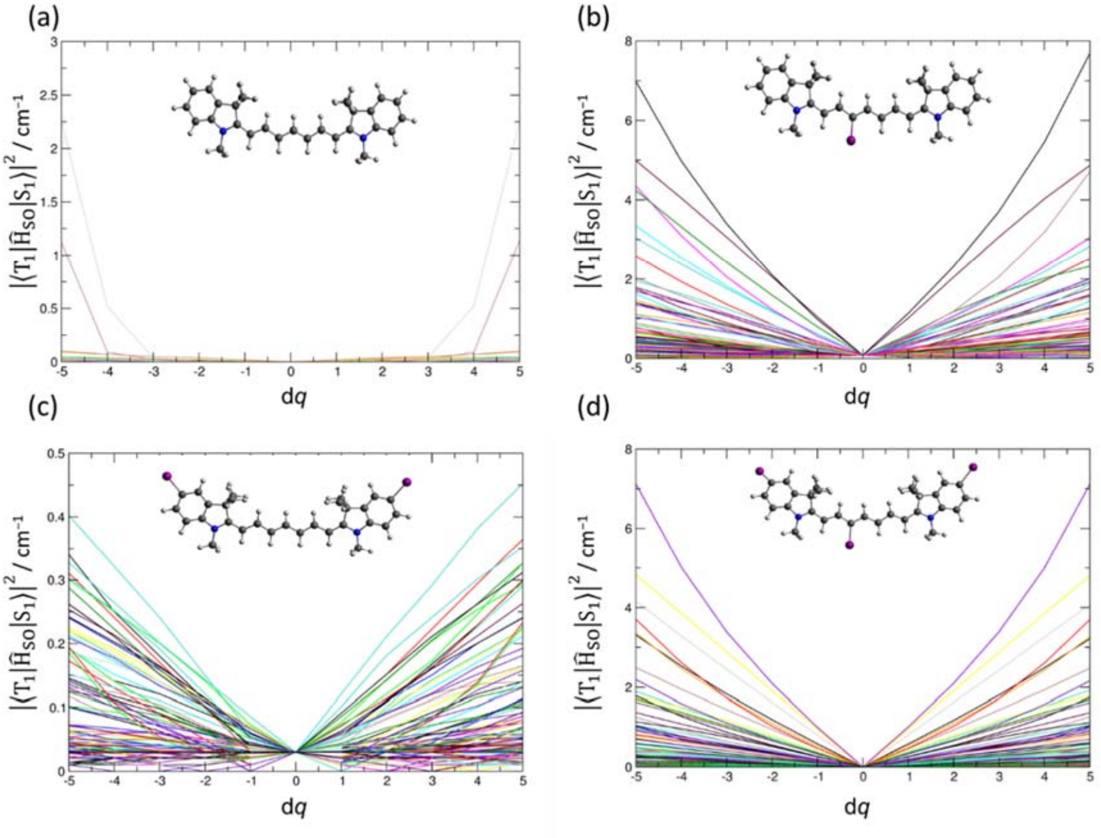

Figure S47. Spin-orbit coupling matrix elements (SOCMEs) at the DKH CAM-B3LYP/def2-SVP level in water as a polarizable continuum between the T_1 and S_1 states for **1a** (a), **1b** (b), **1c** (c), and **1d** (d) along all normal modes q_i. The dq values are given in units of a dimensionless normal-mode coordinate displacement.

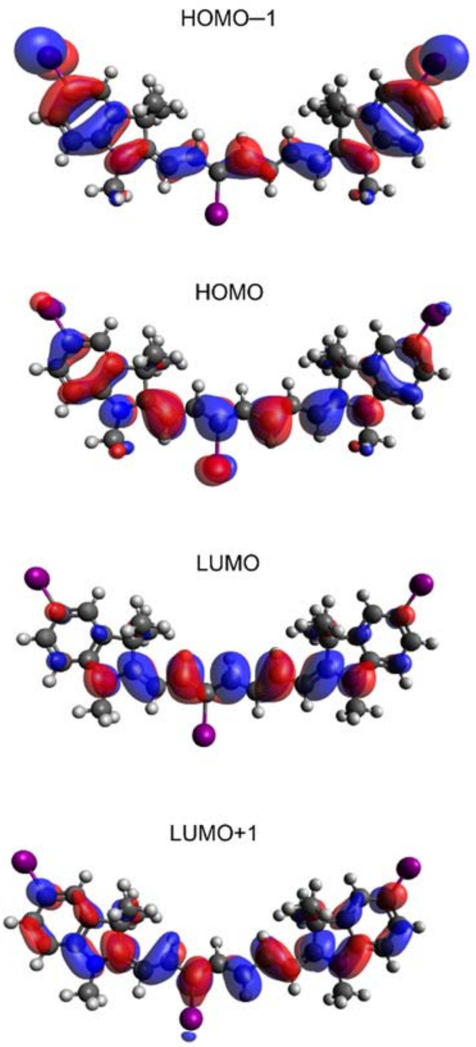

Figure S48. Frontier molecular orbitals for **1d** for dq = 5 (the values are given in units of a dimensionless normal-mode coordinate displacement) along an out-of-plane normal mode ω_{80} (941.93 cm^{-1}) at CAM-B3LYP/def2-SVP level. The contour threshold is set to 0.02 a.u. to better illustrate the contribution of orbitals on the iodine atom (C3′ position).

Table S6. SOCMEs in cm^{-1} for **1b** calculated for the optimal structures at the DKH CAM-B3LYP/ def2-TZVPP, def2-TZVP, and def2-SVP levels in water as a polarizable continuum.

	SOCME T_2-S_1			SOCME T_1-S_1		
Cy7	def2-TZVPP	def2-TZVP	def2-SVP	def2-TZVPP	def2-TZVP	def2-SVP
**1b**	0.071	0.244	0.472	0.020	0.037	0.079

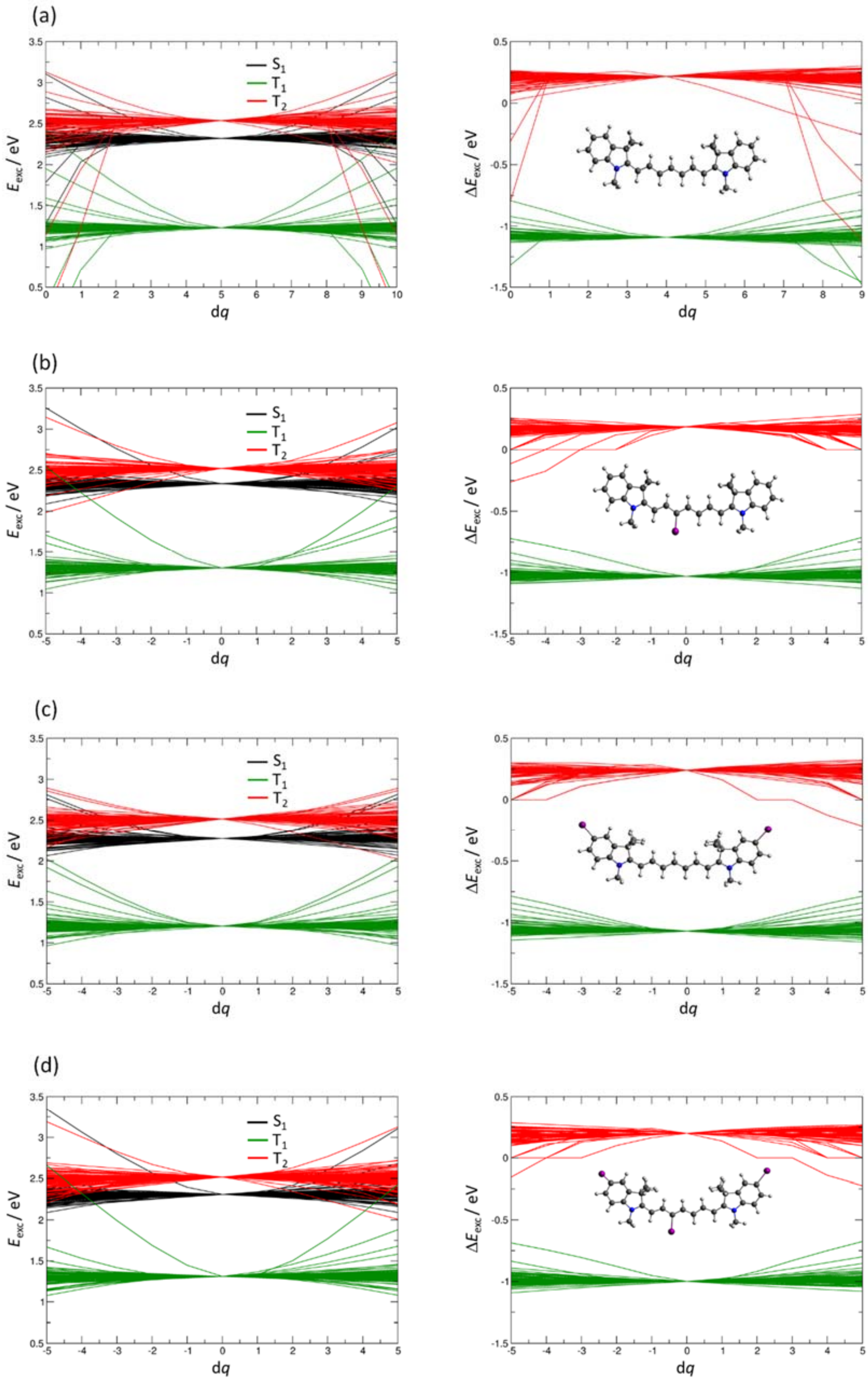

Figure S49. TDDFT energies at the DKH CAM-B3LYP/def2-SVP level in water as a polarizable continuum of the S_1, T_1, and T_2 states and energy differences between the S_1 and T_1 and S_1 and T_2 states along all vibrational modes for **1a** (a), **1b** (b), **1c** (c), and **1d** (d).

(a) Starting material (**1b**)

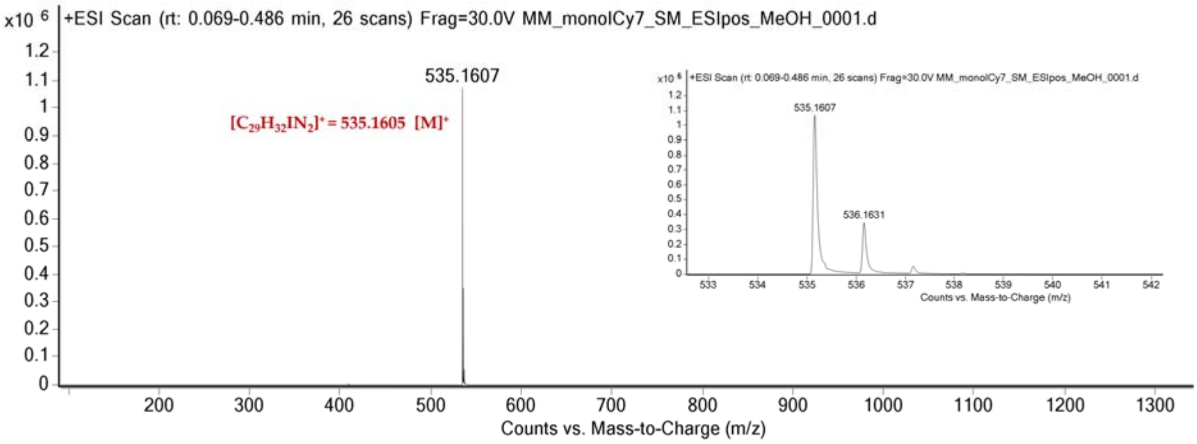

(b) Irradiated mixture

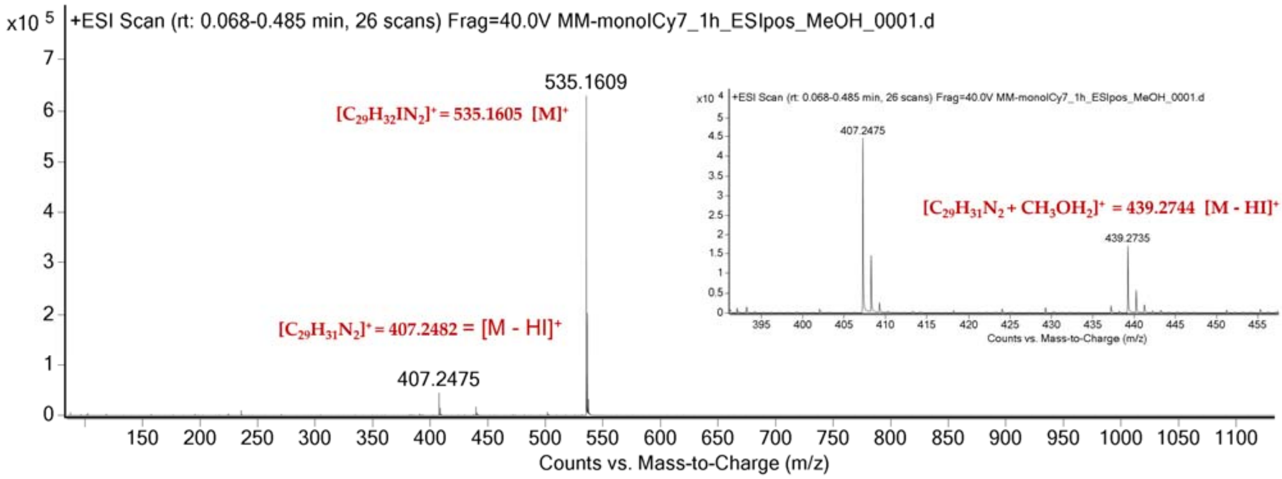

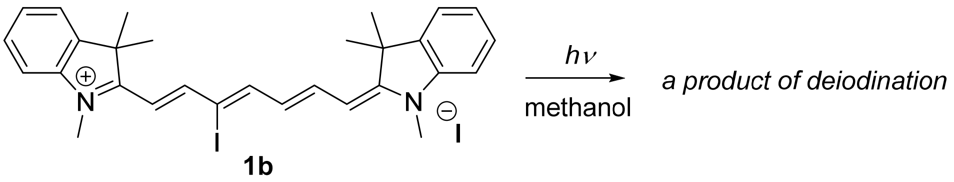

Figure S50. Irradiation of **1b** in methanol (c(init) = 5 × 10^{-6} M) using 700 nm LEDs: (a) **1b** before irradiation; (b) the formation of a product of deiodination (accompanied by the loss 2 H atoms) as a major detectable photoproduct (at ~50% reaction conversion).

(a) Starting material (**1c**)

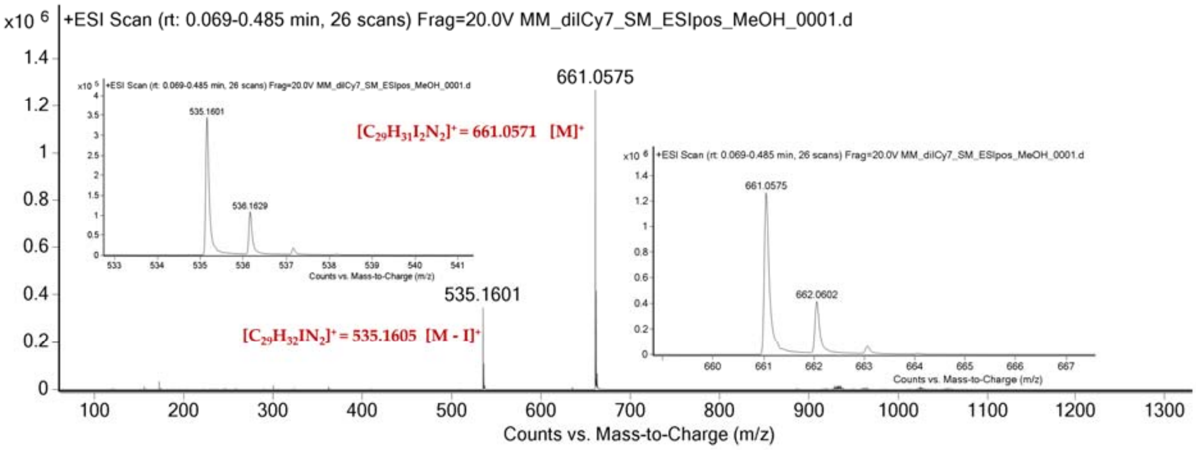

(b) Irradiated mixture

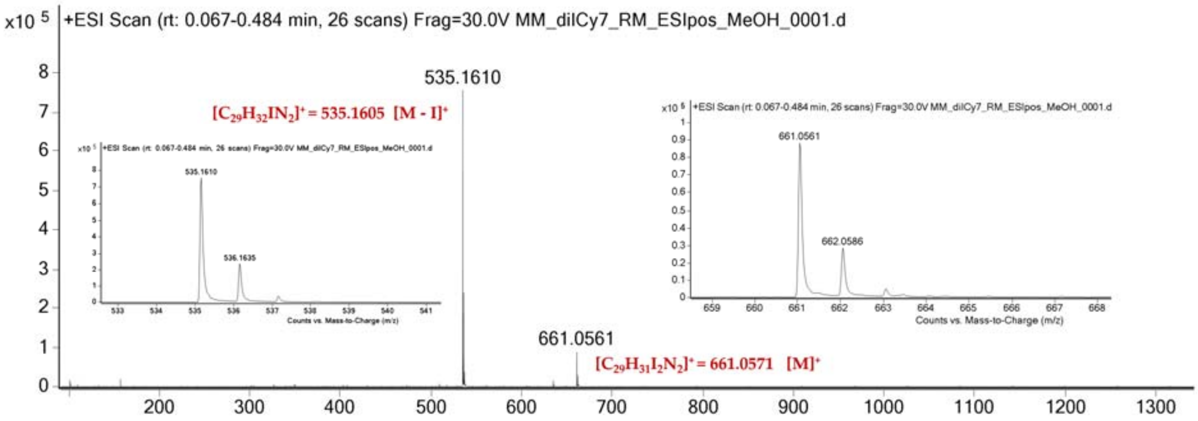

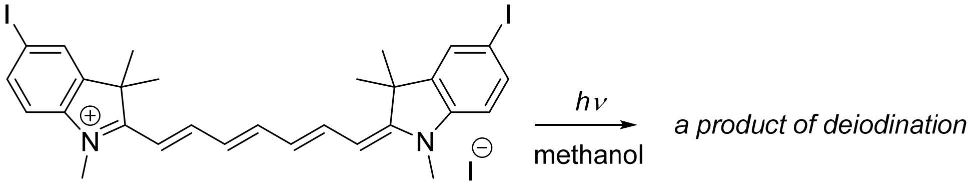

Figure S51. Irradiation of **1c** in methanol (c(init) = 5 × 10^{-6} M) using 700 nm LEDs: (a) **1c** before irradiation (partial deiodination detected during the MS analysis); (b) the formation of a monodeiodinated Cy7 as a major detectable photoproduct (at a complete reaction conversion).
